# On growth and form of animal behavior

**DOI:** 10.3389/fnint.2024.1476233

**Published:** 2025-02-04

**Authors:** Ilan Golani, Neri Kafkafi

**Affiliations:** School of Zoology, Faculty of Life Sciences, Tel-Aviv University, Tel Aviv, Israel

**Keywords:** ethology, behavioral phenotyping, comparative anatomy, evo-devo, behavioral homologies, mobility gradient, dopaminergic system, Eshkol-Wachman movement notation

## Abstract

In this study we propose an architecture (*bauplan*) for the growth and form of behavior in vertebrates and arthropods. We show in what sense behavior is an extension of anatomy. Then we show that movement-based behavior shares linearity and modularity with the skeletal body plan, and with the Hox genes; that it mirrors the geometry of the physical environment; and that it reveals the animal’s understanding of the animate and physical situation, with implications for perception, attention, emotion, and primordial cognition. First we define the primitives of movement in relational terms, as in comparative anatomy, yielding homological primitives. Then we define modules, generative rules and the architectural plan of behavior in terms of these primitives. In this way we expose the homology of behaviors, and establish a rigorous trans-phyletic comparative discipline of the morphogenesis of movement-based behavior. In morphogenesis, behavior builds up and narrows incessantly according to strict geometric rules. The same rules apply in moment-to-moment behavior, in ontogenesis, and partly also in phylogenesis. We demonstrate these rules in development, in neurological recovery, with drugs (dopamine-stimulated striatal modulation), in stressful situations, in locomotor behavior, and partly also in human pathology. The buildup of movement culminates in free, undistracted, exuberant behavior. It is observed in play, in superior animals during agonistic interactions, and in humans in higher states of functioning. Geometrization promotes the study of genetics, anatomy, and behavior within one and the same discipline. The geometrical *bauplan* portrays both already evolved dimensions, and prospective dimensional constraints on evolutionary behavioral innovations.

## 1 Introduction

A common denominator marks the current literature on the analysis of animal and human movement-based behavior: on the one hand, the community is “brimming with excitement and boundless opportunity” being overwhelmed by the big kinematic data offered by novel computational technologies of machine vision and deep learning (von Ziegler et al., 2021). On the other hand, it witnesses a plethora of novel phenotyping methods of automated behavior classification and discovery, based on novel technologies borrowed from machine vision and feature extraction (Berman et al., 2014; Egnor and Branson, 2016; Calhoun and Murthy, 2017). Perhaps because in the vast majority of these studies the main interest is in the processes that mediate behavior, rather than in the growth and form of behavior in and for itself (as was the case with comparative anatomy before the discovery of genetics and neurophysiology), automated behavior classification and discovery largely focus on species-specific behavior, disregarding the phyletic and cross-phyletic perspective.

This essay offers a solution to the situation by describing common primitives and modules, by describing the organization of movement in vertebrates and arthropods, and by providing a comprehensive methodology for revealing and confirming this organization.

The methodology we offer presents a rebirth of several old, yet radical insights largely ignored in the study of movement-based behavior. The first, *organismic* or *holistic* insight is that the organization, including the primitives (natural elementary building blocks), modules (locally integrated units) and generative rules of the different levels of the pyramid of life cannot be defined separately and independently of the organism as an indivisible whole, without initial reference to the organization at other levels, and to the organism’s situatedness in the environment during all its developmental stages. The second, *ethological*, insight is that behavior is an extension of anatomy. It has been conceived by classical ethologists, hardly pondered upon, and abandoned. One implication of this insight is that the connectedness of the skeleton, with its specific mechanical constraints, is mirrored in movement. The other insights are structural. They have been central to developmental biology and evolutionary developmental biology (dubbed evo-devo), but shunned by ethologists, behavioral neuroscientists, and current day phenotypers of movement-based behavior. One such insight is that portraying growth and differentiation (*morphogenesis*) is essential for understanding the process through which a single-celled egg gives rise to a complex, multi-celled sentient, cognizing, and functioning organism. Another, *continuity* insight, is that “an organism’s skeleton begins as a continuum, and a continuum it remains all lifelong” (d'Arcy, 1942; Gould, 1971). Movement grows and differentiates continuously, much like living tissue, and *not* as a sequence of discrete, fragmented, units typically reported by classical ethologists and computational phenotypers (e.g., Berman et al., 2014; Datta et al., 2019; Ligon et al., 2019). Still another, *connectedness* insight is that a part of the body of an organism belonging to one species is homologous to a part belonging to another organism of another species, if and only if their connectedness to other parts of their respective bodies is identical (as opposed to the two parts having a similar shape). We use this so-called *principle of connections*, implemented by Saint Hilaire for the identification of anatomical homologies (Saint-Hilaire, 1830), for the definition of movement-based *behavioral homologies*.

Ethologists, behavioral neuroscientists and phenotypers either ignore, or take lightly, or adopt the opposite, reductionist views in the study of animal movement. Because of the above lacunae, the recording, presentation, analysis, and understanding of movement, being the primary component of behavior, has lagged behind the study of the corresponding molecular, genetic, and anatomical levels, for well over 50 years. The present work aims to fill these lacunae by creating a framework for implementing the holistic, morphogenetic, continuity, and connectedness principles in the study of the structure (geometry) of movement-based behavior.

**Figure 1:**
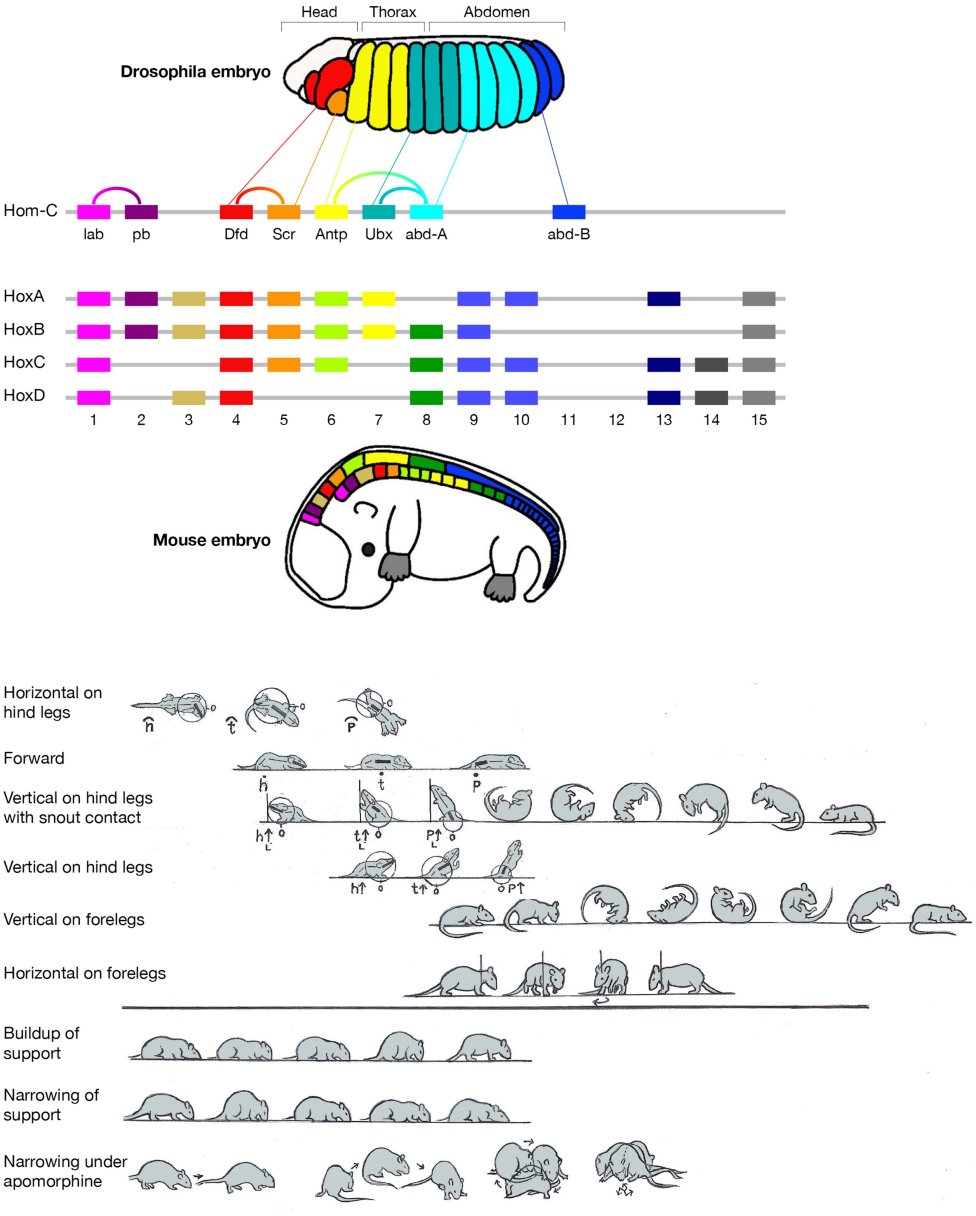
Schematic pictorial summary of the vertebrate *bauplan*: Top: The linear Hox genes’ organization on the chromosome(s) corresponds to their linear, modular, transcriptional expression along the Anterior–Posterior (AP) axis in an arthropod (fruit fly embrio) and a tetrapod (mouse embrio) (Adapted from Stefanie D. Hueber, Georg F. Weiller, Michael A. Djordjevic, Tancred Frickey, https://commons.wikimedia.org/wiki/File:Genes_hox.jpeg). consolidating into a kinematic linkage of multiple, specialized, rigid segments, bottom. This so-called collinearity between genes and the modular anatomical morphogenesis of the body plan is also manifested in a locomotor *bauplan*, unfolding on the temporal scales of moment-to-moment behavior (actualgenesis), ontogenesis, and partly also vertebrate phylogenesis. In vertebrates, locomotor behavior unfolds along six modular spatial dimensions, emerging successively in a fixed order and added progressively to the animal’s repertoire. The build-up is linear within each module separately, progressing from fore- to hind quarters. Pivoting and rearing on the hind legs emerges early in locomotor morphogenesis (top 4 horizontal lines) and pivoting and rearing on the forelegs emerges late (5th and 6th horizontal lines). In stressful situations and under the influence of dopaminergic drugs the spatial locomotor modules are eliminated from the animal’s repertoire in reverse order to their emergence, “last in first out.” Also, the support module is inactivated in a linear tail-to-head order (PA), restricting kinematic freedom.

To introduce the morphogenetic study of behavior we start with the primitives, modules, and geometric rules that have been isolated by evo-devo in vertebrates and in arthropods, and applied at the genetic and anatomical levels (Figure 1 top). Then we proceed to describe their counterparts in the study of movement. The primitives at the genetic level are genes, and their respective modules are gene regulatory networks (Wagner, 2007). The primitives at the skeletal level are bones in vertebrates and rigid exoskeleton segments in arthropods, and their respective modules are organs and the main axes of the body.

Evo-devo shows that the morphogenesis of skeletal anatomy unfolds in reference to the main, Antero-Posterior (AP), Dorsi-Ventral (DV), and Proximo-Distal (PD) axes of the body. These axes are not extrinsic, arbitrarily superimposed geometric constructs, but rather real, active, partly independent physiological morphogenetic fields, now termed modules (Child, 1941; Opitz, 1985; Wagner et al., 2007). The demarcation of the anatomical *body plan’s* main axes is one of evo-devo’s ways to impregnate, right from the start, the primitives, modules, and generative rules of morphogenesis with holistic features even when dealing with local processes. The term body plan refers to a set of morphological features characterizing the members of a phylum of animals.

In our studies of animal movement, the primitives, modules and architectural plan of movement are induced by the anatomical body plan. Therefore, they critically mirror the mechanical connectedness imposed by the kinematic linkage of segments. The primitives are circular movements of single rigid segments moving within individual spheres schematically centered on the respective joint closer along the linkage to the base of support. A change in the base of support may entail a shift of the sphere’s center to the opposite joint, reflecting a respective reversal in the mechanical hierarchy between the segments. The coordinate systems of the individual spheres are gyroscopically oriented in reference to the absolute frame. Describing movement in reference to both the base of support and the absolute physical environment secures a relevant, situated (being embedded in the environment), and being embedded in the body description.

As in evo-devo, isolating primitives that are defined by their connectedness to other primitives, and by their relative position within the whole kinematic linkage, as well as by their orientation in reference to gravity and to absolute and body-related spatial direction, secures the global coherence of behavior, yielding natural kind (Wagner, 1996; Stanford Encyclopedia of Philosophy, 2022) primitives, modules, and generative rules, all permeated from the start with organismic features.

In contrast, classical ethologists and current computational phenotypers of behavior typically employ a reductionist approach. They first establish elementary building blocks, focusing exclusively on their intrinsic features and ignoring their extrinsic connectedness, and their being parts of an indivisible whole, be these units behavior patterns (Tinbergen and Perdeck, 1950; Tinbergen, 2020), stereotyped actions (e.g., Berman et al., 2014), sub-second modules (e.g., Wiltschko et al., 2015), “expert defined categories” (Anderson and Perona, 2014; Egnor and Branson, 2016), “postures” (Schwarz et al., 2015), or “discrete behavioral events” (Golani, 1973; my own youthful folly). Only then do they attempt to relate these units to each other using, e.g., motivational, statistical, or cluster analysis models. This procedure sidesteps the basic zoological procedure of relating, measuring, and comparing identically connected (homological; see below) and organismic (body plan) relevant structures. Assuming initial, seemingly unbiased lack of knowledge, they end up with a list of fragmented, hardly-related and non-generalizable categories that could not be assembled into a unified architectural plan nor could they be compared, except for relative frequencies, across treatments, or taxa.

The current essay singles out two geometric properties - linearity and modularity - and highlights their manifestation across the genetic, anatomical, and behavioral levels, built on studies in evo-devo and animal movement-based behavior (Figure 1 Top). A linear topographical order of the Hox toolkit genes on the chromosome(s); a collinear order of their expression; serially connected anatomical modular segments linearly ordered along the Antero-Posterior (AP) axis; their movement linearly recruited along the AP axis; separately and sequentially along each of six coordinative spatial modules in actualgenesis, ontogenesis, and partly also in phylogenesis, neurological recovery, adult intact and drug induced behavior, and agonistic interactional behavior.

The progressive growth and differentiation of movement within and across spatial modules enacts a buildup in the organism’s neurological freedom of operational space. An opposite narrowing of operational space is induced by stress and dopamine agonist drugs. The organism’s operational space, encompassing action, perception, attention and primordial cognition and emotion, mirrors the dimensionality of physical space, including the continuity within, and discontinuities across, physical dimensions, the constraints imposed by the lower-level anatomical body plan and the genetics supporting it, and the affordances offered by the animate environment.

This essay offers a new synthesis portraying the morphogenesis of an organism’s genes anatomy and behavior architectural plan (*bauplan*, Figure 1B, bottom) in a comparative generalizable frame. The Anglicized term “body plan” was preceded by the German term *bauplan* (plural, baupläne), used in biology and introduced by Woodger in 1945, which means ground plan, architectural plan, or structural plan (Woodger, 1945; Hall and Hall, 1999; Rieppel, 2006).

## 2 Collinearity, modularity, and homology in the genes-anatomy-and-behavior architectural plan of vertebrates and arthropods

The order of the Hox genes along the chromosome(s) is the same as their order of expression in the embryo. These toolkit genes generate along the head-to-tail axis of arthropods and vertebrates a linear modular partitioning of the body into a kinematic linkage of multiple, specialized, rigid segments (metameres). Using a movement notation description that mirrors the mechanical kinematic constraints on this linkage, we propose, above the vertebrate anatomical body plan, a collinear and modular *bauplan* of movement-based behavior (Figure 1 bottom), comprised of six relatively independent spatial dimensions, or modules. Movement within each module unfolds in moment-to-moment morphogenesis (actualgenesis), and in ontogeny, in the same linear, head-to-tail order: the modules emerge, grow and differentiate in a fixed order, building up the freedom of movement of the linkage. Whole body movement around the hindquarters precedes movement around the forequarters. The same order applies to forward progression across vertebrate phylogeny. In stressful situations and under the influence of drugs, the spatial modules are eliminated following a “last in first out” rule, while a support module folds in an opposite, tail- to-head linear order, restricting the freedom of movement of the linkage. The same modules unfold and fold in the same order in arthropods.

Because each locomotor dimension involves the management of specific perceptual inputs relating to a specific physical dimension, the *bauplan* also manifests a hierarchy of attention and of primordial cognition. The momentary kinematic freedom of movement exercised by the homologous linkage discloses the animal’s understanding of the situation, communicating to the organism itself and to its phyletic congeners the emotion indicated by the affordances it offers, thus exhibiting the organism’s life-world (*umwelt*). The performance of novel movement types enables the performance of still unforeseen, more advanced types, liberating the organism from being distracted by external and internal stimuli, enriching its life world by endowing it with an increasing freedom to act with precision or not to act, on the basis of internal context. The portrayed *bauplan* may serve as a road map in studies of the homeotic, neural, and aspects of the cognitive levels of the pyramid of life.

A central theme of this work is the growth of behavior from its inception, at any stage of life: birth, exit from a den, courtship, hunting, and other instances that recur in natural behavior following a structured, well-defined choreography. The synthesis of the built-in rules, according to which many behaviors form during growth processes, has not been properly recognized. While pointed out in the ethological literature, it has not yet achieved recognition as a central theme of ethology. Possibly the most enigmatic example, yet outstandingly flamboyant, is the “war dance” of stoats (*Mustela ermine*) and ferrets (*Mustela euro*), involving stoting, logrolling in the air, zigzags, spins, loops, and very fast bounces. It is discussed in detail in the Discussion section. The geometric analysis of the rules of this buildup and subsequent shutdown across species, placing it in physical space, relating it to genetics, anatomy, and neurochemical modulation, within a biologically relevant comparative framework, and focusing on its growth and form is a central theme of this essay.

*The first step of defining the primitives of movement-based behavior in terms of their topological relatedness to the whole is critical*. The pyramid of hierarchical control of living matter is distinguished by an ascending order of levels, each level featuring its respective simplest element or primitive: atoms at the physical level, molecules at the biochemical, genes at the genetic, …, cells at the cellular, …, rigid segments at the anatomical, organs at the skeletal, etc. Remarkably, however, there is as yet no scientific consensus over what are the primitives of the next-level-up in the hierarchy, that of movement and of locomotor behavior. At the same time, while the anatomical body plan features highly in evo-devo, there are as yet no portrayed *baupläne* that encompass the genes, anatomy and behavior levels within the same frame for any phylum.

The aim of this essay is to identify both the primitives of movement, as well as the bauplan of the morphogenesis of movement-based behavior in vertebrates and arthropods. As illustrated below, for comparative anatomists it has been imperative to attend simultaneously to the primitives and the plan. Borges’ account of “Funes the memorious,” as related by a linguistic anthropologist (Chrisomalis, 2010) in an essay on the effect of written numeral systems on human cognition, illustrating the problem and the endeavor lying ahead of us:

“a person blessed or cursed with an apparently limitless memory… who told the author… that in 1886 he had invented an original system of numbering and that in a very few days he had gone beyond the 24,000 mark. He had not written it down, since anything he thought of once would never be lost to him. His first stimulus was, I think, his discomfort at the fact that the famous 33 gauchos of Uruguayan history should require two signs and two words, in place of a single word and a single sign. He then applied this absurd principle to the other numbers. In place of 7,013, he would say (for example) Maximo Perez; in place of 7014, The Railroad; other numbers were Luis Melian Lafinur, Olimar, sulphur, the reins, the whale, the gas, the caldron, Napoleon, Agustin de Vedia. In place of 500, he would say nine. Each word had a particular sign, a kind of mark; the last in the series were very complicated. I tried to explain to him that this rhapsody of incoherent terms was precisely the opposite of a system of numbers. I told him that saying 365 meant saying three hundreds, six tens, five ones, an analysis which is not found in the “numbers” The Negro Timoteo or meat blanket. Funes did not understand me or refused to understand me” (Borges, 1962).

Chrisomalis wryly points out that “whether we say 7,013 or Maximo Perez is not simply a stylistic choice” (Chrisomalis, 2010). Indeed, the invention of numeral systems (numeral literacy) opened up for human cognition an immense arithmetic manifold, revealing structure and arrangement such as odd and even numbers, prime numbers, zero, ordinal numbers denoting order, and cardinal numbers denoting quantity, distance and proximity between numerals, numbers “wrapping around” upon reaching a certain value – the modulus, and the arithmetic operations, including comparisons – all nonexistent in Funes’s epistemological particularistic world.

von Uexkull (von Uexküll, 1957) and later Gibson (2014) stated that organisms first project an operational meaning (affordance) on a perception, and then act on it in a way corresponding to that affordance. This dictum necessarily also applies to Funes and to literate arithmeticians: for Funes the one and only affordance of “Maximo Perez” and “The Railroad” is counting and then comparing the respective frequencies of occurrence of these “numbers” (and hence their conditional probability of appearance) in a sequence of similarly labeled numbers. In contrast, for a literate arithmetician, the affordances of the respective numeral counterparts of these numbers – 70/4 – include ordering the two numerals, comparing their sizes, calculating their distance from each other, as well as performing these and all the other arithmetic operations with all the other numerals across the whole arithmetic manifold. This, in a nutshell, is the difference between the use of disconnected (Funes type) building blocks, and the use of primitives which are *a priori* designed to be defined by their connectedness. This is also the difference between the building blocks listed in computationally derived ethograms that initially ignore extrinsic connections, and the primitives of movement expected in an “anatomical” paradigm of behavior. Funes and the computational phenotypers are either blind or disinterested in the affordances offered by the connectedness of the manifold generated by the respective primitives comprising it, be they numerals in arithmetic or movement primitives in behavior. But since there are no such thing as self-sustained facts floating in non-entity, because every distinction implies a universe of discourse (Whitehead, 2010a; Whitehead, 2010b), the primitives listed by Funes as well as by most current computational studies of behavioral phenotyping start by assuming a lack of knowledge about the connectedness of behavior, ending up with a fragmented particularistic landscape. In contrast, the paradigm employed in comparative anatomy and by the movement studies presented below defines and then isolates well-connected (i.e homologous) primitives, yielding a well-connected coherent manifold (i.e., a *bauplan*).

*Notations representing holistic features and connectedness highlight natural primitives and baupläne*. Numeral notation has been critical for exposing the structural manifold of arithmetics because of the connected nature of numerals and their indication of the infinite whole. Chemical notation has become practically indistinguishable from the theory of chemical bonding (Bawden, 2001; Brock, 2012) because the notation represents chemical connectedness of atoms in terms of (covalent and ionic) bonds, referring to the periodic table of elements, a tabular display of the chemical elements, which are arranged by atomic number, electron configuration, and recurring chemical properties. The periodic table, together with chemical notation, provides a *bona fide* example of a holistic, connected representation of an architectural plan, in this case of chemical primitives (atomic elements) and modules (molecules). In music notation notes relate to octaves and scales, thus manifesting a fixed relative position, and providing the foundation for music analysis and composition (Nash, 2015). In the same way, Eshkol-Wachman Movement Notation (EWMN, Eshkol and Wachman, 1958; Eshkol and Harries, 2001, 2004) features primitives consisting of single movements, each performed by a single rigid segment, characterized by its relative position along the skeletal linkage, and always defined in reference to its kinematic effect on that whole linkage. This connectedness endows the movement primitive with its homological identity. The notation’s spherical System of Reference (SoR) portrays not only already occupied spatial dimensions, but also the potential for evolutionary behavioral innovation. Another asset of this movement notation is the distinction it offers between continuous vs. discontinuous slabs of movement. These features are discussed in detail in later sections.

*Behavior is an extension of anatomy*. Konrad Lorenz, a founder of the discipline of ethology, accepted his share in the 1973 Nobel Prize in Physiology and Medicine with the reflection that his and ethology’s “most important contribution to science” has been the discovery “that the very same methods of comparison, the same concepts of analogy and homology, are as applicable to characteristics of behavior as they are to those of morphology” (Lorenz, 1974). In particular, to the same extent that anatomists use bones like *humerus* and *radius*, and skeletal organs like *head* or *right forelimb*, to establish the concept of skeletal homology, ethologists have aspired to isolate the particulate processes of behavior in order to establish behavioral homologs. Half a century later, however, Lorenz is mostly remembered for his extensive documentation of the “fixed action pattern,” which he presumed to be the elementary building block of behavior and a waypoint, not the goal, of the quest for behavioral homologies. The fixed action patterns (Barlow, 1996; Lorenz, 2012) and their contemporary descendants are as idiosyncratic as the terms used by “Funes the memorious,” disallowing geometric scaling and/or comparisons of behavior.

A substantiation of the Lorenz insight that behavior is an extension of anatomy, requiring the same methods of analysis, thus sends us to comparative anatomy in its current manifestations: developmental anatomy and developmental evolutionary biology (evo-devo). Unlike Borges’ hero, we promote generalizability by portraying here the natural geometric manifold that unfolds (and folds) in this process, revealing a universal architectural plan that encompasses genes, anatomy, and behavior, including perception, attention, cognition, and emotion.

**Figure 2:**
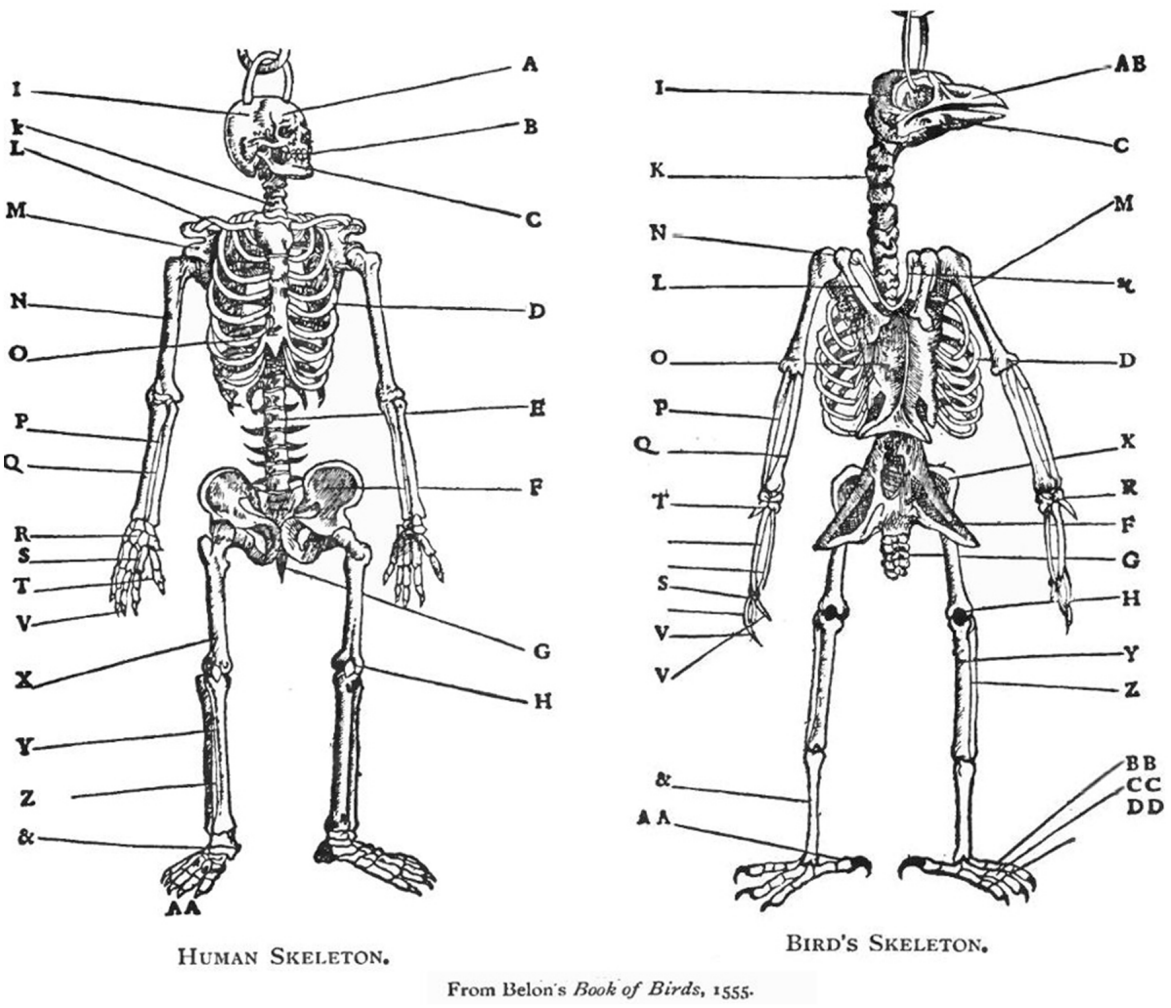
A human and a chicken skeleton share the same architectural plan (same body plan) (Belon, 1555). The whole skeletons, the respective organs (for example, the respective heads) as well as the individual bones (for example, the respective humeri) are homologous. According to the pre-Darwinian definition, the respective organs and bones are homologous because of their identical anatomical connectedness to other bones: they occupy the same relative position in the respective skeletons. According to the Darwinian definition they are homologous because they have descended from common ancestral bones and organs. Neither definition is based on the form or function of bones and organs. In the current essay we describe the homologous primitives (“bones”), modules (“organs”), and *bauplan* of behavior (Belon, 1555).

*The topological definition of anatomical homology*. A straightforward example of the ontology that was used in comparative anatomy for several centuries is presented in a French anatomy book published in 1555 (Figure 2). The concept of homology is the cornerstone of comparative anatomy. Comparing the presumed right upper arms of two animals belonging to two different *tetrapoda* taxa, the one and only way to ascertain that one is indeed measuring the respective right upper arms, is by making sure that both segments are articulated on their respective proximal ends to the rest of the linkage through spherical joints (shoulders), and on their respective distal ends through hinge joints (elbows), which are in turn connected to two parallel bones (radius and ulna), which are in turn articulated on their respective distal ends through synovial joints (radiocarpal joints)…etc. Defining the two bones as upper right arms or humeri establishes them as identical (homologous), tacitly implying a (homologous) body plan. There can be no rigorous science of comparative anatomy without ascertaining that a humerus is indeed a humerus, whether it is embedded in the arm of a human, the wing of a bird, or the foreleg of a horse, regardless of its respective form and function in these different taxa. Its identity is secured by Saint Hilaire’s (Saint-Hilaire, 1822) *principle of connections*, stating that “the sole general principle one can apply is given by the position, the relations, and the dependencies of the parts, that is to say, by what I name and include under the term connections (in Russell, 1945; see also Beer, 1974).” Bones that occupy the same relative position in the respective skeletons of different taxa (and genes that occupy the same relative position in the gene sequence of different taxa), are defined as homologous.

This topological definition of homology preceded and served as the basis for the later, Darwinian, definition of homology, which was based on common descent (Bateson, 1894). Needless to say, identity (homology) is currently secured by Darwin’s historical principle of common descent (Darwin, 1909). With behavior, however, historical evidence is absent. Therefore, the earlier, topological definition can still be useful if an invariant relative position of a behavioral primitive or a behavioral module can be demonstrated (Golani, 2012). The human and avian humeri are two manifestations of the same character identity (hence their sharing the same name), manifesting two distinct *character states* (Wagner, 2000), hence the differences between the humerus of a human and of a bird. Most important, the morphogenetic (developmental) history of the *humeri* is part and parcel of their definition (Hall, 1995). The use of the principle of connections in the definition of behavioral homologies is at the heart of the argument presented in this essay (for the primacy of a structural definition of homology; Wagner, 1994); for other definitions of homology see (Donoghue, 1992; Haszprunar, 1992).

*Ethograms versus movement primitives*. Having defined the presumed building blocks of movement on the basis of so-called expert decisions, or intrinsic features of the movements, or intrinsic features of the movement video-images, or the specific demands of a study, or unsupervised deep learning procedures (which not unlike Funes, rely on the cognizing or computational agent’s impeccable memory), provides a list of presumed building blocks, along with the probabilities of transitions between them. Take for example the list of “subsecond blocks of behavior” isolated in mouse open field behavior by an unsupervised deep learning procedure (Wiltschko et al., 2015). The automated procedure isolates body states from a 3D video, manifested as repeated pixel formations, and then counts their frequencies and reports the location and timing of their performance. The next stage includes manual labeling of the states. The labeling is independent of measurement and is based on subjective “expert evaluation,” not unlike classical ethologists observing behaviors and labeling them deliberately. For example, the deep learning program identified two states that were subsequently labelled “low rearing” and “high rearing.” However, this scaling of rearing episodes was subjective rather than data driven. Thus, even low and high rearing cannot be compared on any measurable scale, except for frequencies of performance, even in the same animal and in the same session, let alone in a comparison of “rearing” states across taxa.

**Figure 3:**
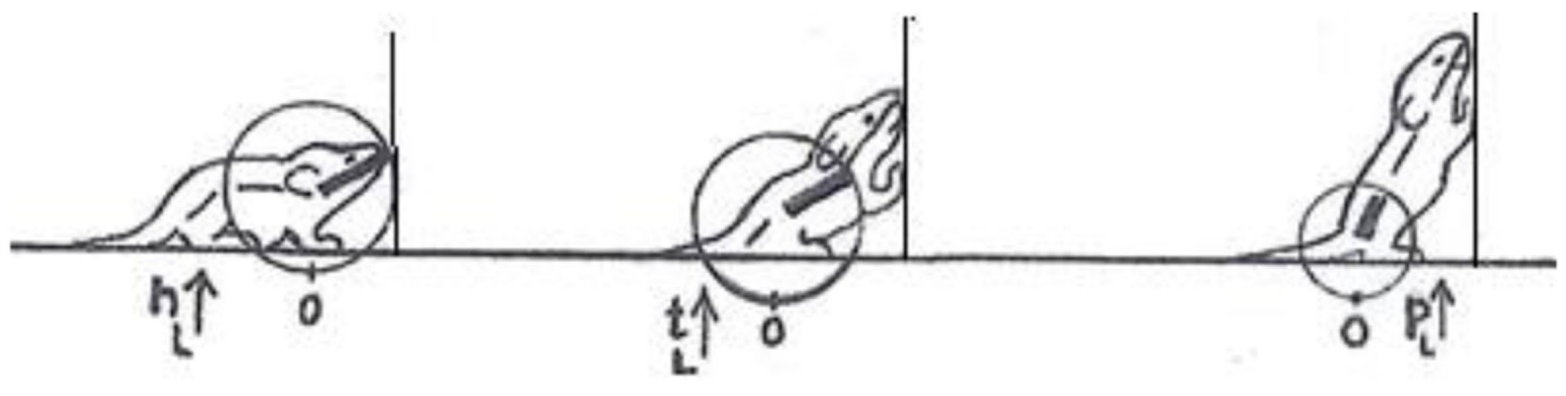
An illustration of three primitives of movement in an infant rat (*Rattus norvegicus*): head vertical movement (left), torso vertical movement (middle), and pelvis vertical movement (right). The straight lines represent the parts of the trunk. The circles stand for the spherical coordinate systems, respectively, centered at the root of the movement. The heavy lines stand for the respective part of the trunk that moved. During head movement (left) only the head changed its angular relation to its next caudal part; during torso movement (middle) only the torso changed its angular relation to its next caudal part, carrying along the neck and the head, and during pelvis movement (right) only the pelvis moved carrying along the torso, the neck and the head. Each of the notational expressions written under the illustrated movements stands for a primitive movement-type. The 3 illustrated movements constitute the beginning of the fourth natural module illustrated in Figure 1 as the “vertical on hind legs with snout contact,” which amounts to tumbling pelvis-on - a backwards whole-body rotation around the side-to-side axis of the body (Modified from Eilam and Golani, 1988).

In contrast, a description of the orientation of the parts of the trunk in reference to individual spherical coordinate systems schematically centered on their respective caudal joints would sensitize and prompt the observer to perceive and record the behaviors illustrated in Figure 3 as head raising (left), torso raising (middle) and pelvis raising (right) as three increasingly larger amplitude movements, along the same, vertical, dimension, recruiting an increasingly larger number of trunk segments, in an antero-posterior (AP) order: first the head, then the torso, which also carries along the head, and then the pelvis, which also carries along the torso and the head. In contrast to the fragmented list yielded by an ethogram, the kinematic primitives yield a fully connected description disclosing the kinematics of a linkage as well as the manifestation of a behavioral spatial module. The performance of these movements in sequence may constitute a morphogenetic buildup process, and the geometrical nature of this type of description allows a comparison across taxonomic groups. Unlike the building blocks of ethograms, the primitives illustrated in Figure 3 (i) refer to the whole kinematic linkage (much like the specific bones in a skeletal body plan), and (ii) can be assembled into higher level modules (here a vertical spatial module), which may amount to a whole body rotation around the side-to-side axis of the body of 360° amplitude (Figure 1, vertical on hind legs with snout contact), which can in turn be embedded within a whole body *bauplan*.

Segmentation of behavior based solely on deep learning cannot yield a coherent model of the organization of behavior. Using it without impregnating the presumed building blocks with extrinsic connectedness illustrates “the fallacy of the first step,” which says that “climbing a tree is not the first step to the moon” (Bar Hillel in Dreyfus, 2012). In contrast, a literate kinematic description has the potential of demonstrating the connectedness and generalizability of movement-based behavior. Since the obtained description is morphogenetic, it furthermore, specifies generative rules rather than conditional probabilities.

To flesh out Lorenz’s insight, the movement primitives of vertebrates and arthropods should yield a *bauplan*: an overall natural geometrical manifold of behavior consisting in natural kinds (Wagner, 1996; Stanford Encyclopedia of Philosophy, 2022; Bird, 2018) in the same way that in comparative anatomy bones form organs, which form a body plan. The homology of the primitives we seek to define (single movements of single rigid segments) is established by (i) demonstrating their connectedness (Saint-Hilaire, 1822) to other geometrically-defined single movements and by (ii) embodying in their definition the indivisibility of the growing and differentiating organism.

To sum up, the major fallacy shared by Lorenz and by ethogram constructors of all times, including the current computational phenotypers of behavior, is their failure to even recognize the need for primitives that derive their identity from their relative position in the *bauplan*, a practice which is taken for granted and implemented *en passant* by anatomists. The very labeling of a bone as a humerus serially implies the whole body plan. In this way each and every skeletal primitive becomes a topologically invariant, and therefore generalizable and comparable unit. In the same way, whereas “scanning” is a particularistic mnemonic label for a behavior, a “positive vertical head movement “is a topologically invariant primitive because its first performance following extended immobility always precedes the first positive vertical chest movement, and always follows the first forward trunk movement. Because of its fixed relative position in the sequence it is comparable-across-phyla.

The use of ethogram-type, essentially disconnected, behavioral building blocks and categories, whether defined on the basis of their intrinsic properties, or *ad hoc*, has been useful in fields such as behavior genetics, behavioral pharmacology, and neuroethology. In these fields, the subject of interest is not the organization or structure or comparative study of behavior, but rather the study of the lower levels of the pyramid of life, such as the biochemical, physiological, genetic, and neural levels, whose connectedness, continuity, structure, etc., are the focus of interest. It is the connectedness and continuity in these underlying levels, which are both supplied and enriched by the quantification of behavioral markers and measures. In these disciplines, even crudely and intuitively defined behaviors can often be useful as a springboard for studying the levels that mediate behavior. Studies in these disciplines, however, largely do not contribute to the comparative study of the structure, organization, or connectedness of behavior itself. While quantification of homologous behavioral primitives is not an absolute necessity in these fields, phenotyping in terms of homologous natural kinds (Wagner, 1996; Stanford Encyclopedia of Philosophy, 2022; Bird, 2018) would be more likely to improve measurement (for the current feasibility of such endeavor, based on high throughput tracking, see section on “*Key managed kinematic quantities and the organism’s umwelt*”).

*Anatomical modules.* In comparative anatomy the bone primitives whose definition includes their morphogenetic history (Wagner, 1989; Hall, 1995) are embedded within organs, which are the modular ontological components of comparative anatomy. For example, a humerus, a radius, an ulna, metacarpals, and phalanges are the constituents of a forelimb. The definition of this module also encompasses its entire morphogenetic history, as a discrete, quasi-independent, locally integrated process and individualized yet interconnected part, or developmental field (Opitz, 1985; Shubin and Hall, 1994; Raff and Valentine, 1997). The right forelimb module, the head, the trunk, or the right hind leg, are not just products of a reasonable division of the organism’s body into parts; but, rather, biologically-real entities of a profound morphogenetic unity. As with the primitives so too with the modules: the embryological development of complex structures is determined and controlled in a spatially coordinated, temporally synchronous, and segmented hierarchical manner that on the one hand implies the whole organism, and is defined by its relative position (connections), and on the other hand expresses both non-specific (that is, phylogenetic, manifesting character identity) and species-specific genetically-coded developmental information (manifesting character states). To cite an example of this type of organization manifested in common anomalies: “identical anomalies produced by such different causes as the presence of an abnormal number of chromosomes in a cell, gene mutation, teratogenic chemicals, and certain surgical procedures show that embryonic primordia respond as units in the production of anomalies of anatomical structure. Hence, they must also act as units during normal ontogeny. The presence of identical malformations in different mammalian species identifies developmental and anatomical homology. These abnormal and normal morphogenetic reactive units are the equivalents of the classical experimental embryologist’s regeneration fields, which are those units of the embryo in which the development of complex structures appropriate to the species is determined and controlled in a spatially coordinated, temporally synchronous, and involving a conservation of form in a hierarchical manner that expresses both phylogenetic and species- specific genetically coded developmental information” (Opitz, 1985).

*What is to be expected of movement modules*. To flesh out Lorenz’s insight further we show below that, in the same way that bone primitives are organized into organ modules, distinct chords of single movement primitives can be organized in kinematic modules. These modules have a distinct morphogenetic history, and are performed in a relatively discrete, quasi-independent, locally-integrated process along distinct spatial dimensions. For example, horizontal movements that start at the head, and grow in amplitude to progressively recruit all the parts of the trunk in whole-body horizontal movement (whole-body pivoting around the hind legs; Figure 1 bottom, top line) behave as a distinct physiological subsystem. But “horizontal” tacitly implies “vertical,” even as a future morphogenetic option or absence thereof, and implies a dimensional operational space, even as an operational option. Anatomical modules (organs) are relatively few in kind and number, with a lot of empty phenotypic space between them (Alberch, 1989), e.g., there is no organ that consists in a combination of a head and a forelimb. If indeed behavior is an extension of anatomy, we would expect that, at least during the early stages of behavioral morphogenesis, the locomotor modules will also be distinct, their number relatively small, and they will be added to the organism’s movement repertoire separately and sequentially, implying a separate “bookkeeping,” module by module.

*Body plans.* The body plan concept has formed the backbone upon which much of the developmental and evo-devo current research is anchored (Willmore, 2012). Remarkably, the 35 body plans characterizing the currently extant 35 phyla of multicellular organisms all appeared about 550 million years ago, when fossils first appeared (Conway Morris, 2006) during an apparent, relatively sudden “explosion” (Raff, 1996; Marshall, 2006). No new body plans have emerged since (Fitch and Sudhaus, 2002), nor have any combinations between body plans been formed, suggesting that each of these 35 architectures manifests a solution whose intact entirety is critical for life (Riedl, 1978; Wagner and Laubichler, 2004). Multicellular organisms are composed of multiple modules, with each module characterized by its distinct morphogenetic history and its final shape. All the modules, together with their characteristic invariant connectedness, form the organism’s body plan (Woodger, 1945; Eldredge, 1989; Raff and Valentine, 1997).

The central role played by the body plan concept is exemplified in the development of the serially repeated set of vertebral structures and somite in vertebrates (Raff, 1996), or in the development of the limbs (Shubin et al., 1997; Jeffery et al., 2018) or in the morphogenesis of rhombomeres in the transiently dividing segments of the developing neural tube within the hindbrain region (Krumlauf, 2016). In both arthropods and vertebrates the trunk is partitioned into specialized groupings of modules like the cephalon, thorax, and pygidium (in trilobites); the head, thorax, and abdomen (in insects); or the head, neck, upper and lower torso, and pelvis (in Tetrapoda). Laterally disposed, equivalent body parts (antimeres), are articulated on both sides of the anteroposterior (AP) axis, like fore- and hind limbs in vertebrates and paired appendages in arthropods, themselves segmented along the Proximo-Distal dimension (Wagner, 1996; Raff and Valentine, 1997). The body plan specifies the identity and the topology of the phenotype, without which the molecular mechanisms that fix, mediate, and support these features could not have been discovered, in the same way that the genetic basis of any complex phenotypic trait could not have been established without the awareness of this trait.

Since the main goal of the present essay is to also encompass behavior within the same plan, and the term body plan refers only to anatomy, we adhere here to the original term *bauplan* when also referring to behavior. If indeed behavior is an extension of anatomy, then each body plan should enact a corresponding behavioral *bauplan*. As with anatomy, morphogenesis must be the key for deciphering the organization of this *bauplan*, in the same manner that it is the key for deciphering the organization of an organism’s anatomical body plan. Since morphogenesis involves differentiation, the more general anatomical taxonomic characters and anatomical structures should appear in development before the more specific and the more specialized ones, as was indeed established for anatomy two centuries ago (Von Baer, 1828). Hence the primacy of a definition of body plans on the basis of their embryological history. The same principle should apply to behavioral *baupläne*.

As with anatomy, the *bauplan* of behavior is not only of significance in and of itself, but is also indispensable as a roadmap for exposing the molecular mechanisms that mediate it.

*What is to be expected of movement baupläne*? Having defined bones as primitives, organs as modules, and the invariant topological connectedness of modules across development as an anatomical body plan, we may join a founder of the study of the embryology of behavior, George Ellet Coghill (Coghill, 1929) in his rhetorical question: “what is (skeletal) anatomy for?” with the obvious answer being “(skeletal) Anatomy is for behavior” (Herrick, 1941). Coghill noted in 1929 that “it is hard to understand why the same method which has proved so suggestive in comparative anatomy has not been pursued more vigorously by embryologists.” But whereas Coghill focused on behavior as an extension of neural anatomy, we focus on behavior as an extension of skeletal anatomy. Behavior, in the context of a kinematic linkage of rigid segments has a geometrical structure. Thus our subject matter is that of the natural geometry of behavior, a geometry that can in turn serve as an explanation for the supporting anatomy. The rest of this essay is dedicated to geometrical aspects of the genes, anatomy, and locomotor behavior *bauplan*. Locomotor behavior relates to the spatio-temporal forms “carved” by the articulated body in space and time.

*The dimensionality of a bauplan.* Back in 1918, the (mathematician and) custodian of biological form (d'Arcy, 1942) wrote a letter to the (mathematician and) custodian of “process philosophy” (Emmet, 1966):

“My dear Whitehead, I have been thinking, or dreaming, lately over a matter of which I know so little… the first difficulty seems to me, to decide whether there are, in reality, three dimensions of space; and the second question is, whether there be, in reality, three dimensions or no, how did we come to think, or to find out, that it is so.

I suggest, that we are … fundamentally guided, by the influence of Gravity…we are always face to face with a vertical axis, and with a plane (or apparently plane) surface perpendicular to it. In other words, the right angle assumes a very special importance, and, consciously or unconsciously, we refer everything in space to trihedral coordinates. Now suppose… that we were of so minute a size… that gravity would have no sensible hold upon us; and suppose, owing to our minute size, that we were mainly under the influence of other, say molecular, forces. Then, to begin with, we should know nothing about a vertical, and care nothing about a right angle. And suppose, in the next place, that we lived in some sort of “close-packed” or crystalline medium, say a tetrahedral one, we should never dream of three-dimensional space …So, paradox or no paradox, I seem to be driven to the conclusion that there is a quibble, or even a fallacy, underlying our definition of Space, or of Dimensions, (or perhaps both). Perhaps that dimensions are not necessarily rectangular: or that perpendicularity, inter se, is not a fair condition to postulate of them. That the Space which actually exists is quite independent of dimensions… does that in any way prove that we have a right to say there are, in reality, three dimensions; is it anything more than a mathematical figment, an elegant formula…. given a symmetrical individual in symmetrical space, how on earth could you ever teach him what right and left meant. He would obviously have no right and left and space itself has, obviously, no right and left. And so, I come back to my query. Has Space really three dimensions; or is this only a convenient figment of terrestrial, and large and clumsy, mathematicians?

Ever yours faithfully,

D’arcy Wentworth Thompson.”

Thompson, whose concern is the growth of biological form, appears to be consulting Whitehead (2010a, 2010b) about the ontological status of spatial dimensions: Does physical three-dimensional space exist in and of itself, regardless of an organism’s biological context? The current essay shows how, while the animal’s three-dimensional life-space is enacted dimension by dimension in morphogenesis, in reference to the physical vertical absolute (gravity), the life-space is embedded within physical space, enfolding its dimensionality, and thus disclosing the animal’s (cognitive) understanding of it.

To provide a context for the geometry of the animal’s operational world and the behavioral *bauplan* enacting it, we first portray below the geometry underlying the main morphogenetic processes leading to the construction of the animal’s anatomical body plan: collinearity between the toolkit (Hox) genes’ topographical alignment, Hox order of expression along the body, morphogen shaping of the AP axis, cell movement along the AP axis, and movement-generated axial forces exerted by the newly-formed muscles shaping the skeleton.

*The Hox genes are aligned in a fixed order on the chromosome*. Our geometrical journey starts with a small number of “master” genes assembled in one or several clusters on the chromosomfvs of arthropods and chordates, aligned in a fixed order on the chromosome (Gaunt, 2018). The Hox are transcription factor genes: Hox proteins encode and specify the characteristics of “position,” ensuring that the correct structures form in the correct places in the body. The expression of a Hox gene in a cell confers segmental or positional identity to that cell, but does not form the actual segment itself.

*Collinearity between Hox genes’ physical alignment on the chromosome and their order of expression.* In the second station of our geometrical journey, the Hox genes are expressed in the embryo’s body, along the AP axis, in partially overlapping domains, and in the same linear order of their alignment on the chromosome(s). The developmental characteristics of each zone reflect the combination of Hox genes that are activated or repressed in its cells. The geometrical correspondence between the physical alignment of the genes and their order of expression has been termed collinearity (Lewis, 1978, 1985; Lewis et al., 1980). Because *Hox* gene activation is concurrent with axial extension and limb growth, the different areas of the body and limbs express distinct combinations of *Hox* gene expression. During the early stages, Hox expressed and other morphogen concentration gradients span the entire length of the embryo. Their relative amounts signal, at precise AP and DV positions defined by their longitude, latitude and altitude coordinates, the topographical location of limb buds on the embryo’s envelope (Carroll, 1995). Which Hox gene turns on, where does it turn on, and when does it turn on is foundational in evo-devo (Goodman and Coughlin, 2000; Papageorgiou, 2021).

Morphogen and cell movement both shape and are shaped by the AP and DV axes. In the third station of our journey, dividing cells exhibit targeted movement to specific sites to form tissues and organs (Solnica-Krezel, 2005; Ananthakrishnan and Ehrlicher, 2007). Morphogen and cell movement both determine and are determined by the AP and DV axes. Morphogen gradients guide cell movement. In mice for example, cell movement specifies the AP body axis (Migeotte et al., 2010). Directed movement and other rearrangements that shape organs and body axes involve a fine-grained control of cell polarity. One type of cell polarity is established in reference to the axes of the embryo or organs (Veeman and McDonald, 2016). Vertebrate gastrulation involves four evolutionarily conserved morphogenetic movements governed by signaling pathways. Two of them, convergence and extension, are associated with axis elongation in both vertebrate and invertebrate embryos (Supplementary Video 1). The process, termed convergent extension, involves the lengthening and narrowing brought about by the collective heaping up of cells toward the embryonic axis (Wallingford et al., 2002). Cell movement related to the main axes is a substantial component of overall cell movement during certain stages of embryogenesis (Solnica-Krezel, 2005).

*The Hox genes implement the principle of connections along the AP axis.* In the fourth station of our journey we find the linearly arranged modular end products of the morphogenetic process. In vertebrates, the embryonic AP axis is segmented into a fixed species-specific number of somites which varies across species, ranging from as few as 32 in zebrafish to 35 in humans, and to more than 300 in some snakes.

*The modular developmental genetics of morphological homologs*. Pondering about the apparently loose relationship between morphological characters and their genetic basis (Hall, 1995; Butler and Saidel, 2000; Wilkins, 2002), Wagner (2007) proposes that it is the historical continuity of *gene regulatory networks* rather than the expression of individual homologous genes that underlies the homology of morphological characters. These modular networks, referred to by Wagner as “character identity networks,” enable the morphogenesis of morphological homologs.

*The ancientness of the Hox genes and of extant body plans*. The discoveries of the Hox genes’ structure and function are among the most important discoveries in biology in the last few decades. These genes and their respective signaling pathways are shared by most living phyla, and are implicated in sculpting the body plan. They have already functioned more than 500 million years ago, before the famous Cambrian Explosion that gave rise to the 35 extant animal phyla mentioned above. These genes, their order on the chromosome, and their ordered expression, were so crucial for survival that their anatomical and operational sequencing had been preserved throughout this enormous span of animal evolution (Carroll, 1995; Durston, 2012). If movement-based behavior is an extension of anatomy, its *bauplan* should also be ancient, and its morphogenesis too should be shaped by Hox genes.

*Collinearity between AP formation of modular organs and their order of recruitment in movement.* Cell movement in reference to the main axes culminates in the linear AP formation of modular organs along the whole skeletal body plan (which also constitutes a module). Remarkably, these modular organs begin to be recruited in vertebrates in movement in the same linear order, separately along each of the six spatial modular dimensions. But in both the vertebrate embryo and neonate, during the transition from organogenesis to movement, muscle-induced mechanical load is involved, via chemical cues, in chondrocyte proliferation, shaping of individual bones, regulating the 3D organization of skeletal elements, and many other aspects of skeletal differentiation and growth (reviewed in Felsenthal and Zelzer, 2017).

*The AP midline axis is a morphogenetic field used as reference for the anatomical Body plan*. Evo-devo and developmental anatomy support the ontological status of the arthropod and vertebrate midline. This midline is not an imaginary abstract sagittal plane dividing the right from the left half of the body. It is a biologically real developmental *field* of profound morphogenetic importance: a part of an embryo that reacts as a spatiotemporally coordinated unit to normal localized forces of organization and differentiation.

**Figure 4:**
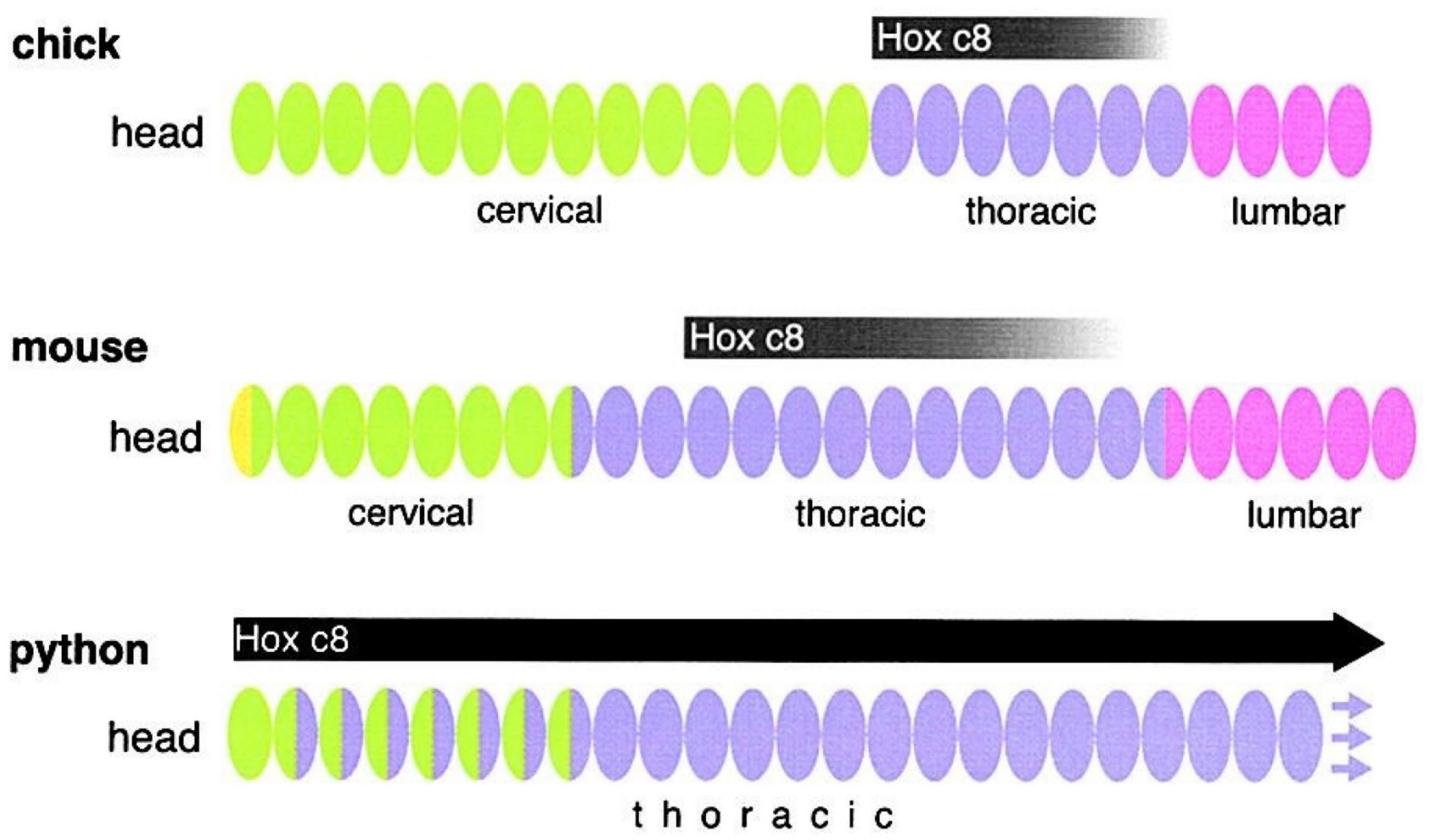
The Evolution of Hox Gene Regulation Correlates with the Evolution of Axial Diversity. Hox genes are expressed at different relative positions along the AP axis in the mouse, chick, and python (Belting et al., 1998; Cohn and Tickle, 1999). Figure Copyright 2000 by Cell Press; Endless Forms: The Evolution Minireview of Gene Regulation and Morphological Diversity (Carroll, 2005). Permissions and image licenses have been obtained from the copyright holders (Source: Elsevier and Copyright Clearance Center).

The morphogenetic field construct, finding its earliest expressions in the beginning of the previous century (Harrison, 1918; Weiss, 1926; Child, 1941) was enhanced by Opitz (1985) and expanded by evo-devo to the now established morphogenetic module (Wagner, 1996; Wagner et al., 2007). If behavior is an extension of anatomy, then it should necessarily be organized in reference to, and around this midline AP axis field, and reflect its linear geometry (Figure 4).

*An interim list of features of a technology for analysis and description of movement should yield:*

- A representation that would highlight growth and differentiation (as in anatomy).
- Primitives, modules and generative rules of movement that would be imbued with reference to skeletal connections, to organismic aspects (*bauplan*), and to the organism’s situatedness in the environment (e.g., base of support and the mechanical constraints it implies, orientation in reference to gravity and to the horizontal domain).
- Demarcation of continuity and discontinuity (as within-, versus across kinematic spatial dimensions).
- A way to extend the body plan features to features of movement (e. g., single segments to single segment movements).
- Circular movements of single segments centered on joints (single segments can perform only circular movements).
- Rules to determine which of the two joints of a segment should be used as the origin for each joint’s reference frame in every situation (the reference frame should be centered on the joint closer along the linkage to the base of support).
- Whole-body axes that define movement-space and impregnate it with features that mirror the indivisibility of the organism (e.g., whole-body AP axis).
- Primitives that are natural kinds (Wagner, 1996; Stanford Encyclopedia of Philosophy, 2022; Bird, 2018), and reflect an indivisible whole: Individual spherical coordinate systems for all segments, gyroscopically oriented in reference to a General Spherical System of Reference (SoR) representing absolute directions (in order to obtain a situated description);
- Primitives impregnated with coordinates that refer to the environment (relevant, embodied and embedded; as gravity-induced vertical, and absolute horizontal directions).
- A geometrical description, always in reference to support (so as to anchor behavior to the substrate and induce a mechanical hierarchy).
- A description in terms of movement, which tacitly implies perception and attention (a phenomenological description).

**Figure 5:**
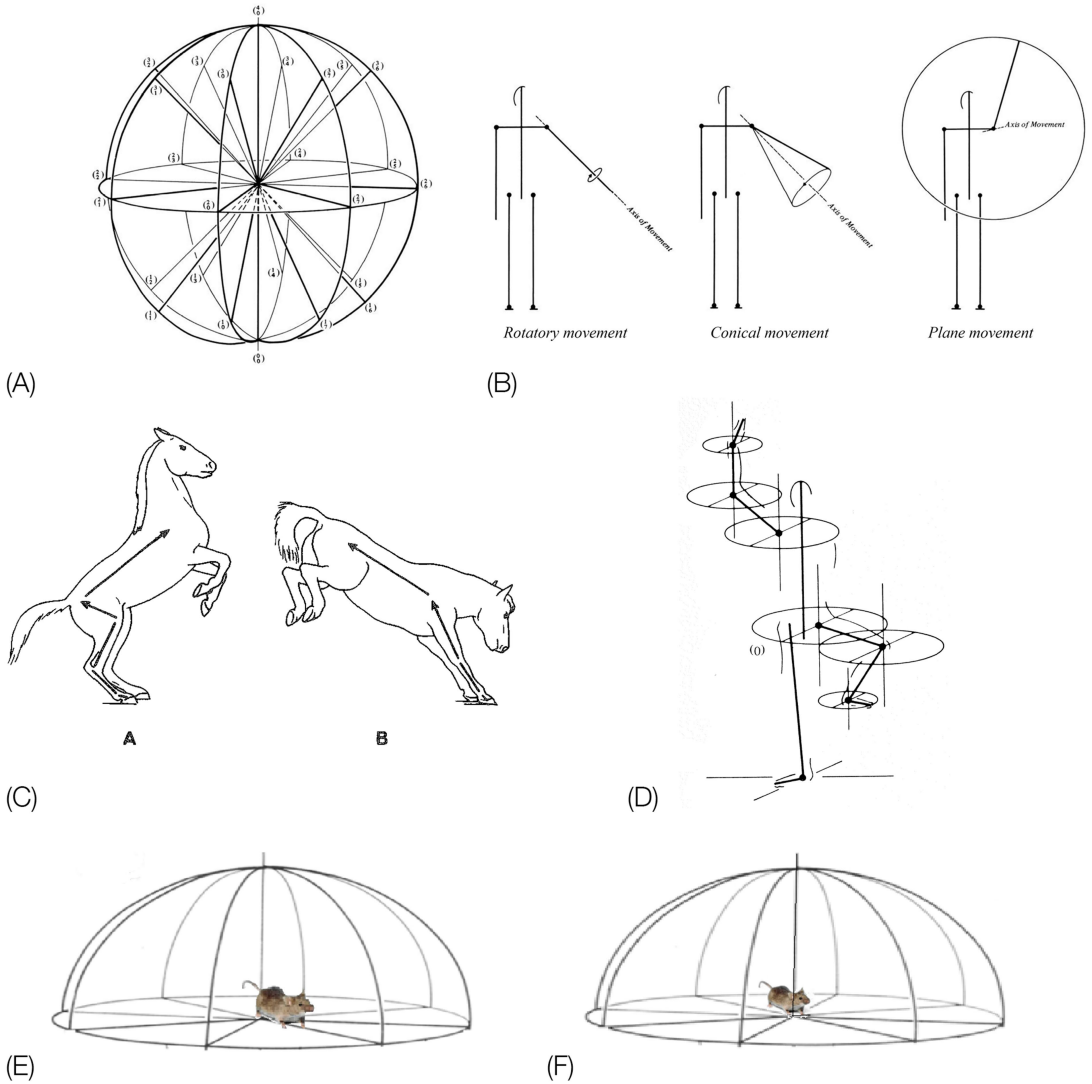
**(A)** EWMN General System of Reference (SoR). **(B)** The three types of EWMN movements: rotatory, conical and plane movements. **(C)**. The law of heavy and light limbs applied to the parts of the body of a horse during rearing on the hind legs **(C,A)**, and on the forelegs **(C,B)**. Movement of the feet in **(C,A)** changes the location and orientation of all the parts of the body connected to it toward and including the head. However, when its weight is shifted to the forelegs, movement of, e.g., the pelvis, in **(C,B)** changes only the location and orientation of the hind legs, having no effect on the parts connecting to it toward the head (from Golani, 1992a). **(D)** Individual SoR are centered on the heavy joint of the respective segments. Only the horizontal planes of the respective spheres are represented for clarity of exposition. The individual systems of reference are parallel at all times to the General SoR (After Eshkol and Harries, 2001). Copyright for **(A,B,D)**: The Noa Eshkol Foundation for Movement Notation. **(E)** The horizontal plane of the General SoR centered on the hind legs. When the mouse shifts its weight to its hind legs and pivots on them, movement of the pelvis changes the location and orientation of the whole trunk. **(F)** The horizontal plane of the General System of Reference centered on the forelegs. When the mouse shifts its weight to its forelegs, movement of its chest changes the location and orientation of its lower trunk, pelvis, and hind legs.

Eshkol-Wachman Movement Notation (EWMN, Eshkol and Wachman, 1958; Eshkol and Harries, 2001, 2004) is endowed with these features (Figure 5). To portray the geometry of the behavioral *bauplan* EWMN first define prone immobility as a reference posture (*zero position*). Then it characterizes the mechanical hierarchy between the parts of the trunk during movement (*heavy and light limbs hierarchy,* Figure 5C). Using zero position and the heavy and light limbs hierarchy, EWMN derives a description in *body-related* frame from a description in the *absolute* frame (the General SoR, Figures 5A,D). All this provides EWMN with an unequivocal definition of what is a *single movement* (Figure 5B) which is necessary for defining *the behavioral modules* and the *bauplan* of behavior they form (Figure 1). We next highlight the importance of each of these components.

**Figure 6:**
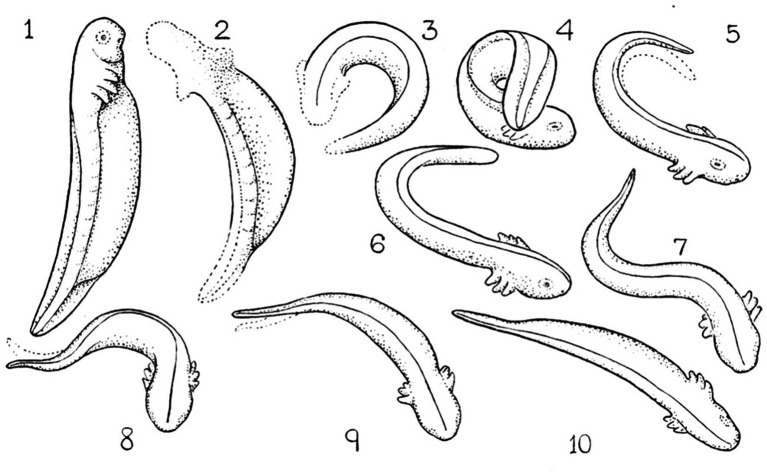
Locomotor embryology in Ambystoma. Starting from immobility (zero position, 1), unilateral horizontal movements increase in amplitude (2–6). When two horizontal (lateral) movements follow each other in opposite directions in proper phase difference (7–10) forward progression emerges (Coghill, 1929). Permissions and image licenses have been obtained from the copyright holders (Source: Springer Nature and Copyright Clearance Center).

*Zero position*. To highlight the topological isomorphism and continuity between the genetic, molecular, anatomical, and movement levels (Figure 1), we represent movement in reference to the midline AP axis (in a bilateral animal) by defining a prone posture, in which the organism’s axial modules are arranged in a linear order along a relatively straight line (Eshkol and Wachman, 1958; Eshkol and Harries, 2001, 2004). Maintaining immobility in this posture and proceeding from it with growth and differentiation of movement are exhibited in embryos and in stress-related situations: for example, in the embryos of salamanders (Coghill, 1929) (Figure 6) and fish (Tracy, 1926), following exposure to a novel environment (Golani et al., 1981; Eilam and Golani, 1988), the proximity of a rival (Golani and Moran, 1983), the immobilizing effect of dopamine agonists (Szechtman et al., 1985; Adani et al., 1991; Golani et al., 1997), and a severe bilateral lateral hypothalamic lesion disrupting dopamine release in the striatum and subsequent denervation super sensitivity to dopamine (Golani et al., 1979).

*The “heavy” and “light” limbs hierarchy.* Since the parts of the trunk are serially connected to each other, there is a mechanical dependence between them: a movement of a segment belonging to the linkage might influence the location and trajectory of other segments belonging to that linkage. This dependence is expressed in *EWMN* (Eshkol and Wachman, 1958) by the law of “heavy” and “light” limbs (Figure 5C): the closer a segment is to the point of support of the linkage, the “heavier” it is and the farther it is from the point of support, the “lighter.” The “heavier” the segment, the more segments’ locations and trajectories are influenced by its movement. A movement of the lightest segment has no effect on any other segment.

An individual spherical SoR is centered on the “heavy” joint of each segment (Figure 5D). The “light” end of such a segment always traces a circular path (a circle or a part of a circle). Since such a SoR is centered on the respective heavy joints of all the segments belonging to the linkage during its movement, these relations can be described also during movement of the whole linkage. The path traced by the free end of the linkage is the sum of the circular movements of the individual segments belonging to that linkage. The individual spherical systems of reference are parallel at all times to the spherical General SoR (Figure 5A), whose vertical axis coincides with the vertical physical absolute determined by gravity, and its horizontal directions are fixed in reference to the environment, securing (together with the continuous reference to the base of support) the situatedness of movement in the environment.

In zero position, lateral movement and absolute horizontal plane movement coincide. Similarly, Dorsi-ventral and absolute vertical plane movement coincide. However, once a heavy segment in the linkage moves out of zero position, it carries along away from the midline zero position all the serially connected, anterior, lighter segments. This disallows the observer to relate the movement to the absolute frame. In such case, the absolute frame is conceived as being carried along on the moving heavy segment and movement of the light limb is described in reference to this carried along frame as though it is the absolute frame: the carried along frame “remembers” the absolute coordinates, and the relations of the light segments to the heavy segments are described as though the heavy segment did not move. Hence the description of, e.g., otter shrew (*Potamogale* sp.) swimming as body-related horizontal (rather than lateral) undulation, and a quadruped’s galloping as body-related vertical (rather than dorsi-ventral) undulation.

**Figure 7:**
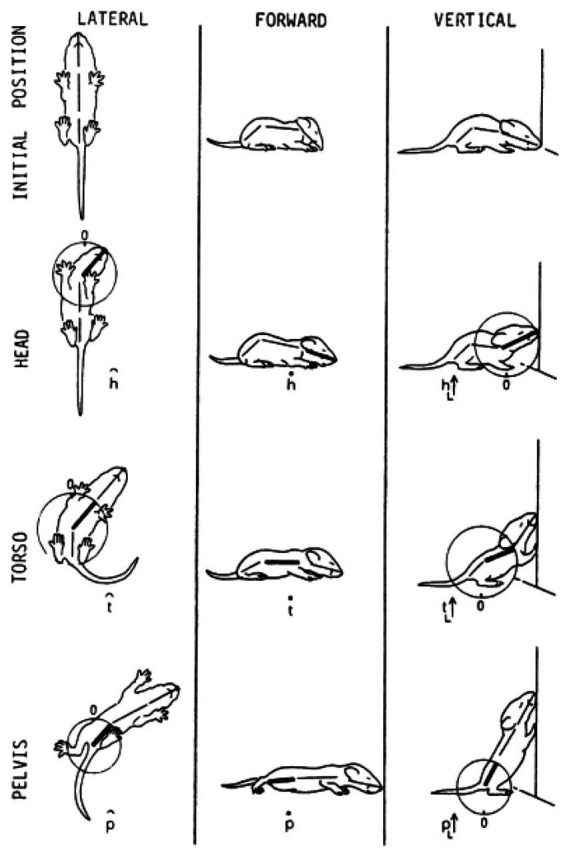
The build-up of the locomotor repertoire of an infant rat proceeds during “warm-up” linearly along the AP axis, separately along each of the first three modules. The trunk is schematically conceived as three rigid axes. The “heaviest” segment that moved, carrying along the “lighter” segments lying ahead of it, is marked by a heavy black line. Every part of the trunk has its own spherical coordinate system, but only the sphere of the heaviest part that moved is illustrated. Each drawing depicts a single movement (a primitive) represented by a single notational expression. Notational symbols designate the heaviest part that moved and the type of movement: h, head; t, torso; p, pelvis; ᴖ, signifies horizontal movement; •, forward transport; ˪↑, vertical movement up with lose snout contact; =↑, vertical movement up in the air (Eilam and Golani, 1988).

The SoR of EWMN is designed so as to reflect the essential properties of the skeletal body plan. Its primitive is *a single movement:* a discrete change of angular relation between a part of the body in reference to its next heavy neighbor, requiring a single notational expression (Eshkol and Wachman, 1958) see notation of primitives in Figure 1, bottom. For example, a horizontal clockwise movement of the head of a mouse on its neck, starting in the midsagittal plane in prone position and ending 45 degrees to the right; see Figure 7, left column. Having defined the primitives of the system, They can be coordinated to generate modules, with morphogenetic generative rules (Figure 1, bottom).

Note that these primitives reflect the mechanical constraints on the anatomical body plan. The heavy and light limbs hierarchy is the highway leading to a natural representation of the mode of operation of the body plan, regulating its kinematic freedom of movement. By using a natural kind primitive, a view of a trans-phyletic geometrical manifold is obtained.

In the following review of the behavior, we adopt a tradition of comparative anatomy, by first describing the linear and modular (LM) morphogenesis of the behavioral *bauplan* during the recovery of movement in a vertebrate “monster.” Some features prevalent in this monster also prevail in an arthropod behavioral monster, indicating homology. To emphasize the heuristic value of monsters for highlighting generative rules we precede the description of the behavioral monster with a brief review of the use of monsters in classical comparative anatomy. Then, we show that LM characterizes locomotor buildup in recovery and in ontogeny. Importantly, we distinguish between the buildup in actual genesis (moment-by-moment time scale), and in ontogenesis (day-by-day time scale). In ontogenesis the buildup consists of a LM sequence of peak primitives, which culminated the daily buildup sequences. We then show that the primitives, modules as well as LM also prevail in the actual genesis of behavior in adult vertebrates, as they proceed out of immobility. Following the review of buildup, we show that the same generative rules, primitives and modules operate in reverse in the narrowing of behavior in stressful situations and, in both vertebrates and arthropods, with drugs. We end up this section of the essay by reviewing evidence for the same *bauplan* characterizing aspects of the phylogeny of vertebrate locomotion.

*Anatomical monsters.* For two centuries, an important line of evidence supporting the foundational role of morphogenetic homologous modules has been supplied by the study of abnormal or “monstrous” anatomical forms. Starting much earlier, with Aristotle, and then Goethe, who insisted that monstrous forms must arise in conformity with natural law (Goethe, 1830; Adelmann, 1936), and going on to Etienne and Isidore Jeofroy st. Hilaire (Saint-Hilaire, 1822; Saint-Hilaire, 1837), who demonstrated that abnormalities can be classified into well-defined types that illustrate morphogenetic principles. Taking a leap to more recent evo-devo literature, Opitz (1985) in medical genetics, and Alberch (1989) in embryology, articulate the advantages of analyzing the structure of abnormal anatomical phenotypes: different mutations tend to result in the same morphological anomaly, illustrating the final common pathway characterizing a morphogenetic field: the severity of a morphological deficiency reflects the stages of differentiation (e.g., degrees of skull reduction in guinea pig head deformation reflect successive stages of embryological differentiation). Most human malformations are midline anomalies of incomplete differentiation; anomalous anatomy highlights the anatomical invariance. A human infant’s tail, for example, draws attention to the midsagittal plane and its modular structure. Should anyone take for granted the existence of a modular spine or take its existence and structure for granted given the spinal requirements for flexibility and tensility, comes the human infant’s tail to instruct us that there is more to the spine’s architectural plan than its adaptive advantages; that intrinsic forces play a role in shaping the body, even when non-adaptive. The non-adaptive malformation prevents fabricated adaptive explanations; that which withstands the insult (e.g., the tail) is invariant and resilient; because the teratologies are generated in a discrete and recurrent manner, the order they highlight must be a reflection of endogenous constraints of the developmental field (generative rules); the deformity is never a mixture of types, highlighting the evolutionary invariant robustness of the modules; the pervasiveness of the same malformations across taxonomic groups implies homologies; the invariant order of loss of structures across instances implies terminal addition in development (as in progressive encephalization of the brain); and the “monster” highlights how much of the observed order is internally generated.

These features also apply to behavioral “monsters.” To introduce the behavioral *bauplan* in vibrant colors we therefore start with a description of the modular organization of a behavioral “monster.”

## 3 Collinear anteroposterior modularity

*The natural geometry of a behavioral “monster.”* When placed outside their cage following severe bilateral lateral hypothalamic (LH) damage, LH rats lie in a prone immobile position. When this placement procedure is repeated daily, the initial pronounced immobility is followed by movements that increase in extent. Day by day the movements start the same way, and day by day they increase in amplitude and complexity in a striking geometrical regularity, culminating in the daily sequence with the terminal addition of a new movement type(s). The recovery of movement can be conceived as a matrix comprising rows of daily sequences of actualgenesis (*actualgenese*), culminating in a terminal column representing the newly-recovered movement type(s) (followed by movement types which had appeared earlier on). This process of motorial expansion, later also established in intact infant rats (Golani et al., 1981; Eilam and Golani, 1988), and in adult animals (Golani, 1992a, 2012), has been termed a “warm-up” sequence.

In “warm-up,” movement propagates from head to tail in a linear order on the AP axis of the trunk, along each of the spatial dimensions separately (Golani et al., 1979).

**Figure 8:**
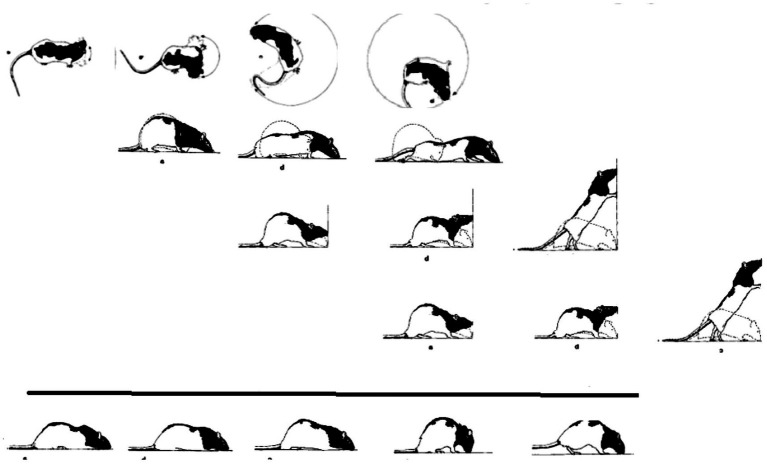
A schematic illustration of the separate AP buildup of movement (from left to right along the lines) involving recruitment of the next caudal part(s) of the trunk along each module separately, and the successive emergence of modular spatial dimensions, represented by horizontal lines, from top to bottom: horizontal, then forward, then vertical along surfaces, then vertical in the air. Concurrently, the “support” module also recovers anterior-posteriorly (bottom horizontal line from left to right). After Golani et al. (1979).

- *Horizontal on hindlegs dimension*: Each and every warm-up sequence is initiated by small amplitude side-to-side horizontal head movements. During early recovery, the daily warm-up sequence starts, proceeds, and ends with this type of movement (type defined by the anatomical part and the spatial dimension in which it moves). Later on in recovery, horizontal movement propagates in a linear AP order along the trunk, culminating daily in the performance of progressively more caudal parts in horizontal movements (Figure 8, 1st row).
- *Forward dimension:* Forward head-and-neck transport is typically initiated only after the exhaustion of the horizontal plane. It foreshadows progressively larger amplitude movements, culminating in full-blown movements recruiting in an AP linear order the whole body in forward progression (Figure 8, 2nd row).
- *Vertical-on-hind-legs-with-snout-contact dimension*: The same AP generative rule applies to increasingly more advanced sequences, exhibiting vertical positive movement involving snout contact, which ultimately culminate in full-blown rearing vis-à-vis vertical surfaces (Figure 8, 3rd row). Later still, the daily sequence still starts with horizontal movement around the hind legs, commences across the forward dimension, then positive vertical movement with snout contact unfolding along the trunk (head raising, rearing), followed by the same cascade of vertical movements but this time without snout contact. In older infants and in adult animals, during interactional play, vertical rearing on hind legs extends into tumbling backwards during head-to-head contact maintenance (Figure 1, vertical on hind legs with snout contact; Figure 11 in Havkin and Fentress, 1985).
- *Vertical-on-hind-legs-without-snout-contact dimension*, again involving progressive AP recruitment of the parts of the trunk, first *vis-à-vis* nearby vertical objects, then *vis-à-vis* increasingly distant vertical objects (Figure 8, 4th row) (Golani et al., 1979).

The generative rule applying to the moment-by-moment (actualgenesis) build-up is: *a movement of a part of the trunk is not performed along a dimension unless the part of the trunk anterior to it has already moved along that dimension, and unless that part has already moved in the current sequence along the previously prescribed dimension* (*horizontal, then forward, then vertical with, and then vertical without snout contact*). In the full-blown version of this recovery process, each dimension is exhausted and then repeated multiple times before the transition to the next dimension. The primitives of the process are horizontal and vertical movements of the parts of the trunk and corresponding stepping, supporting movement along the four dimensions. These primitives exhibit (linear) recruitment along each dimension *separately*, implying separate “book-keeping” for each dimension, i.e., modularity. As with anatomy so too with movement: modularity refers to a pattern of connectedness in which the primitives are grouped into highly connected subsets – that is modules – which are less tightly connected to the other subsets. The coordinative movement synergies, which unfold along discrete spatial “dimensions” are referred to henceforth as “modules.” The extended connectedness marks this modular locomotor recovery as a candidate homology, prompting its search in other preparations, taxa, and contexts.

Several features warrant the “monstrous” nature of this behavior: first, the *large amplitude delay in recruitment of the next caudal body part*, highlighting the magnitude of this delay as a relevant kinematic variable, and second, the *perseverative non-adaptive* nature of the behavior. The delay in recruitment is also manifested in intact animals during stressful situations (Golani and Moran, 1983) and in the phylogenetically remote fruit flies treated with the dopamine reuptake inhibitor, cocaine. Notably, in the adult fly, where movement between segments of the rigid trunk is impossible, the delay is manifested instead in an increase in the angular interval between the direction of the fly’s trunk orientation and its direction of progression (Gakamsky et al., 2013). Below we suggest that this delay in recruitment is a homologous built-in “final common pathway”; a recurrently common, fundamental kinematic constraint on the freedom of movement. The non-adaptiveness of the behavior is demonstrated by placing the LH rat in an open corner at, e.g., the stage of partial recovery of the forward progression module. The constrained forward progression module dooms the rat to perseveratively pivot, progress slightly out of the corner, back off, pivot again, etc., seemingly leaving the corner yet never actually accomplishing departure (“behavioral trap” phenomenon Golani et al., 1979). A third instructive lesson conveyed by this behavioral monster is that the failure to leave the corner *also involves a deficit in arousal*, and hence in repertoire size and amplitude: a tail pinch, tactile input from the encountered corner’s walls, or proprioceptive input emanating from the animal’s own attempt to extricate itself, increase the repertoire and amplitudes of movements to amounts that are not sufficient for extrication yet larger than those exhibited in the open space, where a physical challenge is absent (Wolgin and Teitelbaum, 1978). A similar transient buildup in mobility and arousal is generated by human parkinsonian patients using self-generated proprioceptive input to extricate themselves from a freezing episode (Menken, 1988; Sacks, 1991) (see also Supplementary Figure 5). All in all, the monster portrays the organism’s morphogenetic potential, the concealment of this potential in the absence of a challenge, and, once this potential is exhibited, its ephemerality. We will return to these features below, in our discussion of the function of exuberant behavior, by suggesting that peak mobility movements generate proprioceptive feedback that liberates the organism from the dominion of both external and internal stimuli, allowing it to act (or not to act) freely.

The non-adaptiveness of the monster’s behavior prevents explaining it away by means of speculative adaptive stories (Lewontin, 1979). Nor would it make sense to assign the seemingly bizarre postures a biosemiotic meaning. The exhibited form asks for a morphogenetic explanation. Presenting these results in the context of anatomical monsters highlights the instructive power of behavioral monsters and supports the notion that behavior is an extension of anatomy.

*Collinearity and modularity in the actualgenesis and ontogenesis of locomotor behavior in infant rodents and in other Tetrapoda*. If there is at all a behavioral *bauplan* shared across the vertebrates, then its primitives, modules and generative rules should be manifested in recovery and in ontogeny alike. Ontogeny and even more so actualgenesis in infancy also provide an appropriate period for the study of morphogenesis because of the relatively slow and gradual buildup of behavior characterizing young age.

Extended immobility provides an opportunity to study the moment-by-moment growth in extent and differentiation of locomotor behavior in intact infants (Golani et al., 1981; Eilam and Golani, 1988; Golani, 1992a). As with lesioned adult rats so too with intact infant rats - placing one outside its nest induces immobility. Following pronounced immobility, the infant exhibits a sequence whose repertoire of movements is characteristic of the developmental day. (*i*) *Horizontal on hind legs module* develops first, (*ii*) *Forward module* second, (*iii*) *Vertical-on-hind-legs-with-snout-contact* third; and (*iv*) *Vertical-on-hind-legs-without-snout-contact module* last (Golani et al., 1981; Eilam and Golani, 1988) (see Figure 7). Along each dimension separately, “buds” of small amplitude movement, recruiting only the head, foreshadow progressively larger amplitude movements ending in full blown movements recruiting the whole body in linear AP order. The following warm-up sequence (demonstrating a sequence of actual genesis) starts with horizontal movement that exhausts the horizontal plane before the budding of increasingly longer forward movement, culminating in vertical head movement in the air (in the absence of nearby walls, vertical movement with snout contact is skipped; Supplementary Video 1).

A similar exhaustion of the horizontal plane before forward movement is added to the repertoire is illustrated in Supplementary Video 3, depicting the actualgenesis of movement of a barn owl fledgling placed outside the nest.

**Figure 9:**
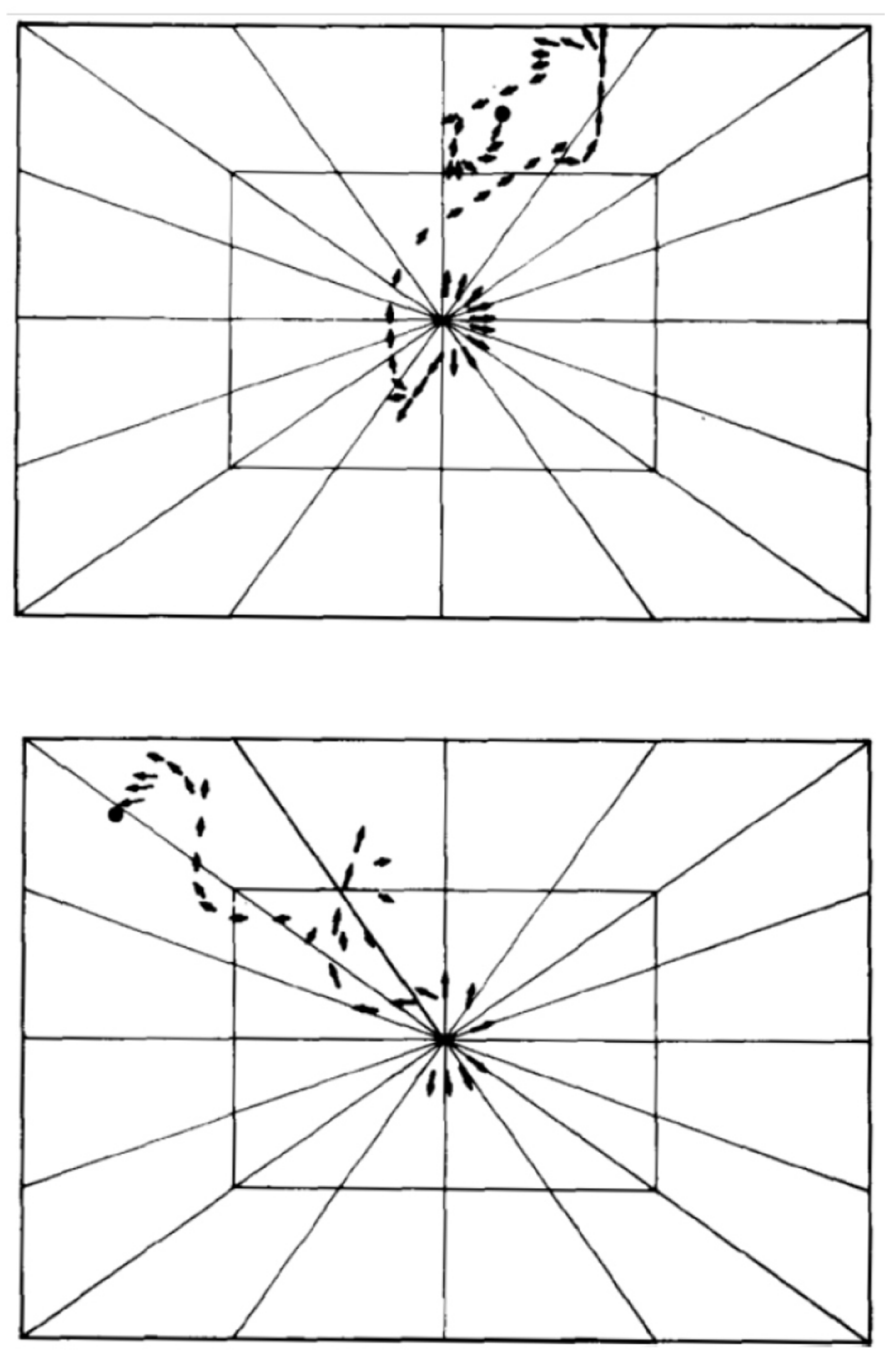
The locations and positions of the torso on the observation platform are traced from film, in 10 frame intervals, in two daily sequences of 14-day-old rats. Recording started with initial immobility and ended with the performance of the first vertical movement of the head (signified by a dot). From Eilam and Golani (1988).

During the performance of warm-up the infant rat may: (a) repeat the same movement type; (b) revert to movement types performed earlier; (c) proceed to incorporate the next heavier segment in movement along the same dimension, or, (d) move with the lightest segment (the head) along a new, more “advanced” dimension (Eilam and Golani, 1988). A “byproduct” of this kinematic process is the scanning, first of the proximal environment, and then of increasingly larger areas (Figure 9).

**Figure 10:**
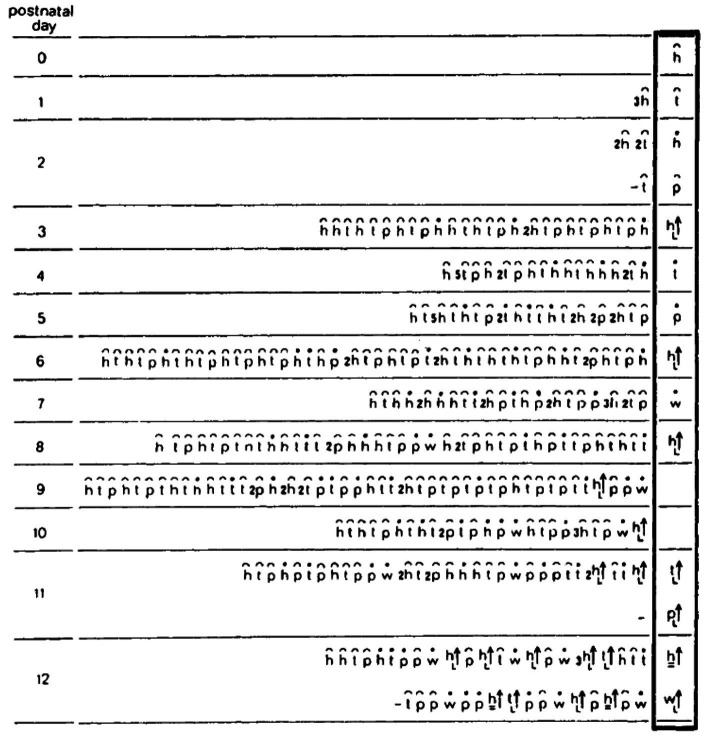
“warm-up” in actual genesis and in ontogeny. The head-to-tail sequence of initiations of movements of the “heaviest” part of the trunk, separately along each of the first 4 spatial modules, horizontal first, forward next, vertical with and then without snout contact. Each line presents the most advanced sequence of each developmental day. Horizontal lines distinguish between daily sequences. (Distinction between phrases is eliminated). ᴖ, signifies horizontal movement; •, forward transport; ˪↑, vertical movement up with lose snout contact; =↑, vertical movement up in the air; w, forward walking; w˪↑, climbing; h, head; t, torso; p, pelvis. From Eilam and Golani (1988).

The build-up in extent and the differentiation are thus monotonical in the broad sense: once a movement along a new plane or a movement of a more caudal part is performed, these movements become part of the animal’s locomotor repertoire for the rest of the sequence, being performed unpredictably with more advanced movement types. *Collinearity thus applies to the addition of new movement types* (Eilam and Golani, 1988). The process involves a progressive build-up of the animal’s movement repertoire. Since the “warmup” sequence is performed *de novo* following extensive immobility, the developmental day-by-day sequences yield the most advanced movement types that culminate the daily “warm-up sequences” (Figure 10). Note, that whereas in the classical ethological type of sequence, syntactical connectedness is limited to first-, or mostly second- or third-order transitional probabilities across building blocks, in the mobility sequence build-up, which is also a type of sequential connectedness, connectedness may extend across days, weeks or even months, as in anatomical differentiation. Such morphogenetic build-up has not been reported in actualgenesis for any sequence of ethogram-listed building blocks.

Since the matrix of daily warm-up sequences is defined topologically on the basis of the invariant connectedness among the primitives (movement types) (Saint-Hilaire, 1822), the movement types, the spatial modules, and the whole behavior exhibited in recovery are serially homologous to the behavior exhibited in ontogeny [serial homology is a special type of homology, defined as “representative or repetitive relation in the segments of the same organism (Owen, 1846), like the hands and the feet of a human”]. The cascade of modules constituting this behavior has been termed “the mobility gradient” (Golani, 1992a, 1992b).

*The transition from weight on hindlegs to weight on forelegs in actualgenesis and ontogenesis.* As soon as the infants become juveniles there is an increase in the extent of movement and differentiation to additional modules. This increase is exhibited in interactional play in the third, *Vertical-on-hindlegs-with-snout-contact* module, such as when an animal rears on its hindlegs and then may commence to roll back pelvis-first (Figure 1 bottom, vertical on hind legs with snout contact). *Along this spatial behavioral module, the heavy and light limbs hierarchy proceeds from hind to forequarters, and movement proceeds in a linear Antero-Posterior* (*AP*) *direction.*

A build-up into two new modules unfolds in play and fighting situations as the animal shifts weight to its forelegs, exhibiting the.

**Figure 11:**
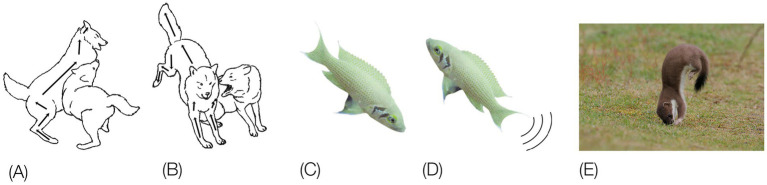
During so-called ritualized fighting interactions, golden jackals (*Canis aureus*) **(A,B)** and cichlid fish (*Neolamprologus pulcher*) **(C,D)** change the vertical orientation of their trunk. The free animal (often described as superior), rears on its forequarters but may also rear on its hindquarters (**A,B**, left jackal; and fish in **C**). The constrained animal rears only on its hindquarters (right jackal in **A,B**, fish in **D**). **(C,D)** are reproduced with permission of Drs. Sigal Balshine and Adam Reddon, McMaster University (Reddon et al., 2019). **(E)** During play or “war dance,” stoat rears on forelegs (courtesy Tristan M. Bantock).

- *Horizontal on forelegs module* [pivoting around the forelegs (Yaniv and Golani, 1987); see Figure 1 horizontal on forelegs; Supplementary Figure 1A] and the.
- *Vertical on forelegs module* (handstand; rodeo posture in horses; Golani, 1992a) (Figure 11B left jackal), rearing on forelegs during “war dance” in stoats (Figures 11E), culminating in tumbling head-on; also Figure 1 bottom, vertical on forelegs). The last two modules emerge both in ontogenesis and in actualgenesis concurrently and may be performed simultaneously. The last two modules may belong to one and the same module of shift of weight to forelegs and release of hind feet contact with ground (as in a kicking horse). The homological counterpart of rearing on forelegs in quadrupeds (Figure 11B left jackal), is shifting weight to forequarters (head down posture or even standing on head in fish (Figure 11C). The homological counterpart of rearing on hind legs in quadrupeds (Figure 11A left and right, B right jackal) is shifting weight to hindquarters (head up posture in fish (Figure 11D). Along the last two spatial behavioral modules the heavy and light limbs hierarchy proceeds from fore to hind quarters, and movement proceeds in a linear *Anterior–Posterior* (*AP*) direction. (compare to same, topologically equivalent, geometry, applying in the *Collinearity and modularity in the phylogeny of vertebrate adult forward progression* in chapter carrying this heading).

The freedom to rear either on hindlegs or on forelegs, versus the absence of rearing on forelegs in the constrained partner is illustrated also in ritualized fighting of wild Norwegian rats (Golani 2015b; Supplementary Video 4).

These behaviors first require actual physical snout contact or near contact (opposition) with the partner (e.g., during fighting in rodents and in canids) but may then also be exhibited “in solo” during what has been termed “exuberant locomotion” (Lorenz, 1981). High mobility rearing and/or pivoting on forelegs is exhibited, for example, by ungulates when bucking off a rider in a rodeo, or in the wild when throwing a big predator off their back (Supplementary Video 5).

Needless to say, the functional aspect of the behavior should not dissuade one from acknowledging its form and its sequential position along the mobility *bauplan*. The very same forms are also exhibited in the absence of a rider or a predator (Supplementary Video 6) and hip slamming a rival during an interaction (free wolf in wolves; Golani, 1981). The vertical on forelegs module culminates in tumbling head-on in play fighting (Figure 1, 5th row), allowing the free animal mouth-access to the forelegs of the constrained opponent, which may be bitten, and in a real fight also broken (e.g., wolf cub in Figure 12 in Havkin and Fentress, 1985; Golani, 1992a). This behavior is performed in a variety of functional contexts, such as urination (Supplementary Video 7).

- *Simultaneous release of all four feet contact with ground.* Ever more striking types of movement, involving release of all feet-contact with the ground, such as in stotting or pronging, log-rolling and tumbling in the air, are exhibited at the peak of exuberant behavior, begging for a functional interpretation that would be implied by and consistent with the essential features of the *bauplan* (see our liberation hypothesis below).

**Figure 12:**
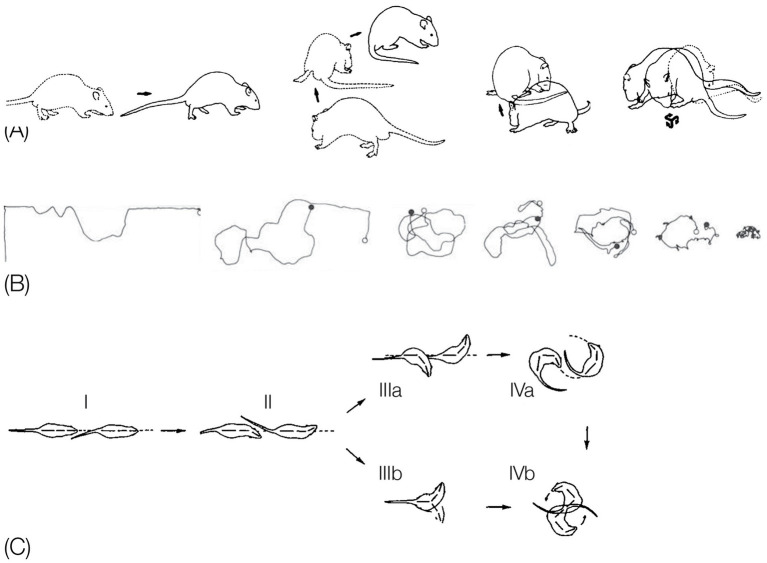
**(A)** In rats treated with the dopamine agonist apomorphine (1.25 mg/kg) the spatial modules are enhanced and then eliminated in a fixed order (schematically illustrated from left to right): vertical first (not shown), forward second, path curvature is thereby gradually enhanced to a maximum, pivoting on hind legs is enhanced and then decays linearly from hind- to forequarters, and support dwindles from hind to forequarters (after Szechtman et al., 1985). **(B)** Seven representative path segments traced by the snout of a selected rat at successive 15 s intervals during the course of apomorphine action illustrate, from left to right, the regressive narrowing of attended space (After Szechtman et al., 1985). **(C)** A schematic illustration of the superposition of horizontal trunk movements on forward progression in rats injected with 5 mg/kg amphetamine. Interrupted lines represent the path traced in the environment. Roman numerals designate the composite locomotor patterns observed and their order of performance. In the sequence illustrated in **(A)** forward progression is eliminated after the onset of horizontal lower torso movements; and in **(B)** before the onset of forward progression (after Adani et al., 1991).

*Modular build-up in ontogeny from fish, across amphibians to humans.* The ontogenetic transition from horizontal (lateral) movement to forward progression has been demonstrated across the vertebrates, from primitive species all the way to human infants. It has been elucidated, for example, in Coghill’s classical description of the ontogeny of movement in the axolotl (*Ambystoma*; Figure 6). Following extended immobility, the embryo performs single, increasingly larger forequarter horizontal (lateral) movement to one side at a time. Forward progression emerges when two horizontal movements on the left and right sides of the body follow each other in proper phase delay (S-reactions; forward swimming; Coghill, 1929) The same sequence has been reported in fish (Tracy, 1926; Eaton, 1992).

At the other end of the phylogenetic scale, the following sequence has been reported in human infant ontogeny: first pivoting (week 29), then backward crawling (week 31), then forward crawling (week 34), then creep-crawling (37 weeks), standing (56 weeks) and bipedal walking (60 weeks), recruitment of trunk parts “sweeping from head toward the lower trunk, pelvic and sacral regions, and… progressing outward in the limbs from shoulder to finger-tips, and from hips to toes…” (Shirley, 1933; Gesell and Ames, 1940).

*Linear and modular build-up in adult actualgenesis.* Following extensive immobility, many adult vertebrate species perform a warm-up sequence, typically exhausting the horizontal plane several times before incorporating forward transport into the sequence. The initial immobility, the recovery of support in the forelegs and only then in the hind legs, and the performance of more than 740° in pivoting before incorporating forward progression is evident in an adult desert hedgehog (*paraechinus aethiopicus*; Supplementary Video 8).

Following an ambush culminating in the successful bite of a mouse, a horned viper also performs a warmup sequence (Supplementary Video 9). The adaptive explanation – first letting the venom take its action, and only then searching for the footprints of the bitten runaway mouse and following them to the already dead mouse – should not dissuade us from noting the extended immobility following the strike, and the fact that a modular, collinear AP order of recruitment of the trunk segments applies also during pivoting in place while sidewinding.

In some species, an additional module of backward locomotion is performed. It is illustrated here in a salamander (Supplementary Video 10). Backward crawling has also been reported in human infant ontogeny, following the development of horizontal movement (pivoting; Gesell and Ames, 1940).

*The locomotor modules are relatively homogenous during early morphogenesis, and become increasingly mixed with other modules during later stages.* The younger the animal, and the more extensive the initial immobility, the greater is the repetition of a movement type, yielding extended bouts of same-type (stereotyped, see Golani et al., 1999) movement before proceeding to the next type along the body, within the same module, yielding extended relatively homogenous bouts (Eilam and Golani, 1988). Thus, for example, infant vertebrates tend to repeat side-to-side head movements for long bouts before recruiting side-to-side chest movement, and tend to exhaust the horizontal plane and repeat it until it becomes “well-trodden,” before moving forwards (Supplementary Videos 2, 3). Modularity can thus be fully unfolded into separate modules, whereby transition from one dimension to the next takes place only after exhaustion of the previous dimension in the prescribed sequence. During later stages of morphogenesis, the spatial locomotor modules become increasingly mixed and inhomogeneous. Such transition from highly modular sequences, consisting of separate, homogenous modules, to increasingly mixed sequences has been described by Bohm as a transition from explicated or unfolded order to an implicated or enfolded order (Bohm, 1981). The transition from an explicated to an implicated order in morphogenesis is why the connectedness (Saint-Hilaire, 1822) of behavior is evident early in actualgenesis and ontogenesis, becoming increasingly blurred as the behavior enfolds or becomes implicated at later stages. This is perhaps why connectedness and therefore behavioral homology could not be demonstrated by Lorenz in duck display sequences (Beer, 1974; Lorenz, 2012): the movement material Lorenz studied consisted of full blown, implicated, and therefore, seemingly haphazardly ordered sequences. Connectedness cannot be demonstrated compellingly without studying the morphogenesis of the sequence, tracking back the transition from adult implicated order, back to juvenile and then to infantile explicated order. With fairness to Lorenz we should recognize the paucity, at the time, of technological means to track, store and analyze sequences of actual genesis of movement across ontogenesis. These alleviating circumstances are not admissible, however, for most current day computational phenotypers who choose to restrict the study of the connectedness of behavior to full blown implicated behavior.

Morphogenesis folds in reverse during narrowing of the repertoire of modules. The majority of anatomical morphogenetic processes involve growth, differentiation, and unfolding of structure - forward life processes increasing the organism’s viability (see, however, reversal of morphogenesis in Hydra, Braun and Ori, 2019). In contrast, the actual genesis of movement-based behavior consists of both, build-up and narrowing of freedom of movement: unfolding and folding of movement, both along the body and across modules, manifesting both forward and backward bouts of morphogenesis. These fast alternations in direction should provide evo-devo with a novel experimental view of morphogenesis unfolding and enfolding within seconds.

*Drug-induced and neurological “behavioral monsters” in a vertebrate:* High-dose drug-induced behavior often shares with teratological preparations the absence of functionality and exaggeration of core structural features, which are less conspicuous in intact behavior. The repertoire of modules, which builds up in intact rats by terminal addition (Eilam and Golani, 1988), is narrowed by first enhancing and then eliminating module after module, reflecting the “last in first out” rule, following injection of the dopamine agonist apomorphine (1.25 mg/kg s.c. neck injection; Supplementary Video 11). Concomitantly, attended space gradually shrinks (Figure 12B).

The same order of modular enhancement and then elimination is exhibited with regard to forward progression and horizontal movement, following injection of (+)-amphetamine (5 mg/kg), a dopamine releasing drug (Figure 12C). Variations in the relative timing of onset and termination of the appearance of these two modules account for the multiple patterns observed (Figure 12C) (Adani et al., 1991). While the narrowing process culminates under apomorphine in a shift of weight backwards without stepping, under high doses of amphetamine narrowing culminates in backward running (Fernando et al., 1980).

*Drug-induced and neurological “behavioral monsters” in human pathology:* The significance of fully documented manifestations of the spatial modules in pathological human movement-based behavior is an indication of their very existence in man’s repertoire. These manifestations require, however, a systematic mapping, which does not exist so far, of the morphogenesis and connectedness of these modules.

One pathological manifestation of the vertical upward (positive) module is the Oculogyric Crisis (OGC), the clinical phenomenon of sustained dystonic, conjugate and typically upward deviation of the eyes lasting from seconds to hours. It was initially observed in patients with post-encephalitic parkinsonism, following the epidemy of *encephalitis lethargica* (Economo’s disease) during the 1910–1930s, and in particular following the administration of L-Dopa (Jelliffe, 1933; Sacks and Kohl, 1970; Barow et al., 2017). The OGC manifests the characteristics of a spatial module, including AP linear propagation from eyes, to head and then to neck, involving a psychomotor shift of visual attention upward (vertical on hindlegs in Figure 1 bottom).

We also found a single documentation of a whole-body saltation pelvis-on, involving an AP linear recruitment of all the parts of the trunk, ensuing in the performance of 3 × 360° saltations backward (Figure 1, vertical on hind legs module, bottom figure, 4th line from top). The behavior has been performed by a soccer player following a kick to his neck. The player has been reported to fit to play in the next game, scoring two goals (Abdulrahman Al-Shoaibi - Wikipedia). This episode suggests an activation of a built-in, well-coordinated vertical positive module. To our knowledge, there is presently no other explanatory context that can make sense of this, seemingly out-of-context behavior, except for the model of the built-in spatial modules of movement portrayed in this essay.

**Figure 13:**
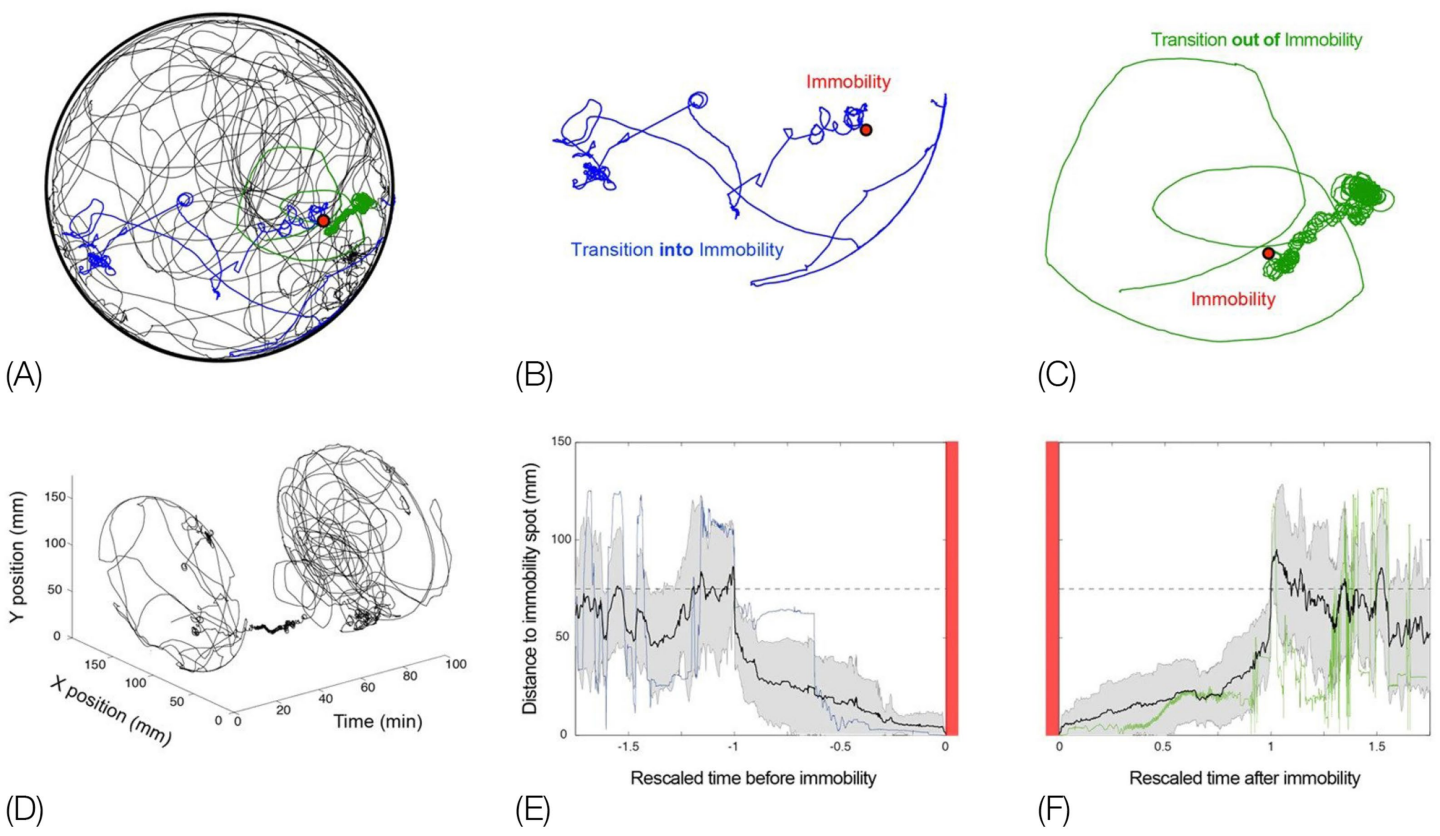
The locomotor path of cocaine treated flies progressively narrows into immobility and then builds-up to exhibit normal locomotor behavior. **(A)** A fly’s path for the entire 90 min session in a circular arena. Red dot indicates location of immobility. Blue path depicts transition into immobility, and green - transition out of it. The rest of the trace throughout the experiment is represented in black. **(B)** Same path as in **(A)** unfolded in time. Transition into and out of immobility is clearly visible, and is used as the point of reference for examining the phenomenon. **(C)** The transition into immobility (corresponding to the blue path in **A**) is marked by the performance of straight paths, and then by increasingly curved paths, narrowing the spatial spread of the animal’s path. **(D)** The transition out of immobility (corresponding to the green path in **A**) is marked by the performance of initially curved, and then increasingly straighter paths, building up the spatial spread of the animal’s path. **(E)** Distance of each fly to its corresponding immobility spot as a function of rescaled time. The average trend of activity (black line presents the mean for all flies, gray area depicts SEM) reveals a consistent narrowing of the path during transition into immobility (alignment marked by the vertical red bars), and **(F)** a build-up of the path for all flies during transition out of immobility. In **(E,F)** blue and green traces correspond to the example from **(A–D)**. Despite single fly variability being high the further away it is from immobility, the closer to immobility (both before and after) the more progressive and slow the spatial spread dynamics become. Note the narrowing before immobility and the build-up after immobility of the span of traversed space (after Gomez-Marin et al., 2016).

**Figure 14:**
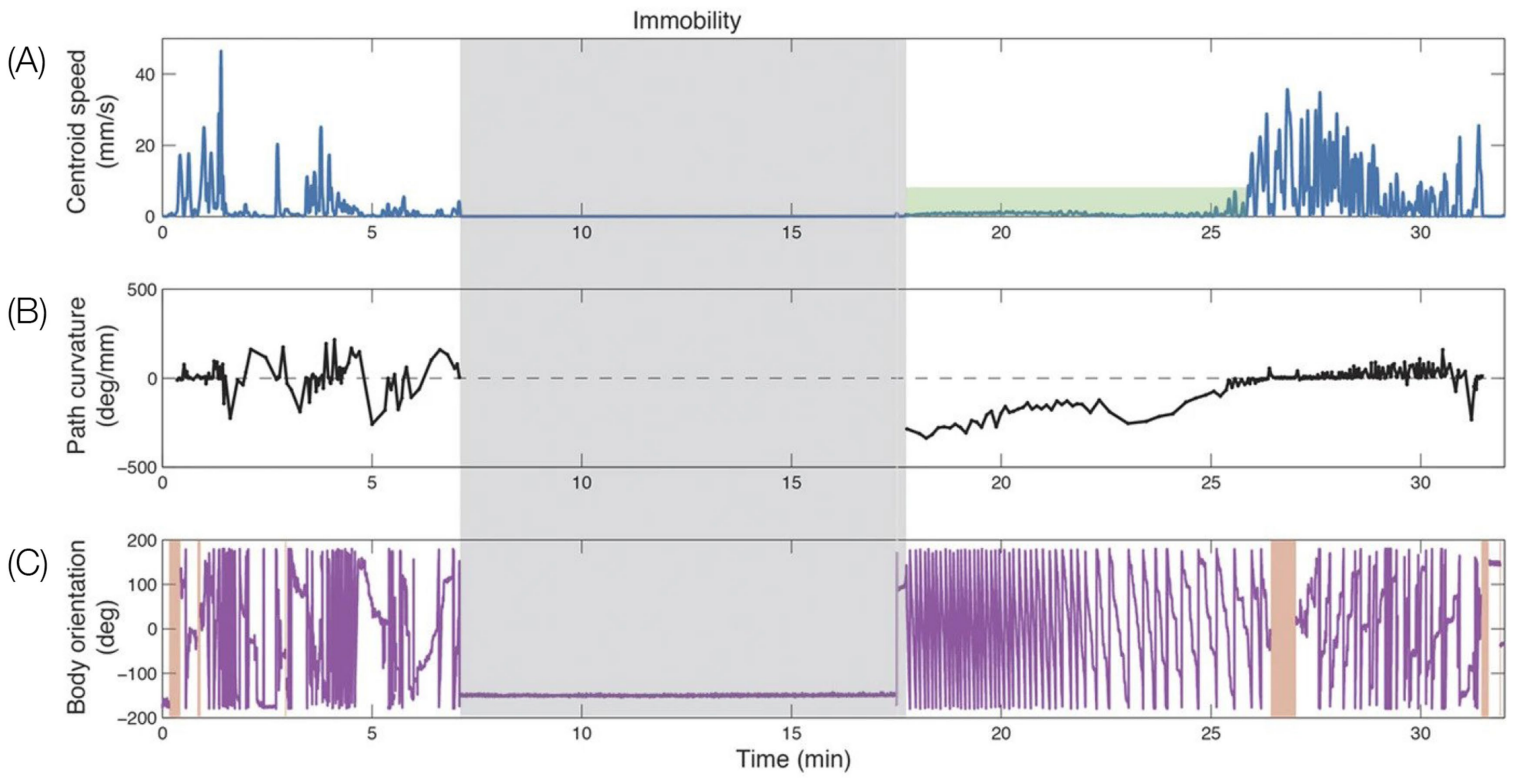
Representative moment-to-moment dynamics of the three kinematic degrees of freedom, before and after immobility, of the same fly path session plotted in Figure 6 in Cartesian space. The shaded area marks the period of immobility, which is used as a reference for the events that precede and follow it. The session starts with bursts of speed that progressively decrease toward zero **(A)**. The straight path is followed by bursts of high curvature until immobility **(B)**. Extensive whole body horizontal movement (pivoting) leads to immobility **(C)**. Following a 10-min period of complete immobility, very low speed **(A)**, is then accompanied by high curvature **(B)**, and extensive pivoting **(C)**. Build-up culminates in high speed **(A)**, straight path **(B)**, and low change in body orientation **(C)** (after Gomez-Marin et al., 2016). While trans-phyletic behavioral homology and the molecular dopaminergic stimulation mediating this behavior are conserved across phyla, it is still been debated whether there was a deep homology between the respective neural substrates (basal ganglia and central complex) that appeared to mediate the behavior (Strausfeld and Hirth, 2013; Varga et al., 2017).

*Drug-induced and neurological “behavioral monsters” in an arthropod:* Remarkably, the same modules and generative rules of narrowing and of build-up observed in rats injected with dopamine agonists are also exhibited in fruit flies: when the dopamine re-uptake inhibitor cocaine is used as the parameter inducing progressive transitions in and out of immobility in *Drosophila melanogaster*, narrowing down into immobility is accomplished by first enhancing and then eliminating forward progression, followed by enhancement and then elimination of horizontal whole body movement (Gomez-Marin et al., 2016; Supplementary Video 12). The kinematics of narrowing and build-up are plotted in Figures 13, 14 and illustrated in Supplementary Video 13.

An intriguing issue examined in evo-devo is whether the hypothetical common ancestor of the bilaterian clad, i.e., of all animals having a bilateral symmetry including arthropods and vertebrates (dubbed *Urbilaterian*), had a central nervous system, or a diffuse ectodermal nervous system. Has a central nervous system evolved just once, as now debated by many, e.g., (Hirth et al., 2003; Arendt et al., 2008; De Robertis, 2008; Tomer et al., 2010; Holland et al., 2013; Sen et al., 2013), or twice, as now maintained by few (e.g., Gerhart et al., 2005; Pani et al., 2012)? The view that the ancestral, urbilaterian animal had a CNS is consistent with the view that the CNSs of all metazoans are homologous. Needless to say, the demonstration that fruit fly cocaine-induced behavior, cocaine being a dopamine reuptake inhibitor, shares with rat cocaine-induced behavior the same topological connectivity, strongly supports the hypothesis that the respective CNSs of vertebrate basal ganglia and arthropod central complex, are homologous, implying an urbilaterian ancestor endowed with a CNS. Note, that the behavior becomes a criterion implying an underlying anatomical homology because of its topological content, which appears to reflect the anatomical and physiological connectedness of its anatomical substrate. Could a dopamine feed-forward loop akin to the one described by Ikeda et al. (2013) in the vertebrate striatum also function in the arthropod central complex? To examine this and similar hypotheses relating neural connectivity to kinematic connectedness, it would be necessary for students of Evo-devo to embrace the view that behavior is an extension of anatomy, and dare using a literate geometric approach to behavior, as they already do with anatomy.

Reviewing the fruit fly larval behavior literature with a search image for low and high mobility, attention is immediately drawn to the abnormally high extent of turning behavior exhibited by larvae with mutations in the gene scribbler (sbb) in the absence of food (Sokolowski, 2001). These appear to be respective manifestations of the high and low ends of the mobility gradient.

The four key features characterizing low mobility in the cocaine-treated fly (low speed of translation, highly curved path, high body rotation, and immobility) exhibit a full correspondence to the features of the “abnormal crawling pattern” exhibited by scribbler larvae: low speed, curved paths, high turning rate, and long pauses. The parameter precipitating this behavior could be, as Sokolowski and co-workers suggest, the absence of food, or else, given our search image, the stress brought about by the absence of food, or even its presence in hungry flies. Equivalent differences in mobility, expressed by pivoting and/or rearing on hind legs and forelegs, or only on hind legs (Figures 11A,B) reported to be exhibited by vertebrate partners engaged in interactions, might also be looked for in fruit fly courtship and agonistic interactions.

*Stress-induced narrowing of kinematic freedom of movement.* In the same way that different mutations and treatments tend to result in the same morphological anomaly, illustrating the final common pathway characterizing a morphogenetic field (Opitz, 1985; Alberch, 1989), the kinematic narrowing of freedom of movement exhibited in vertebrates consists of the same primitives, modules, and generative rules, in animals treated with dopamine agonists, and in animals responding to naturally occurring stressful situations.

The “last in first out” rule of dropping out of action of the parts of the trunk following injections with dopamine agonists, also applies to the reversed modular linearity exhibited by the so-called “inferior” quadruped animal during “ritualized fighting” interactions. The order of inactivation with the drugs is the same as that exhibited in an intact inferior individual under stress induced by the approach of a superior rival (Moran et al., 1981; Golani and Moran, 1983; Yaniv and Golani, 1987), or by entry into a novel environment (Golani et al., 1981; Eilam and Golani, 1988; Hager et al., 2014), or in situations involving transition into immobility, such as the shift from forward progression to circling to pivoting and to squatting that precedes defecation in canids (Golani, 1992a), or the pivoting that precedes lying down to sleep in canids (Golani, 1992a). During ritualized fighting interactions, one interactant, commonly labeled the “superior,” exercises full freedom of movement of all the parts of the body along all the spatial modules, regardless of distance and relationship of opposition (near contact) to its (inferior) partner (Yaniv and Golani, 1987). In contrast, the constrained interactant, the so-called “inferior” (Schenkel, 1967), exhibits, in tandem with the reduction of distance between the partners and the establishment of a specific near-contact relationship between the superior’s teeth and its own vulnerable body parts, a lawful narrowing. This includes a transition from locomotion on toes to locomotion on soles and toes (from digitigrade to plantigrade), from forward to horizontal movement around the hindquarters, then to full shutdown of stepping with hind legs, then to squatting of hindquarters, pivoting in place, elimination of negative vertical movement (no lowering of the trunk below the absolute horizontal plane; viewed from hind- to forequarters) and finally to crouched immobility. As soon as the distance from the opponent increases, immobility dissipates in an AP order (ritualized fighting in wolves, Supplementary Video 22, Golani and Moran, 1983), honey badgers, Tasmanian devils (Eisenberg and Golani, 1977; Golani and Moran, 1983; Yaniv and Golani, 1987). The contrast between the freedom to perform horizontal and vertical whole body movement around both the hind- and forelegs by the free partner, and the absence of freedom to move within these two modules by the constrained partner, is evident in the so-called “ritualized” fighting interaction between the two jackals presented in Figure 11 as well as in the video of a similar interaction in two wild Norwegian rats (Gomez-Marin et al., 2015; Supplementary Video 4).

**Figure 15:**
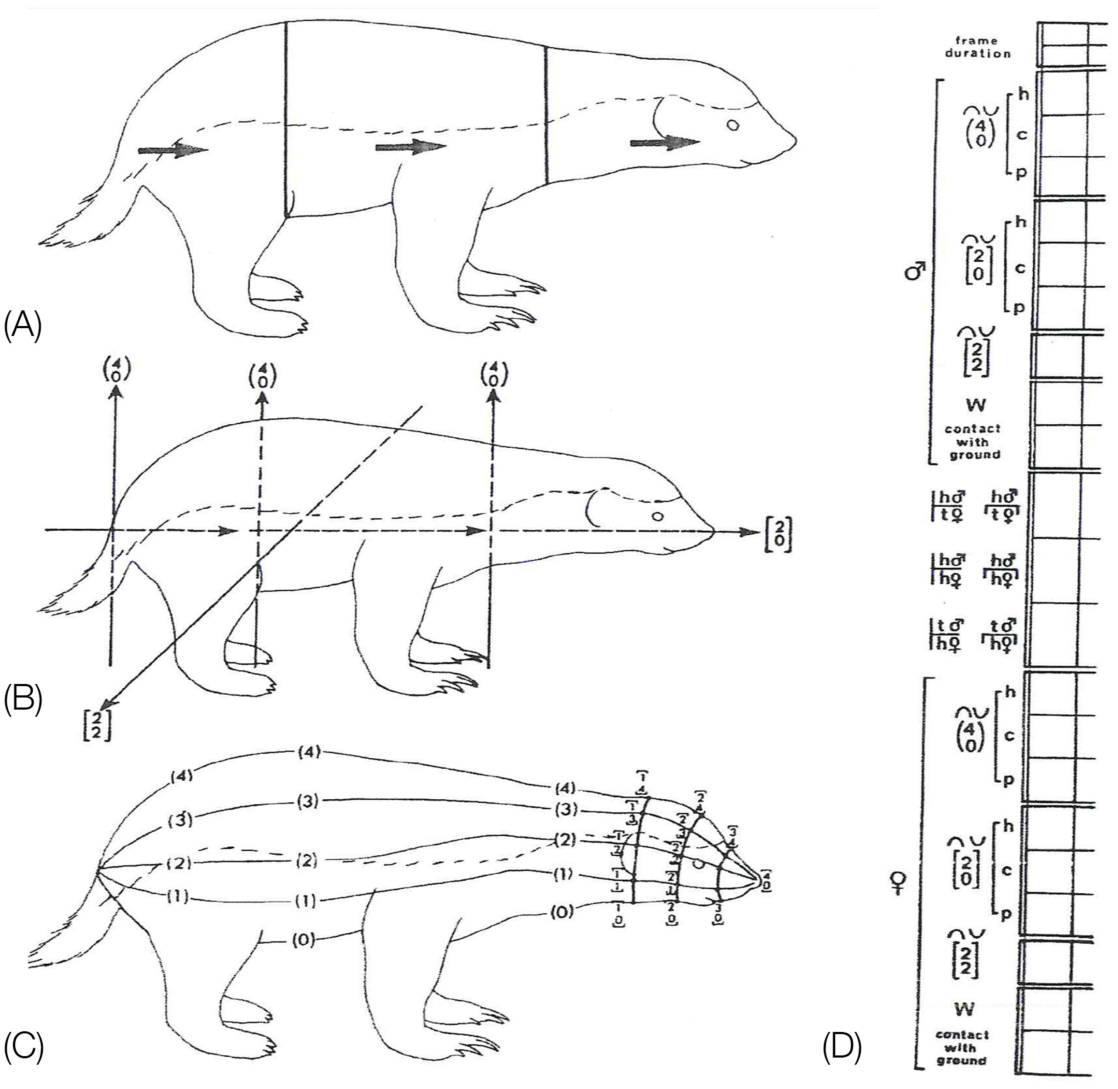
The geometrical frame used to analyze and represent Honey Badger (*Mellivora capensis*) interactions. **(A)** The law of heavy and light limbs applied to the parts of the badger’s trunk. Arrows indicate the relation “from heavy to light” when the badger performs a whole-body rotation on the hind quarters. **(B)** Movement is described in reference to the three axes of rotation and their location in reference to the AP axis. These 3 whole body rotations stand respectively, from tail to head, for horizontal movement around the hindquarters, around the center of the trunk, and around the forequarters. Whole body rotation around the side-to-side axis stands for vertical positive (tumbling head-on), and vertical negative (tumbling pelvis-on) modules. **(C)** The surfaces of the badger’s trunk and head seen as modified spheres with EWMN coordinates. **(D)** The organization of the EWMN manuscript page (after Yaniv and Golani, 1987).

*The movement modules may be conceived as whole-body rotations* (*or parts thereof*) *around the main axes of the body. In interactional behavior they are elicited in response to specific relations of opposition* (*including contact*) *with the partner.* A study of honey badger ritualized fighting behavior confirms the difference between free and constrained behavior. It is the difference between the (i) number and (ii) types of spatial modules available to the free animal vs. the constrained animal, during specific stimulus situations between the partners. The heavy and light limbs hierarchy, the whole-body axes of rotation of the animals, and the topographical positions on the partners’ body surface - all the movement material used in the badger study - are represented in a nutshell in Figure 15 and Supplementary Figure 1. While *the behavior of the free partner’s is unpredictable, having all the modules at its disposition given any opposition with the partner,* the constrained partner uses a narrowed repertoire, with the order of narrowing being prescribed by the *bauplan*.

*Stimulus-bound versus free behavior.* Having obtained the stimulus situation impinging on a focal animal (e.g., “animal A’s partner’s snout is nearly touching animal B’s hindquarters”) we document the movement options available to a focal animal. Using this method, we have shown that in near- or full contact interactions, while one of the partners is absolutely stimulus-bound, responding swiftly with one and only one low-mobility module, the other partner is free, responding or not, and when responding, manifesting unpredictably any movement module to any relationship of opposition (Yaniv and Golani, 1987).

*Quantifying stereotypy.* The degree of the momentary narrowing (or build-up) of the animal’s repertoire is quantifiable in terms of the number of spatial modules available to the animal, the range of trunk metameres recruited along the AP axis of the most advanced module exhibited by the animal at the time of measurement, and the predictability of the sequence of primitives and modules performed. Note, that behavior can unfold along multiple modules and yet be completely predictable, or else be constrained within a single module yet exhibit a narrow range of as few as two primitives but be as unpredictable as a random sequence written in binary code. In other words, the predictability criterion is orthogonal to the other two criteria characterizing the animal’s repertoire (Golani et al., 1999).

*Collinearity and modularity in the phylogeny of vertebrate adult forward progression.* The following type of geometric description regards which segment moves on which within the kinematic linkage, importantly attending to the way in which the organism is hooked to the world both biomechanically and perceptually. Incorporating the law of heavy and light limbs hierarchy into the hypothetical evolutionary stages is essential, if only because of the required use of situated natural kind primitives, modules, and generative rules: whether the forequarters move on the hindquarters or vice versa, and whether the forequarters are fixated or not in the absolute frame, are fundamental kinematic features that should be articulated in the evolutionary history of locomotor behavior of organisms, because they are situated in the world, and should be represented as such.

Given the paucity of data on the actualgenesis, and partly also on the ontogenesis of locomotor behavior in the large number of different vertebrate species, we can focus on the geometry of the kinematics of adult forward progression, which is extensively documented in vertebrates. Relating to the *bauplan* established so far, we can say that in the evolution of forward progression, (i) horizontal (lateral) movement of the parts of the trunk (“lateral undulation”) preceded vertical movement (“Dorsi-Ventral undulation”); (ii) a Posterior-to-Anterior (PA) linear heavy-to-light mechanical hierarchy evolved and prevailed first (forequarters move on hindquarters), and an Anterior-to-Posterior (AP) heavy-to-light linear mechanical hierarchy evolved next (hindquarters move on forequarters); and (iii) Proximo-Distal (PD) buildup of movement (from propulsion by trunk to propulsion by appendages; e.g., in Tiktaalik; Shubin et al., 2006) preceded Distal-to-Proximal (DP) narrowing (from propulsion by appendages to propulsion by trunk; e.g., in snakes).

The most primitive form of forward swimming consists in the vertebrate *bauplan* of horizontal (lateral) side-to-side movements that propagate from head to tail. During the side-to-side movements, the trunk metameres (“heavy” in EW notation frame), etc., change in AP order their horizontal direction, generating so-called lateral undulation. In the most primitive version of this form of locomotion the head moves in the horizontal plane from side to side in absolute space, generating waves that oscillate laterally, and propagate linearly backwards, producing the propulsive thrust. The heavy to light limbs hierarchy proceeds from tail (heaviest) to head (lightest). In the most primitive version of this type of swimming, the head crosses during its movement the midsagittal plane of its next caudal neighbor, moving from side to side also in absolute space (its direction is not fixated). This, most primitive version, is exercised in the lamprey, belonging to an ancient extant lineage of jawless fish of the order Petromyzontiformes, superclass Cyclostomata (Supplementary Video 14).

In the next, more derived form of forward progression, the head still performs the same side-to-side movements in body-related frame, but maintains invariant its horizontal direction in absolute space (head fixation), due to equal, simultaneous, neck movements in the opposite direction. At this evolutionary stage the hindquarters still function as the heavy base. The propulsive thrust comes from the body; the heavy to light limbs hierarchy still proceeds from tail to head; the head is the lightest segment in the hierarchy, based on the distribution of support along the body (Supplementary Video 15).

In the next, still more derived phylogenetic stage, the heavy-to-light hierarchy is reversed, as the forequarters and then the whole trunk become the heaviest part of the body, and the hindquarters or even the anal fin alone, moves on the trunk, becoming the lightest segment. This AP confinement of the propulsive lateral movements mirrors the reversal of hierarchy following the shift of weight to the forequarters in actualgenesis in quadrupeds. It is full blown in the more progressive propulsive swimming of recent *tunniform* fishes (Lindsey, 1978; Webb, 1994).

Another evolutionary trend is a proximo-distal transition of active movements from the trunk to the fins (Lindsey, 1978; Webb and Weihs, 1986; Eilam, 1992; Webb, 1994). This is illustrated in the sun fish (*Mola mola*), whose trunk is short, rigid, and does not contribute actively to locomotion, which is generated by the back and anal fins (Supplementary Video 16). Movement of the parts of the trunk in the vertical dimension (forward progression by “dorsi-ventral undulation”) did not evolve in fish.

Limbless amphibians’ (*Apoda*) forward progression during swimming consists of horizontal side-to-side movements that propagate from head to tail, the head crossing the midsagittal plane from side to side. Recent salamanders (*Urodela*) swim with exclusive horizontal movements. They represent the prototype tetrapod and serve as a model for primeval quadruped terrestrial locomotion (Eilam, 1992) in which they use both lateral movements and stepping, also moving their head horizontally from side to side across the midsagittal plain (Chong et al., 2021). Toads and frogs (*Anura*) show a proximo-distal transition of movements from the trunk to their hind legs thereby manifesting an evolutionary reversal in the heavy and light limbs hierarchy.

Horizontal (lateral) undulation in swimming is also manifested in aquatic reptiles (e.g., crocodile, *Crocodylus* sp.), and in a primitive mammal (otter shrew, *Potamogale* sp.). It is also exhibited in terrestrial locomotion in reptiles (e.g., walking crocodile, *Crocodilus* sp.), and mammalian quadrupeds (e.g., ferret, *Mustella* sp.). In this type of locomotion the trunk movements are coupled to diagonal stepping, and forward propulsion is transferred to the appendages, which then serve as the heavy base(s) of support (Kafkafi and Golani, 1998; Gruntman et al., 2007). As locomotion evolves and diversifies, propulsion is transferred to the appendages also in other terrestrial groups (e.g., turtle*, Testudo* sp.; wolf*, Canis lupus*). In the most extreme forms along this line, in so-called cursorial animals (Gambaryan, 1974) (e.g., horse, *Equus* sp.), the four legs produce the propulsive power without movements of the trunk.

Having videotaped free locomotion of ferrets (*Mustella putorius*) from below through a glass floor, and having measured the lateral bending along the head, torso, and tail, and the location of the four paws, and having introduced an algorithm which extracts the phase (and thus also the relative phase) even when the movements were neither periodic nor symmetric, it was shown that relative phases between segments have preferred values, which are relatively independent of the amplitude, duration, and asymmetry of the movement. In particular, both walking and turning can be explained as modulations of a single pattern: an AP traveling wave of horizontal movement with a wavelength of approximately one length of the body. The relative phase between movements of adjacent segments is similar when the body is in S shape (i.e., when walking forward), or C shape (i.e., when turning). The movements of the paws in the horizontal plane can also be considered as part of this traveling wave. These findings suggest that the concept of traveling waves of horizontal bending, as found in the locomotion of undulating fish, can be generalized in two ways: (i) by considering the axis around which the movement is centered, it applies not only to forward locomotion, but also to turning; (ii) by incorporating the position of the paws, it applies also to the movement of quadrupeds (Kafkafi and Golani, 1998).

Along still another line of diversification, forward progression is accomplished by body-related vertical movement. In terrestrial quadrupeds, in adult animals during high speed progression (e.g., gallop in rodents), and in phylogeny, in semi-aquatic mammals like the beaver (*Castor* sp. Supplementary Video 1) and the otter (*Lutra* sp. Supplementary Video 18). Similarly whales, Cetacea, swim using body-related vertical paddling of the horizontally oriented tail along the body-related vertical dimension (Supplementary Video 19).

It has been argued (Dubbeldam, 2001) that uncoupling of central pattern generators for locomotion may have been an important mechanism in evolution allowing the emergence of richer behavioral repertoires and thus of behavioral expansion, including play and play-like behavior.

*Behavioral baupläne in taxa having other anatomical body plans.* Endowing the vertebrate and arthropod body plan with a central role in shaping the behavioral *bauplan*, it would be useful to ask how other, very different body plans could guide the search for the corresponding behavioral *bauplän*e they might induce.

*What is the behavioral bauplan of hydra* (*Cnidaria, Hydrozoa*)*?* The skeleton is a locomotor instrument, and skeletal anatomy is “for behavior” (Coghill, 1929). A hydra’s body plan, for example, is characterized by a single oral-aboral main axis connecting its two poles. Both poles can serve as bases of support, but there is a priority for standing on the aboral pole. Vertical planes centred on the main axis (Supplementary Figure 2) could exhaustively map the hydra’s operational life-space. Is the hydra constrained to move actively along these planes when using one of its two bases of support? Hydras expand and contract radially, and longitudinally along the oral-aboral axis (Han et al., 2018). Is there, following a stressful situation, a characteristic build-up of mobility, whereby the contracted state is used as a “zero position”? Progression is accomplished by alternating between the two bases of support, thus reversing the light and heavy limbs’ hierarchy, by “looping” (inchworming), and/or by “somersaulting” (Han et al., 2018), or by gliding on the aboral base, or by “walking” on the tentacles on the oral base, or by floating. The reported distinction between walking and climbing (Han et al., 2018) appears to describe the same behavior, merely reflecting two orientations of the substrate (Supplementary Video 20).

Is there, however, a hierarchy in the performance of these forms of progression across actual genesis and development? Is there an order in the recruitment of the parts of the hydra’s body during the performance of a vertical movement (e.g., an oral-aboral order of recruitment of body regions?) Are there preferred rotational sides (Eshkol and Harries, 2001, 2004) for whole body vertical plane movement (and the direction of progression in body-related space)? Can parts of the body move actively along horizontal and intermediate planes, which are oriented perpendicularly or diagonally in reference to the vertical planes (Eshkol and Harries, 2001, 2004)? Since the co-alignment of fibers and axis appears to be mediated by mechanical feedback (Livshits et al., 2017; Braun and Keren, 2018), which itself mediates the influence of movement, are anatomical and behavioral morphogenesis related? All this leads us back to a revised version of D’Arcy Thompson’s query (d'Arcy, 1942): Given a symmetrical hydra in symmetrical space, what part of physical space does its life-space occupy at any one time, and is there any overlap between a hydra’s life-space and the life-space of “large and clumsy mathematicians”? Hydras are not expected to share with mathematicians right and left, nor are they expected to rotate around their body-related longitudinal axis in preferred directions, having no distinct rotational topographical sides. However, endowed with a substrate-related vertical, their life-space appears to extend equally along any substrate, whatever its orientation is in physical space. Sharing with mathematicians a distinction between foot-end and oral-end, both hydras and humans manifest a preference for a foot-on-substrate stance, attending with the opposite oral-end, which is also equipped with effectors (mouth, tentacles, hands), toward open space, and using the oral-end as a heavy base only transiently. An abrupt onset of akinesia and its gradual dissipation, and hence a growth and differentiation in life-space, is exhibited in humans afflicted by some forms of Parkinson’s disease (see chapter on Parkinsonian space and time; Sacks, 1991). What, if any, is the actual genesis of mobility in the hydra?

What is the behavioral *bauplan* of living echinoderms? The relationship between body plan and behavioral *bauplan* is similarly intriguing in living echinoderms, involving larvae with bilateral symmetry, animal-vegetal, anterior–posterior, left–right, dorsal-ventral (oral-aboral) axes, which metamorphose into pentameral adults with changed body axes. In this phylum, head, anatomical anterior, and locomotor anterior do not necessarily coexist nor coincide topographically (Crozier, 1920; Grabowsky, 1994; Astley, 2012; Parker et al., 2014; Omori et al., 2018). The take-home message in this phylum is to relate the echinoderms’ body plans to the *actual genesis* scale of behavior *in statu nascendi* situations (in the course of being formed). This implies looking for a natural reference behavior, from which the behavior builds up and to which it narrows, and describing it in a natural frame, using generalizable symbols. Both the demonstration of a “mobility gradient” in echinoderms, or even a demonstration of its absence (implying an absence of regulation of freedom of movement under stress), would be a significant accomplishment.

The geometry of behavior in locale space. While displacement in locale space can be viewed as a “by-product” of articulated movement along the six spatial modular dimensions (the subject of the current essay), there is, on top of the six segmental articulated modules, a locale operational space. The neural correlates of this locale space – the place cells in the hippocampus, and the grid cells in the brain’s medial entorhinal cortex, have been extensively mapped and studied (for seminal contributions in this discipline see O'keefe and Nadel, 1978; Tsao et al., 2018). The place- and grid cells are, however, neural correlates of a kinematic *bauplan* operating at the level of the path traced by the animal in locale space. The kinematic primitives of the locale *bauplan* are progression segments, and staying in place episodes. The modules are excursions performed in reference to an origin, or a home base, established by the organism. Generative rules specify the rate of growth and the ways in which the excursions differentiate in locale space (Golani and Benjamini, 2018) (also see lecture on Supplementary Video 21). The locale *bauplan* illustrates how a morphogenetic bauplan can be established at the level of the path, which is one level above the level that can be portrayed by movement notation. Adopting the locale *bauplan* as a roadmap in the study of the neural correlates of spatial behavior should greatly expand the scope of the discipline studying the neural correlates of spatial behavior (e.g., Savelli and Knierim, 2010, 2019; Rao et al., 2021).

## 4 Discussion

*A bauplan encompassing genes, anatomy, and behavior within the same scientific discipline?* A major problem in the study of life phenomena is how to encompass the various levels of the pyramid of life within the same discipline. The current essay shows how the levels of genes, anatomy, and behavior, as well as the physical environment in which the organism is situated, share common geometrical features. This is accomplished by showing that features of the anatomical body plan on the one hand, and features of the physical dimensions of the environment on the other hand, share their topology with the geometry of movement.

A powerful methodological principle of evo-devo is its attention to simple anatomical facts that have been taken for granted for several decades. The skeletons of some animal phyla are constructed of rigid articulated segments (primitives); assembled into organs (modules); have a head-to-tail axis and a serial modular structure that grows and differentiates in a linear order, etc. Asking what were the genetic and molecular processes that mediated the body plan would not have been possible without first realizing that there *was* a body plan. In a similar way, the current essay defines: (*i*) the primitive of locomotor behavior: a discrete continuous change of angular relation between a trunk segment and its next “lighter” neighbor, along a specific modular spatial dimension; (*ii*) the six spatial modular dimensions: horizontal, forward, etc. (Figure 1B); and (*iii*) the generative rules of build-up and narrowing of the animal’s locomotor repertoire in actualgenesis and in ontogenesis: modular collinearity of recruitment and reversed linearity in narrowing. The animal’s behavioral *bauplan* is induced by the animal’s body plan, increasing the likelihood of the *bauplan*’s generalizability. Not unlike evo-devo, which has integrated several seemingly disparate fields into a single discipline (Tickle and Urrutia, 2017), a *bauplan* which mirrors the anatomical constraints of the body plan is more likely to encompass genes, anatomy, and behavior within the same scientific discipline.

The incorporation of the study of the moment-by-moment morphogenesis of movement, which equally consists in the case of movement of build-up and narrowing processes, adds a whole new perspective to the study of the (homological) *bauplan* of behavior. It is hard to understand why the same study of morphogenesis, which has proved so suggestive in comparative anatomy, has not been, and is not being pursued more vigorously by researchers of movement-based behavior (It is being pursued, however, in the study of birdsong; Lipkind et al., 2017), where a morphogenetic approach, molded in the study of movement-based behavior (Tchernichovski et al., 1998; Lipkind et al., 2004) has been commendably developed for the study of vocal learning).

*A study of the morphogenetic buildup and narrowing of behavior is essential for portraying the bauplan of behavior, and adds a viewpoint on anatomical morphogenesis*. The problem of what the flow of movement-based behavior consists of has occupied students of animal behavior since Darwin (1872). The ethological and current computational phenotyping view of behavior is conceived as a sequence of fixed, or less fixed, or variable, units. In sheer contrast, the morphogenetic description of locomotor, exploratory, and social behavior (agonistic, playful, courtship), in development, in recovery, under stress, and with drugs, consists of buildup and narrowing, channeled separately within six or seven modules that unfold sequentially, sometimes partly overlapping, and at other times following each other in a prescribed order, across development, unfolding and enfolding incessantly in actualgenesis. The process involves neurological enabling and constraining of kinematic freedom, as well as a decrease or increase in stimulus boundedness. While similar processes presumably take place at other levels of the pyramid of life, the kinematic processes are fortunately fully observable, allowing a full mapping of the geometric manifold. Since kinematic invariance discloses management of perception and attention, the description is also phenomenological (see section on *umwelt* below).

*Whole body rotations around the longitudinal axis of the body* (*righting*; *logrolling*). In EWMN, “rotatory” movement is a movement of a segment around its own longitudinal axis (Figure 5B, left). Whole-body rotations around the longitudinal axis of the trunk, such as those performed by infants from supine to prone on a substrate (Trojaniello et al., 2014; Gao and Calderon, 2020), or in the air during a fall (Pellis and Pellis, 1994), or from prone to supine in infants lying under their dam (Eilam and Smotherman, 1998), are all performed in an AP order, starting in the recruitment of the infant’s eyes and proceeding to recruit the head, then the neck, then the chest and then the torso. For simplicity they were not reported in the current essay to be performed during the early stage of the mobility sequence (Figure 1B), because the infant’s or lesioned animal’s posture already implies active maintenance of proneness, be it during development or recovery. The righting response merely buffers out a perturbation of the prone reference posture, and the transition to a supine posture reflects an adjustment for suckling following a stimulation by the mother’s nipple: both transitions do not imply build-up nor narrowing. The same whole-body rotations are reported in the infant or adult animal’s *bauplan* when performed during the exuberant portion, reflecting a build-up in actualgenesis and ontogenesis. Righting from supine to prone in human infants is, however, the first build-up module in actualgenesis and ontogenesis in the *altricial* human neonate.

*A Hox gene that mediates whole body rotations around the longitudinal axis of the body may indicate how tool kit genes shape other modules belonging to the behavioral plan.* The problem of how genes shape behavior requires a behavioral *bauplan* that can be used as a roadmap for such search. The behavioral studies reviewed in the current work focused on morphogenesis, used “monsters” to highlight the core plan, and established the same *bauplan* in intact organisms across a wide range of taxa and situations, all concurrently and independently of evo-devo. Given the large body of data demonstrating how toolkit genes shape the anatomical body plan (Gilbert, 1997; Wagner, 2007; Carroll, 2008), the locomotor *bauplan* might now be used as a roadmap, guiding the search for how toolkit genes shape this *bauplan*. A promising line of studies reveals that post-developmental downregulation of the Hox gene Ultrabithorax (Ubx) in adult neurons of *Drosophila* leads to substantial anomalies in flight, suggesting that the Hox genes are a component of the genetic program that maintains normal neural function in adult *Drosophila* (Raouf Issa et al., 2022). A series of studies also suggests that a common miRNA-Hox genetic module manifests its behavioral roles via repression of a specific Hox gene (Picao-Osorio et al., 2015, 2017; Issa et al., 2019). This module can be re-deployed in different neurons to control functionally equivalent whole-body rotation movements around the longitudinal axis of the body (self-righting; surface-righting; logrolling) in biomechanically distinct organisms such as fruit fly larvae and adult flies. Using our locomotor *bauplan* as a road map, we suggest that a corresponding novel functional role of the Hox genes should be sought in both arthropods and vertebrates for modular locomotor dimensions belonging to the mobility gradient *bauplan*, such as the rotation around the vertical axis located in the hindquarters (Figures 15 and Supplementary Figure 1), the rotation around the vertical axis located in the forequarters (Figure 15 and Supplementary Figure 1), and the rotation around the horizontal side-to-side body axis (Figure 15), all of which manifest a collinear order of recruitment along the AP axis, in tandem with the Hox order of expression.

*The modular geometry of life-space mirrors the geometry of physical space.* A major question addressed in the current essay is whether and how is the geometry of physical space mirrored (enfolded; re-unfolded) in the geometry of behavior? Does the animals’ movement reflect at all an understanding of spatial dimensionality? How many and what physical dimensions are mirrored in movement-based behavior? Is the affordance, i.e., the operational accessibility, of the respective physical dimensions reflected in the kinematics of the trunk linkage? And more generally, how is situatedness in the environment concretely reflected in movement?

Having at hand the EWMN General System of Reference (SOR), and the law of heavy and light limbs, we first demonstrated that the primitives of movement are relatively pure horizontal and vertical movements, *not* diagonal movements along intermediate planes. In other words, horizontal and vertical movements are *bona fide* horizontal and vertical, not mere projections on the horizontal and vertical dimensions of variously oriented movements (Figures 1, 8). Next we demonstrated that during actualgenesis there is a separate bookkeeping for the progressive linear order of recruitment of the parts of the trunk: the primitives belonging to a specific module are always recruited in the same head-to-tail order, and are performed in module-specific bouts. The generative rule applying to the build-up in actualgenesis is: a movement of a part of the trunk is not performed along a modular dimension unless the part of the trunk anterior to it has already moved along that modular dimension, and unless that part has already moved in the current sequence along the previously-prescribed modular dimension (horizontal, then forward, then vertical movement culminating with tumbling pelvis-on, then vertical culminating with tumbling head on, first with, and then without snout contact, etc.). Starting from extended immobility, every time an animal commences to move, it enacts a dynamic life-space, building it up and/or narrowing it. On the one hand, this life-space reflects physical space, the horizontal locomotor module reflecting the physical horizontal dimension, etc.; but on the other hand, it is much richer, impregnating the space with the functional significances, or so-called affordances, identified by the organism (von Uexküll, 1957; Gibson, 2014). The physical dimensional discontinuity characterizing the outside world is enfolded in the recruitment of the parts of the trunk along each module separately. For example, a rat finding itself in a novel environment first establishes a well-trodden (i.e., familiarized) horizontal plane, not unlike Uexkull’s well-trodden path (von Uexküll, 1957), then enacts horizontal directions on it by un-arching and then progressing forward (Figure 9), and then performs increasingly larger vertical planes. These directions and planes are not only explored and attended by the rat, they are also *enabled* by it neurologically: the very essence of the “warm-up” phenomenon involves a systematic dissipation of the rat’s own partial *akinesia* (Golani et al., 1979); moving along a dimension with a body part induces an enabling process (Sinnamon and Galer, 1984; Sinnamon et al., 1987; Chevalier and Deniau, 1990; Tresch et al., 1995; Sinnamon et al., 2000), facilitating the next movement along that dimension, and potentiating movement along the next dimension and/or the next trunk part (see below section on the cerebral organization of the mobility gradient). The rat probes repeatedly into novel directions and planes, cognizing and re-cognizing them, and “digging” its way both along the body and in neural and physical space, by impregnating it with novel affordances of mobility (see also Cohen et al., 2015). The impregnated meaning consists in (*i*) a cognitive content manifested in the opening or closing of geometric dimensions, and thus disclosing the rat’s assessment of the current affordances of movement within these dimensions and along these directions (the prospective growth and/or narrowing of life-space is embodied in the kinematic freedom of the trunk linkage); and (*ii*) an emotional content, manifesting and communicating how inviting or threatening (Schneirla, 1959) these dimensions and directions appear to the animal, the so-called *befindlichkeit* (Heidegger, 1962) of the organism – “the state in which it is found” (Dreyfus, 2012). Since this state has the same homologous, and therefore invariant, kinematic shape in all the members of the phylum, it is also understood directly by congeners, by organisms belonging to the same phylum (including zoologists), and first and foremost by the animal itself (Darwin, 1872; Cheng, 1986; Yigael, 2018; Damasio, 2021).

*Contextual and pharmacological modification of infant warm-up*. The dissipation-of-immobility function of warm-up is nicely demonstrated by the following observation: when rats eat food pellets their hindlegs are rooted to the ground. After consuming the food and before leaving the feeding place, they perform a warm-up sequence of duration correlates with the time interval spent with rooted hindlegs, presumably reflecting the amount of warm-up movement necessary for dissipating the immobility induced during eating. This warm-up duration can be extended by offering the rats either larger, or harder-to-chew food pellets (Whishaw and Gorny, 1991).

Maternal nicotine exposure prior to conception influences 10-day-old rat pup motor behavior in an open field task by inducing a stereotypy in their “warm-up” sequences: once making one lateral head movement they then repeat the movement at regular intervals rather than escalating warm-up movements into locomotion (Torabi et al., 2021).

*Behavior enfolds* (*mirrors*) *the continuity within- and discontinuity across physical spatial dimensions.* Does the animal distinguish between continuity within and discontinuity across physical dimensions? The built-in distinction between horizontal, forward and vertical modules in behavior shows that the animals distinguish between the respective physical dimensions. As soon as they perform a movement along a new physical dimension, they treat it as belonging to a new domain, commencing to build it up along a separate module.

The currently investigated *bauplan* thus also addresses the question pondered by Thompson in his letter to Whitehead, portraying the experienced dimensionality of the organism - its dynamic geometric mode of being-in-the-world (Heidegger, 1962). The discontinuity across dimensions in life-space mirrors (re-unfolds) the physical discontinuity across dimensions scanned outside. Physical space defines what is possible for the organism in the world at the scales of phylogeny, ontogeny, and actual genesis, whereas life-space defines what is actually being manifested and experienced.

*Coordinating the geometry of life-space with the affordances of the animate environment*. Traditionally, the behavior exhibited during animate encounters has been interpreted as a ritualized transfer of information from a sender to a receiver. Common examples are cowering as a signal communicating submission to a potentially stronger congener (e.g., in wolves, Golani and Moran, 1983; Schenkel, 1967; Lorenz, 1974; Supplementary Figure 3A; Supplementary Video 22); stotting or pronging as a signal informing a coursing predator about an antelope’s ability to outrun it (Fitzgibbon and Fanshawe, 1988; Supplementary Figure 3C; Springbok pronking in Supplementary Video 23); courtship as an invitation for copulation; or even a “dance” claimed to mesmerize a potential prey as preparation for killing it without a struggle (stoat “dancing” vis-à-vis a rabbit before killing it; see below). In these examples the function of the display is assumed to be the transfer of information from one animal (sender) to another (receiver), affecting the behavior of the receiver.

*Functional analysis should follow structural analysis.* In the information transfer scheme above, however, what would be immediately striking to a comparative anatomist, a geneticists or a developmental biologist, is the common absence of any realization that social behavior, much like any other biological entity, requires a systematic structural analysis that would precede any hypothesis regarding its function (Lewontin, 1979). The absence or paucity of structural analysis of movement-based behavior characterizes the work of sociobiologists and behavioral ecologists (e.g., Wilson, 2000), of leaders in biosemiotics (e.g., Sebeok, 1977; Barbieri, 2009), and some ethologists (e.g., Dawkins, 2016). It would be inconceivable to discuss the function of, e.g., the kidney or the hippocampus, without first describing their respective anatomy, histology, the natural sutures between them and the rest of the body, their connectivity, as well as their existence as relatively independent modules (Wagner, 2000). In a similar way, it should be scientifically unacceptable to discuss the function of behavioral categories established on the basis of *ad hoc* claims for “expert knowledge.” Colin Beer, a second-generation founder of ethology described “expert knowledge” regarding movement-based behavior as belonging to “the school of immaculate perception” (Beer, 1974).

Signifying the meaning of the behaviors illustrated in Supplementary Figure 3A,B, as “submission” or “inferiority” is therefore insufficient, compared to the explanatory wealth offered by a detailed morphogenetic literate description. As noted by Goethe, it is “useless to attempt to express the nature of a thing abstractedly. Effects we can perceive, and a complete history of those effects would in fact sufficiently define the nature of the thing itself. We should try in vain to describe a man’s character, but let his acts be collected and an idea of the character will be presented to us” (von Goethe, 1996). Suspending such initial labeling we detail below a description of such a behavior, including its animate context, its actual-, onto-, and presumed phylogenetic history, and the detailed kinematic combative actions it both affords and assesses. The signifier in Supplementary Figures 3A, B, is the momentary freedom of movement exercised by the wolf’s skeletal linkage. It signifies quantitatively how stressful the momentary situation is for the wolf, and it signifies the affordances of the situation to the rival, as well as to a human observer. The founders of the ethological theory missed Goethe’s “complete history of this event.” Once such history is provided, including the morphogenesis, context, generalizability, location within the geometric manifold, etc., there is no harm in also referring to the behavior using a shorthand label.

Viewing the priority of structural analysis as imperative we refrain henceforth from relating to adaptive hypotheses that are not preceded by, and grounded in, a systematic structural analysis of the morphogenesis of behavior. Any functional hypothesis relating to movement material belonging to the mobility *bauplan* should relate to its primitives, modules, generative rules, and situatedness (for a commendable example on how comparative anatomists systematically proceed from a structural analysis to each and every functional claim, see Jones et al., 2018; Jones et al., 2021).

Depending on the animate partner’s identity, distance and opposition, morphogenesis may yield narrowed and stimulus-bound versions (e.g., “inferior” wolf, badger, or rat ritualized fighting; Supplementary Figures 3A,B), or else built up, large amplitude, differentiated, exuberant movements. In *Tetrapoda*, the exuberant versions may involve simultaneous release of all feet contact with ground (as in stotting in antelopes, Supplementary Figure 3C), logrolling and tumbling in the air (as in stoats “war” dance, see below), or movement within previously untraversed planes (as in bird courtship, see section on birds-of-paradise below).

Notably, much like any other movement type constituting a stage in the morphogenetic buildup sequence of the *bauplan* (Figure 1), the performance of a novel movement type (*i*) foreshadows the repeated performance of that same novel movement type, (*ii*) enables the performance of still unforeseen, more advanced types, and (*iii*) liberates, presumably via proprioceptive activation, the organism from being controlled (and distracted) by both external and internal stimuli, endowing it with an increasing freedom to act or not to act, on the basis of its internal context. The performance of a movement is importantly communicated to the behaving animal itself, informing it about its own level of mobility and stimulus boundedness upon facing a significant impending situation.

In a seminal study illustrating a display’s critical function of self-communication, Cheng (1986) showed that during nest-building activity, the cooing of female barbary doves (*Streptopelia risoria*) stimulates the growth of their own ovaries, enhancing follicular growth. A bonded, deafened, female dove coos while nest-building, but when information about the cooing (and hence about the affordance of incubation and breeding) does not reach her auditory system, the follicles do not grow. The cooing, part of the female’s nesting courtship, is first and foremost necessary for cultivating her own follicles. Similarly, the performance of a specific movement type in the mobility buildup sequence is required for enabling the performance of the next movement type in the prescribed *bauplan*.

*Narrowing the freedom of movement and increasing stimulus boundedness simplifies control.* A homology obtains between the narrowing of behavior exhibited by the constrained wolves in Supplementary Figure 3, by infant tetrapods in a presumably stressful environment, by dopaminergic drug-induced akinesia in rats (as with apomorphine, Figure 12A), and apparently also by the akinesia characterizing human Parkinson’s disease (Cools, 1992; Ikeda et al., 2013). The adaptive function of narrowing can be appreciated in intact wolf interactional agonistic behavior: the redundancy of degrees of freedom endowed by a linkage of serially-connected rigid spinal segments poses severe problems of intersegmental coordination for the organism. A main mechanism for coping with the excessive freedom is locking of some of the joints so as to reduce the problem of redundancy of degrees of motor control (Bernsteĭn, 1967). The current essay offers a specification of the phyletic architecture regulating the freedom of movement of the trunk linkage. Regulation is accomplished by an ordered linear unlocking and locking of joints along the linkage. Unlocking enacts, and locking eradicates freedom within and across the specified spatial modules, in accordance with the degree of stress of the situation. The control problem is also simplified by a stimulus bound animal using a limited set of stereotyped predictable “martial arts” blocking techniques.

*Building up the freedom of movement of the trunk linkage, and liberating the organism from stimulus boundedness, enacts a swift, unpredictable, free organism.* Coming out of their den, stoats (*Mustela ermine*) perform the whole warm-up array, starting with side-to-side horizontal head movements, and commencing with whole body horizontal pivoting around the hind legs, then forward, then vertical, and then tumbling head-on, stotting, tumbling head-on, and log-rolling in the air, in an unpredictable, lightning-swift strikes, all the while performing increasingly longer and more tortuous excursions from the den into the environment (personal observation). The entire sequence is paradigmatic because (*i*) it provides a live, embodied, manifestation, all the way from the first horizontal head movement to exuberant behavior, all in one behavioral bout, of the homology that has been described in the current review in a piecemeal fashion, using examples taken from different animals in various situations, (*ii*) it also provides a manifestation of a second homology, of behavior in locale space (Wexler et al., 2018; Golani and Benjamini, 2018; Supplementary Video 21), and (*iii*) the exuberant part is typically performed face-to-face with a prospective docile prey victim (e.g., a rabbit), making it difficult to come up with a credible social communicative adaptive story (why, on earth, would a carnivore perform a “war” dance vis-à-vis a “steak” before chewing on it?).

A recent long shot video (Supplementary Video 24), unfortunately not including the most initial warm-up, supports this description. It consists of four increasingly longer and progressively more tortuous excursions, all performed in reference to the same origin. The stoat performs a short excursion consisting of a single outbound and a single inbound portion, including whole-body pivoting around the hindquarters, then proceeds with a second excursion starting with a large vertical rearing episode followed by a frenzy of Protean zigzagging, spinning, looping, and bouncing. This Protean behavior continues in two additional excursions, each consisting of several outbound and inbound portions. The “dance” is performed vis-a-vis a heap of dry leaves, perhaps suspected (or projected) as potential prey.

In other instances, the most differentiated, lightning fast, “exuberant” modules of the buildup sequence are performed during solo behavior (Supplementary Video 25), and in still other instances, while facing a stationary rabbit, the stoat logrolls, bounces, stotts, and performs Protean zigzagging and bouncing that serve as an introduction for a precise killing bite delivered to the back of the neck of a prey, sometime 10 times bigger than the stoat.

The stoat’s flamboyant, extravagant, and exuberant behavior vis-à-vis its docile prey presents a challenge: why would a carnivore dance in front of a docile prey before attacking it? “Death’ or “war” dances, ascribed to human warriors as a way of emotional and spiritual preparation for battle have hardly ever been ascribed by ethologists to animals. The theoretical embarrassment is indeed evident in King Powell’s “The natural history of weasels and stoats” (King and Powell, 2006): “Opinions are divided on whether “dancing” weasels are merely playing, or deliberately using the “dance” as a hunting technique. In favor of the first interpretation is the fact that these “dances” are not confined to situations offering a potential hunting opportunity… some dances are performed without any audience at all (Pounds, 1981). In favor of the second interpretation is the fact that weasels are intelligent…hunters, and if they find themselves surrounded by curious rabbits… they will take the chance to catch one if they can. If they realize the connection between their behavior and the subsequent kill, they might well learn to “dance” on purpose. A third explanation is that the “dances” are an involuntary response to the intense irritation caused by parasitic worms lodged inside the skull… Whatever the interpretation of … “mesmerizing,” …these behaviors… could eventually be reinforced, whether it was deliberate or not.” Upon presenting the same behavior, Attenborough similarly notes that experts generally agree that the stoats do use the manic “infamous death dance to disguise an attack, hypnotizing the prey so as to allow the stoat to get close enough to strike, but they do not suck the blood of the victim vampire-like as folklore would have us believe.”

In sharp contrast to the disciplines prioritizing the social function of communication, in the frame of the current, morphogenetic *bauplan*, it would only be consistent - indeed inevitable - to expect the stoat’s exuberant behavior to be endowed with the same enabling and liberating properties characterizing all the movements of the *bauplan*: recognizing the affordance of a precision kill, the stoat builds up its own mobility, enabling novel movements, increasing repertoire size, liberating itself from the distractive interference of external and internal stimuli, enhancing its own speed, and when ready delivering “the final strike with deadly accuracy… the killed animals often show no injury besides the two pairs of needle-like punctures in the neck” (King and Powell, 2006). The “dance” is indeed a “war” dance, preparing the animal itself for the consummatory kill.

But the consummatory kill, although a significant attractor, is not the sole attractor to which the “war” dance converges: as illustrated in two of the video clips presented above, the “war” dance is performed frequently in solo, in the absence of prey (and hence in the absence of an affordance to kill; Pounds, 1981), and it is also performed frequently between young siblings that appear to be role playing, “taking it in turns to act the passive victim and the demonic dancer of legends” (Supplementary Video 26).

In other words, the “war” dance is an attractor in and of itself, as is the whole mobility *bauplan*, as is the morphogenesis of any behavioral homology (Golani, 1981), and as is the morphogenesis of any anatomical homology (see below the section on why Perceptual Control Theory (PCT, Powers, 1973) is not enough in zoology). The demonstrations that the very performance of so-called “appetitive behavior” is most often a goal in its own right (in the language of PCT - the reference signal) rather than a means to an end, and that a consummatory act may be performed without the appetitive behavior usually preceding it, or that a consummatory act may be performed for the opportunity to perform the appetitive stage, is of the essence in ethology: canaries in confinement build a nest “in order” to collect hours of flying with nesting material; gulls perform full bathing and wading behavior on dry land, ducks perform water-filtration movements in the absence of water; and starlings perform insect prey-catching behavior in the absence of insects (Lorenz, 1981). More generally, “reference signal” (goal) and “perceptual input” (means), keep changing roles across, and even within, performances (Golani and Fentress, 1985).

The “war dance,” therefore, both appears to warm-up the stoat for the culminating consummatory bite, *but* is also attractive in its own right, much like animal and human courting and sexual foreplay, which, except for building up and liberating the partners, is *also* attractive in its own right. The stoat’s war dance thus illustrates the rule rather than the exception.

*The build-up of so-called innate behavior*. More broadly, a gradual build-up is a general feature of species-specific so-called innate behavior at various time scales (Lorenz, 1981): when extending over weeks, (e.g., transition from crawling to walking in human infants) it is described as growth. When extending over days (e.g., nest-building behavior in gulls, whereby a gull, across days, first only touches nesting material, then picks and immediately drops it, then drops it after carrying it for increasing distances, then drops it at the prospective nesting site) it is described as maturation of nest-building behavior. When extending over minutes or hours (e.g., breaking of ostrich eggs behavior by Egyptian vultures) (*Neophron percnopterus*), whereby, over minutes and sometimes over days (personal observation) the bird throws a stone on the egg only after first touching the egg, then walking to the stone and touching it, then picking the stone and immediately dropping it, then picking and throwing the stone, multiple times, all over the place, then standing over the egg with the stone, aiming, throwing and missing, and only after many failures, actually hitting the egg, breaking it, and eating its content. The build-up may be enhanced following many such encounters, but is hardly ever eliminated. For an abbreviated version of this behavior (see Supplementary Video 27).

A similar build-up is exhibited by honey badgers (*Melivora capensis*), whereby to reach a morsel of food hanging on a tree, the badger, over many minutes, and typically over half an hour, rears toward it, and not having in its repertoire jumps, looks around for large-enough objects such as logs of wood, alternates between climbing on them, and pushing them around haphazardly, until the log (*i*) happens to be located right under the hanging morsel (*ii*) in a position that allows the badger to reach the morsel after (*iii*) climbing the object. Since all 3 conditions are rarely fulfilled simultaneously (the badger climbs the log in the wrong locations, and misses a climb when the log is right under the morsel), this type of behavior may go on over extended durations, nevertheless always culminating with a success. Having demonstrated this build-up sequence for many years to classes of students in my “introduction to ethology” course, on the same individual, who narrowed the sequence only slightly over the years, I used to contrast it with “the straightway,” recounted by Wolfgang Kohler in his insight theory (Köhler, 1921): when presented with a cluster of bananas hanging from the ceiling of a cage plus a series of sticks that could be assembled into a single long stick with a hook at the end, a chimp examined visually the bananas and the sticks, stood up, assembled the sticks into a long hook, and then used the hook to get the banana cluster, all in a single move.

*Key managed kinematic quantities and the organism’s umwelt.* Having started this essay with a continuous recording of the Cartesian coordinates of all the joints of a vertebrate’s or an arthropod’s skeletal linkage, now possible using DeepLabCut (Mathis et al., 2018); and having followed with a subsequent computation of the spatial orientation of all the rigid parts of the skeleton in absolute space, in reference to their respective next heavier neighbor, including the orientation of the body parts that serve as the base of support, the next obvious question would be: *what is relevantly the matter in the recorded behavior?* One way to answer this question is to identify the relatively stable recorded variable(s), and examine whether the other, unstable variables are enslaved so as to maintain the stability of the relatively stable ones. In *synergetics* the relatively stable variable is the so-called *order parameter* (Haken, 1977); in Perceptual Control Theory (PCT; Powers, 1973) it is the *controlled or key variable*, or the *controlled quantity*; and in our own work we describe “*routes of convergence*,” isolating kinematic transients that converge to relatively stable, homeostatically maintained, kinematic values, or *attractors* (Golani, 1976; Golani et al., 1981).

In the current section we establish the trajectory traced by the head in absolute space as a candidate key variable, or order parameter, to which the animal’s trunk and appendages kinematic linkage movements are enslaved. But in the section that follows we strongly qualify this statement, adding a zoological perspective. In physics and in control theory, a description of the variables that maintain the stability of the key variable(s) by their fluctuations is often regarded as a “black box.” To understand what is relevantly the matter while driving a car on a road, I have to attend to, and manage, the car’s distance from the side-walk’s edge, the magnitude of the empty space extending in front of the car, and the speed of the optic array’s flow backwards, but *not* to the detail of my hands’ rotations of the wheel. In contrast, a description of the perceptual input generated by the kinematics which are enslaved to maintain the driving controlled quantities (distance from side-walk, length of empty space ahead, and speed of optic flow) are of the essence in the current essay on locomotor behavior. This is because such description portrays the constraints that shape the behavior, and, as detailed above, because in behavior, “reference signal” (goal) and “perceptual input” (means), keep changing roles across, and even within, performances (Golani and Fentress, 1985): it is as though in zoology, managing the car’s driving variables could be used so as to maintain a specific wheel rotations pattern.

The head, possessing the mechano- and tele receptors, and sometimes also the animal’s most effective weapons, is the “lightest” segment along the trunk’s linkage when the animal is on its legs. The movements of the parts of the linkage sum up to carry the head’s perceptual organs, as well as teeth or horns, along those trajectories and vantage points, which afford effective perception and action. The head kinematics discloses, therefore, the organism’s management of perception, attention and action. The head’s trajectory is a key variable, or an order parameter (Haken, 1977). Affording priority to the input it provides (Golani and Fentress, 1985), the head’s trajectory engages the entire linkage to support specific perceptual references. One example of this is the circular trajectory traced by the infant rat’s snout on the substrate (Figure 9), (Eilam and Golani, 1988). This is a manifestation of part of the rat’s spatial life-space. It reveals the rat’s attention to tactile and/or olfactory input in its immediate vicinity (Figure 9). Another example is that of the steady maintenance of a particular relationship of opposition (near contact) of, e.g., a Tasmanian devil’s (*Sarcophylus harisii*) snout *vis-à-vis* the cheek of its partner during ritualized fighting interactions (Supplementary Figure 4, animal in front and left, frames 005–217). It is a manifestation of invariance in the Tasmanian devil’s interactional life-space (maintaining a stable visual image of the partner’s shoulder or cheek, and all the while opposing the partner’s cheek with its teeth, appears to serve as a “martial arts” blocking technique). The head’s paths and point attractors have *a priori*ty for the respective animals, engaging the trunk so as to maintain an invariance by wriggling, twisting, and even relinquishing feet contact with the ground (Golani, 1976, 1992a; Moran et al., 1981; Golani and Moran, 1983). The snout-of-the-constrained-animal-to-shoulder-or-cheek-of-free-animal opposition is an attractor in interactional space. Having spotted a stronger rival, the wolf in Supplementary Figure 3B is in the middle of a transient leading to the steady maintenance of this attractor.

Kinematic invariants in behavior disclose perceptual quantities controlled by the organism (Powers, 1973; Moran et al., 1981; Mansell, 2020). The unfolding morphogenetic sequence of modular dimensions thus discloses a hierarchy of attention. The precedence of horizontal movement indicates in rats a priority for tactile and olfactory probing of the animal’s vicinity, manifested in repetitive palpation (Knutsen et al., 2006), accompanied by touch with their snout (Welker, 1964); forward progression involving loose snout contact with the substrate reveals tactile and olfactory probing, reaching increasingly distant terrain in developing infants (Eilam and Golani, 1988); vertical movement involving snout contact reveals tactile examination of vertical surfaces, while vertical movement in the air facing open spaces discloses visual and olfactory examination of increasingly more distant horizons. A recovering LH rat first rears in front of nearby objects, and only then rears facing increasingly distant objects (Golani et al., 1979), thus disclosing the progressive expansion of its life-space. Conversely, amphetamine treated rats exhibiting modular linearity in reverse (Adani et al., 1991) first rear while facing distant horizons, and then attend to increasingly closer portions of the environment, ultimately focusing on their own body (Mueller et al., 1982). A similar shrinking of attended space has been reported in monkeys (Nielsen et al., 1983), and in humans experiencing an amphetamine-induced psychosis: starting with attention to far horizons and reporting feelings of spiritual connectedness, human subjects exhibit a severe reduction of attended space, to the point where they investigate portions of their own body in increasing detail (Ellinwood, 1967).

The morphogenetic growth and decay in the extent and number of degrees of freedom available to the organism thus also portray the build-up and narrowing of the organism’s life-space, its *umwelt* (von Uexküll, 1957). This *umwelt* is hierarchical and modular, translating the level of stress experienced by the organism to the level of how unpredictable (versatile, free, Benjamini et al., 2011) the behavior is, and to the level of how extended the domain of its attention is. Modular collinearity thus ties the genes and anatomy levels to the sentience and cognition that characterize life. Sensation (tactile, olfactory, visual), perception (near, far), cognition and attention (horizontal, vertical, near, far) are embodied and embedded in a modular, collinear fashion. The sentient aspects of the organism are shaped by its *bauplan* via its body plan. Genes, body, brain, movement, perception, and attention share, at least during early morphogenesis, the same modular, collinear hierarchically-embedded organization. The situatedness of sentience is an extension of the body plan and of the modular organization of movement. When, during scanning, a novel environmental feature or perturbation is encountered, the organism’s freedom of movement is adjusted in order to cope with the affordances offered by the situation (von Uexküll, 1957; Gibson, 2014).

*Behavior is the control of perception under severe phylogenetic and species-specific constraints.* The Tasmanian devil situated in the front of the top three rows, and in the back in the bottom rows in Supplementary Figure 4, maintains a snout-to-cheek opposition with its partner. From the vantage point of PCT (Powers, 1973; Tariq et al., 2018; Mansell, 2020) the variable trunk movements that support this invariance can be regarded as a black box, so as long as they work they are largely inconsequential and uninteresting. The only aspect relevant to a PCT researcher would be that of the steadily maintained perceptual relation to the partner’s cheek. The PCT methodology would focus on the final product, and assume that it can be directly accomplished in a straightforward way. In sharp contrast, for a zoologist studying the devil’s snout-to-cheek opposition, a study of the kinematics of the trunk linkage accomplishing and supporting the head opposition is of the essence, revealing the generative rules of the mobility gradient *bauplan*. Having been shaped by evolution, the supporting kinematics reflect the cumulative effects of a variety of historical contingencies, reconfiguring and incorporating existing systems into novel ones (Jacob’s tinkering metaphor; Jacob, 1977), and exhibiting a multiplicity of built-in constraints, which endow the behavior with its phyletic (character identity) and species-specific (character state, Wagner, 2000) shape.

A PCT engineer studying the behavior of an infant rat would, for example, might expect the animal to be able to perform a direct transition between any two postures supporting the same or a different reference posture or trajectory of the head. An essential point of the current essay, however, is that during some stages of actualgenesis the infant would have to go through a whole set of movements, abiding by the mobility gradient generative rules, in order to accomplish the necessary build-up for performing a seemingly straightforward specific transition. Furthermore, note that the constraints confronting the animal during movement are “stubborn” ones, in the sense that they cannot be foreseen *a priori*, but must be discovered by observation. Hence, the use of observation is an imperative in a zoological study of behavior.

*On the cerebral organization of the mobility gradient.* The involvement of the basal ganglia in enabling and/or inhibiting the movements of the mobility gradient has been reviewed by us in the past (Golani, 1992a). The distinction between stimulus-bound and free animals, portrayed in the current essay, has been used by Cools and associates as a roadmap for establishing the neurochemical substrate underlying the difference between individual animals that tend to respond on the basis of (narrow, restricted) external context, and animals that tend to respond on the basis of (built up, rich) internal context (Cools, 1992; Ikeda et al., 2013). Using the mobility gradient as a search image, they manipulated dopamine levels in the striatum and in its output stations, showing that the buildup (recruitment) of the various striatal circuits proceeds via spiraling striatal–nigro–striatal connections involving a neurochemical feed-forward loop. It is characterized by region-specific changes in dopamine efflux in serially connected striatal regions, occurring during ontogeny and during exploratory, agonistic and stress-induced behavior. The most advanced stage of the warm-up, characterized by the highest degree of unpredictability and non-stimulus-boundedness only appears when the dorsal striatum is fully activated. Dopamine neuron activity before action initiation gates and invigorates future movements (da Silva et al., 2018). The dorsal striatum frees the animal from external constraints, allowing it to switch behavior unpredictably (Cools, 1981; Cools et al., 1990). In view of the recruitment of the olfactory tubercle, the ventral striatum, and the dorsal striatum during the buildup, the function of the dorsal striatum is superior to that of the ventral striatum, which allows the animal to switch behavior with the help of cues - external stimuli that are originally neutral and irrelevant (for review see Cools et al., 1990). Neural mechanisms of narrowing of the various striatal circuits occurs during progressive pathology and aging (Cools, 1992; Ikeda et al., 2013). Importantly, the Cools, Ikeda, and associates study shows that the cerebral programming processes of cognition, of emotion and of locomotor behavior, all mediated via striatal circuits, are intimately coupled to each other.

*An inside, first-person view of an unpredictable non-stimulus bound mind.* The homology obtaining between the cerebral organization underlying human Parkinson’s disease and that underlying vertebrate animals’ mobility *bauplan* (e.g., Ikeda et al., 2013) supports the translational relevance of conclusions reached in the human domain to conclusions reached in vertebrate animals, and vice versa. A first-person human account of the experience accompanying a buildup in mobility should therefore be translationally relevant for the corresponding experience of buildup in vertebrate animals, including, for example, stoats.

The experience accompanying the transition from a narrow to a built-up view of reality, caused by the efflux of dopamine into a dopamine-deficient brain, has been reported by a sufferer of Parkinson’s Disease treated with L-dopa, a precursor of dopamine. The report has been provided by Ivan Vaughan, a curious, brave, articulate, and extremely intelligent patient, who happened to be a boyhood friend of John Lennon and a schoolmate of Paul McCartney (Ivan was the one who introduced McCartney to Lennon, Vaughan, 1998). The experiment was documented on a video produced by Vaughan and Sir Jonathan Miller, a renowned theater director and neurologist (Menken, 1988). Vaughan has been a proficient snooker player before the onset of the disease. To demonstrate the effect of the medication, he has abstained from L-dopa for an extended period of time before making the movie. In the first, depleted-state round of the experiment, Ivan could not even stand up, approach the snooker table, and take hold of the snooker cue in one step. To accomplish the task, he reports having to split the act into multiple short, roundabout, digressive, actions, nevertheless failing to strike the cue ball. Following the medication, “almost like a ghost exorcized from his body, the tremor slips quietly away.” Ivan then proceeds with a series of exercises using one hand to twist and dorsiflex the fingers of the other hand (Supplementary Figures 5C,D), a type of stretch exercise used by Parkinsonian patients for building up their own mobility (Nasrallah et al., 2019). As he stands up gracefully and cheerfully in front of the snooker table, he is asked by Miller: “you have a different view of this table now to what you had, say, an hour and a half ago?,” to which Ivan responds:” indeed, yes…! It’s now packed with possibilities, achievements, rather than struggles and failure! It is very difficult when I approach the table now, in my new state of control, to remember, sort of emotionally, the difficulties I was having before. The two states of existence are totally separate, and yet, the transition from one to the other is smooth and matter of fact. So, when I take the medication and go over into this state of control, I simply pick the snooker cue, aim at the cue ball, and…” (as Ivan’s precision strike succinctly interrupts his verbal commentary) …“off we go!”…A similar effect is reported by Ivan following trampolining: “I had been off my drugs all day. At three o’clock I launched myself on to a huge trampoline. After a lot of bouncing, falling and springing, I eventually came off and then spent an exciting hour playing football, excitedly rushing around, darting in different directions, all with perfect postural control….”

*Enhancing mobility by stretching* (*Pandiculation*). The arousal, activation, decreased stiffness, and comfort attainment reported by Ivan and other Parkinsonian patients following stretch exercises, also characterize the wellbeing following *pandiculation* - the act of stretching oneself, especially upon waking, common in animals (Supplementary Figures 5A,B and Supplementary Video 28), and in man, intact (Supplementary Figures 5C), or Parkinsonian (Supplementary Figures 5D,E).

This behavior (Fraser, 1989) is common in livestock, involving arching of the neck, or extending the head or a hind leg, or a foreleg. In poultry, the common forms of stretching include vigorous extension of one wing after another. While the concept is of musculature being stretched, many of the above actions clearly involve marked joint extension.

Whereas the stretch’s enabling effect is relatively mild following pandiculation, in Ivan’s case the effect is dramatic. If the quantal phenomenological transformation reported by Ivan is homologous across the vertebrates, then the perceptual and cognitive *presence* and the *meaningful vividness* he reports, sometimes culminated by a precision strike, might also characterize the *umwelt* (von Uexküll, 1957) of our “fellow brethren” be they exuberant stoats at the peak of buildup of their “war dance,” culminated by a precision bite; birds of paradise culminating a buildup of flamboyant courtship with copulation; stotting impalas warming up for an unpredictable lightning-fast zigzagging (“Protean”) flight from a predator; and even human lovers use, according to Carl Jung, of “everybody’s road for accomplishing numinosity” via courtship and sexual foreplay, winding up in coitus (Jung, 2014) and then orgasm (Pavličev and Wagner, 2018). A common precursor of all these buildup and differentiation processes is the experience of intense proprioceptive and/or tactile input that brings about an enabling process accompanied by a quantal jump in exuberance. It would be intriguing to consider the possibility that a human meditation practice, such as the ancient Vipassana (Hart, 2011), is realized through a disciplined flow of attention to one’s own physical sensations in an AP anatomical order along one’s own body, also culminating with a jump in the meditator’s *umwelt*, including a quantal jump in the experience of freedom, a liberation from impinging external and internal stimuli, and the perception of a numinous world pregnant with affordances. Not unlike Ivan, meditation practitioners are also acquainted with “two totally separate states of existence whose transition from one to the other is smooth and matter of fact.”

*Do behavioral phylogenetic innovations grow in a prescribed order along the dimensions of the general SoR and bauplan*? The correspondences between the generative rules that hold in phylogenesis, ontogenesis, and actualgenesis are: (*i*) horizontal precedes vertical, (*ii*) PA heavy-to-light limbs hierarchy of trunk metameres’ movement precedes AP heavy-to-light limbs hierarchy, and (*iii*) transition of propulsive force generation proceeds from Proximal to Distal, and back to Proximal. Does the geometric SoR only portray the existing geometry of behavior, or does it also *prescribe* evolutionary behavioral innovations?

Take, for example, vertebrate phyletic forms of locomotion before the emergence of body-related vertical (sagittal) movement: at that time, 3-dimensional physical space was traversed by vertebrate swimmers using only horizontal *body-related* movement. This state of affairs is currently illustrated by extant fish, amphibian larvae, and the mammalian otter shrew (*Potamogale velox*). At the time, body-related vertical movement has not evolved yet, and vertical body-related movement has been an unrealized dimension, a so-to-speak phylogenetic vacuous potential niche awaiting phylogenetic manifestation.

Body-related vertical movement, manifested as gallop, perhaps emerged first in the course of ontogeny, during high speed forward progression. Manifested in the same animal, side-by-side with low speed horizontal AP waves (Jones et al., 2018, 2021), it perhaps served as the matrix for the full-fledged vertical AP waves, which became the exclusive type of swimming in secondary aquatic vertebrates such as otters (*Lutrinae*) and whales (*Cetacea*). Such mode of evolutionary innovation, whereby “behavioral change comes first” has been offered by Mayr (1958, 1960) cited in Wagner (2014). The behavioral flexibility that characterized terrestrial Tetrapoda was perhaps replaced by a novel behavioral neophenotype, which in turn brought about a morphological innovation in trunk anatomy, followed by genetic integration (Johnston and Gottlieb, 1990 cited in Pigliucci and Muller, 2010).

Swimming by vertical AP movement has been, in a way, both afforded and constrained by physical space, and correspondingly, both foreshadowed and constrained by the linearity and modularity of the behavioral *bauplan* and by the vertebrate’s SoR, not unlike the way that land and air afforded and shaped their invasion by respective innovative life forms. Note, however, that whereas the portrayal of potential phylogenetic affordances and constraints is at best an intuitive, unsystematic art, the behavioral *bauplan* mathematical and quantitative model might have evolved in the footstep of the geometric manifold. As such, the SoR could perhaps be used as a roadmap for the discovery and explanation of past, and perhaps also future, kinematic innovations.

*On the geometry of bird display.* Using the vertebrate’s SoR as a reference, we perform a cursory examination of bird display geometry. An old ethological study (Daanje, 1950) on the presumed evolutionary origins of bird display, suggested that it consisted of low-intensity forms of locomotor behavior, such as stepping, and head, neck, trunk and wings locomotor movements. These movements were described as “intention movements” (Heinroth, 1955) serving as the matrix for display. When preceding a full blown amplitude movement, they were reported as part of a motorial expansion process (Lorenz, 1957; Kortmulder, 1998; Dubbeldam, 2001), or as low-intensity forms of locomotion, similar to the movements performed during the early stages of buildup described in the present essay as part of the mobility gradient. These movements and their derivatives are performed in isolation, and/or in the context of an interaction, and/or *vis-à-vis* congeners, sometimes in an exaggerated form involving repetition, or an increase in amplitude, or, exhibiting regularity in body-related rather than in partner-related frame (Lorenz, 1958). They were described as “ritualized,” i.e., movements that were exapted (co-opted for a use other than the original one) from their original locomotor context and function, sometimes also undergoing a change in form, at times also undergoing amplification due to ornamentation by plumage structure, and intensification of movement, and color, so as to become specialized for communication (Morris, 1957).

The majority of the examples of motor display presented by classical ethologists (Daanje, 1950; Lorenz, 1958) consisted of apparently exapted trunk, neck, and head locomotor movements (ritualized intention movements) performed along the bird’s midsagittal plane (body-related [0–4] plane; vertical movement), including rotations of the neck. To illustrate this observation with but a few examples, the White stork (*Ciconia ciconia*) mainly moves its trunk, neck, and head during courtship in the midsagittal [0] plane (Supplementary Video 29). A major component of the Houbara Bustard (*Chlamydotis undulata*) male display consists of forward locomotion along the midsagittal plane (in [0] plane) (Supplementary Video 30). A major component of the Laysan Albatross (*Phoebastria immutabilis*) display consists of trunk, neck, and head movements in the midsagittal [0] plane (plus neck rotations) (Supplementary Video 31). Duck (Anatidae) courtship behavior also mainly consists of head, neck and trunk plane movements, including forward locomotion, all in the midsagittal [0] plane (plus some neck rotations; Supplementary Video 32). A common denominator of these movement-based display gestures is that they are “made” of (exapted from) existing locomotor movements, and that they unfold along the bird’s body-related AP (midsagittal or vertical) plane.

Intriguingly, an explosion of innovative displays appears in exotic species such as the neotropical manakins (*Pipridae*; Prum, 1990) whose display includes side-to-side progression (and “moon-walking”) along the frontal [2–6] plane, and the birds of paradise (*Paradisaeidae*), a quintessential example of elaborate ornamental diversification among animals, consisting of hardly-ever-seen-in-other-birds movement types, plumage ornaments, colors, and plumage movement (e.g., Frith and Beehler, 1998; Prum and Brush, 2002; Scholes and Laman, 2018). Even a cursory examination reveals that “ritualized” movement in the birds’ midsagittal (body-related vertical) plane also features in the birds of paradise display. But in addition, these birds also exhibit flamboyant movement in novel planes, and around novel axes. For example, see Supplementary Videos 33–37.

One example is the Vogelkop superb bird of paradise (Supplementary Video 33), who performs to-and-fro repetitive side-to-side hopping (0:38–0:43) (Nat Geo WILD, 2018), involving whole-body shifts of weight along the body-related (frontal) side-to-side axis [2–6].

Another example is the western parotia (*Parotia sefilata*), who performs trunk, neck and head plane movement without stepping, in the side-to-side plane (frontal plane) of the trunk, as well as plane movement of the neck in the side-to-side (frontal) plane, with simultaneous fixation of the head in the vertical absolute (the head is maintained in the same position on the frontal [2–6] plane due to antagonistic simultaneous movements of the head and neck on the trunk; Supplementary Video 34).

These and several other displays occupy novel body-related dimensions and/or novel movement types. It is yet to be established whether locomotor behavior is the matrix out of which these innovative display movements were exapted. It is also to be established whether the birds of paradise perform display movements along the midsagittal plane before movement along the other planes in actualgenesis and in ontogenesis. The prevalence of head, neck, and trunk plane movements versus the paucity of conical movements in bird display suggests the evolutionary primacy of plane movements. The fixed order of emergence of dimensions of movement in vertebrate locomotion, and perhaps also in bird display, might disclose evolutionary constraints on the order of innovations along the various spatial dimensions of the general SoR. Concurrent explosion of color novelty, plumage novelty, and movement geometry novelty – the explosion of creativity in all three aspects – is puzzling.

Replacing the label “Perch-pivot” with “horizontal trunk movement” and substituting the category “tracing a Figure 9 “with “trunk conical movement” (Figure 5B, middle) is not simply a stylistic choice: while the first of the two options reifies a list of fragmented building blocks, the second option is literate, in the sense that it highlights a geometric novelty, embedded within the geometric manifold of the general SoR, relating in this way to the whole universe of already enacted as well as prospective, yet to be evolved, novel movements. Geometrizing behavior instantly puts the birds of paradise display movements on the same manifold and scale with all movement-based bird display, with all bird movement, and with the entire manifold of vertebrate movement, prompting a quantitative phyletic comparison of the morphogenesis of kinematic form in evolution. The envisaged aim of such endeavor would be an evolving morphogenetic tree whose main trunk would consist of the six spatial locomotor dimensions portrayed in this essay and its branching out offshoots would consist of growing and differentiating boughs - one of courtship culminating in sex, another of foraging culminating in eating, etc.

*The geometrization of movement and of other related phenomenal manifolds.* As illustrated by the story of Funes the memorious, the insight that there are such entities as quantity and order is a prerequisite for the realization that there is such thing as a describable arithmetic manifold. Numeral literacy, the ability to read, write, and calculate with numerals, is the technological tool for exploring and mapping this manifold. Similarly, the insight that movement-based behavior is essentially continuous in the same way as anatomy, has been the impetus for the development of a technology of movement literacy, which can in turn be used to portray the geometric manifold of animal movement, i.e., the geometric *bauplan* of growth and decay of animal-movement form.

A geometrization of animal movement-based behavior in organisms whose skeletal body plan consists of an articulated linkage of rigid segments requires the reduction to geometric constructs of such kinematic phenomena as locomotion, exploration, fighting, play, courtship, etc. To achieve this aim we must find geometric reference frames, origins, primitives, and modules, in which different geometric properties stand to each other in the same formal relation as the different kinematic phenomena stand to each other in real articulated organisms. In my own work, having been caught by a technology of description that fragmented the behavior irrevocably (Golani, 1973), Ilan Golani was fortunate to encounter a literate technology that forced the user to see the common geometric manifold on which the behaviors unfolded in morphogenesis. The initial conditions for such study were favorable: a phenomenological technology of description like EWMN is rare, and movement is fully accessible for observation (as opposed to, e.g., neurophysiological, perceptual, or thought processes), and the natural frames, origins, etc., could be readily established. These geometrical constructs were used to replace the particularistic, idiosyncratic, units of behavior, which are *not* the primitives shared by all the members having the same *bauplan,* with topologically defined, phylogenetically shared, primitives.

As articulated by Rashevsky (1956) the only objectively scientific study of life can be made through the study of its physical manifestations. If physics is to be reduced to geometry, then it follows that eventually a geometrization will be also the fate of behavior. As demonstrated in the current essay, the invariant aspects of anatomy and movement are relational, therefore the geometrization of behavior has to be topological.

The current essay copes with the geometrization of movement-based behavior, revealing correspondences between physical space, situatedness in physical space (e.g., the heavy and light limbs hierarchy), and life-space. It would, therefore, be intriguing to examine how the challenge of geometrization of related landscapes, such as those of perception, language, and thought have been coped with, and what kinds of findings did such coping yield. The proposition that thinking is more of a movement than normally supposed is not far-fetched, being expressed in the embodied mind thesis, which says that cognition depends upon the kinds of experience that come from having a body with various sensorimotor capacities (Varela et al., 2017), and in the demonstrated claim that basic sensorimotor schemas pervade abstract language, thought, and action, with bodily, orientational, and spatial metaphors (Lakoff and Johnson, 2008). In a review on “Order and form” Gould quotes d’Arcy (1917): “The study of form may be descriptive merely, or it may become analytical. We begin by describing the shape of an object in the simple words of common speech: we end by defining it in the precise language of mathematics… The mathematical definition of a “form” has a quality of precision, which was quite lacking in our earlier stage of mere description; it is expressed in a few words or in still briefer symbols, and these words or symbols are so pregnant with meaning that thought itself is economized; we are brought by means of it in touch with Galileo’s aphorism (as old as Plato, as old as Pythagoras, as old perhaps as the wisdom of the Egyptians) that “the Book of Nature is written in characters” (Gould, 1971). In his dialogue “Timaeus” Plato pleaded for a geometric foundation of natural laws including laws of the human mind (Wildgen and Brandt, 2010). Thom (1990) maintained that the geometrization of cognition and language was already largely achieved by Aristotle. The philosophy of symbolic forms conceived and developed by Cassirer (1979) compared different types of cognitive grip humans have on reality. A scale of increasingly more rational apprehension of reality links the symbolic forms: myth > language > science (mathematics). In myth, the distance between the cognitive capture and reality is minimal; in language, larger; and in the mathematical sciences (e.g., theoretical physics) maximal, yielding an architecture that encompasses major aspects of reality, a greater capacity of generalization, and possibly a global understanding (the hidden kernel of reality; Wildgen and Brandt, 2010).

A critical question is which mathematical tools to use in order to geometrize the phenomenal manifold in a respective discipline. In the study of movement-based behavior we were fortunate to be entrusted with a tool mirroring the structure and connectedness of the body plan, relating movement to both the body and the environment (body-related and absolute frames), highlighting the mechanical constraints on kinematic linkages (heavy and light limbs hierarchy), providing a phenomenological account by disclosing perceptual invariants and hierarchies of attention, using well-defined primitives and an open symbolic system capable of describing generatively unforeseen phenomena, all in the relational, quantifiable language of geometry and topology.

A complex form cannot be described without first isolating its discontinuities. Importantly, EWMN takes care of the multiplicity of discontinuities by identifying the boundaries between single movements, the boundaries between modules and between chords of simultaneous movement, and the points of change in the base(s) of support during movement, engendering a change in the mechanical dependency between the segments.

*Features revealed in the geometrization of the phenomenal manifold of movement-based behavior.* The continuity of movement of organisms endowed with a skeletal linkage is partitioned into skeletal-segments’ movement primitives. A primitive is a continuous arc, bounded by discontinuous positions, traced by the “light” end of a rigid segment on the envelope of its individual spherical SoR, centered on its “heavy” joint. The angular change between the light segment and the heavier segment on which it moves is unequivocally described by a single notational expression. The locomotor primitives are recruited in vertebrates during the buildup of movement in a linear AP order, separately along each of six distinct spatial modules. The behavioral *bauplan* formed by these modules folds in stressful situations in reverse, last in first out. Movement unfolds in moment-to-moment morphogenesis and in ontogeny, day by day, in the same order. The linearity of recruitment within modules mirrors the mechanical kinematic constraints on the linkage. The modules unfold in a prescribed order in both vertebrates and arthropods, and fold in the opposite order, under stress, in both phyla. Continuous dynamics prevails within spatial modules, while discontinuities occur – and are bridged by intercalation of types belonging to successively enabled modules.

The continuity within the physical dimensions is re-unfolded in behavior by preserving the integrity of each of the mirrored dimensions in the re-unfolded module. A seamless transition across the discontinuities, mirroring the physical discontinuities between physical dimensions, is accomplished by intercalating the movement primitives belonging to the multiple modules in a lawful (module-specific AP) order along the kinematic sequence. Each of the spatial kinematic modules is integrated in this way into the exploratory flow while on the one hand, maintaining its integrity through the modular bookkeeping, and on the other hand, weaves a seamless spatial kinematic continuity. The intentionality of the animal - distinguishing between the dimensions while at the same time not mixing their identities - is instantiated in the sequential rule of the mobility gradient.

The syntax of the mobility gradient (horizontal first, forward second, etc.), reflects the” natural” dynamic order in which discontinuities are typically encountered by an organism: proceeding from immobility, and implementing horizontal movement for proximal space, forward movement for more distal space, vertical scanning movement against vertical surfaces, vertical on hind legs in the air *vis-à-vis* the horizon, and whole body in the air. The body facet, the space facet, and the syntax facet alike, thus mirror the order in which reality presents itself to the organism: what has been described as early as 1913 as “The fitness of the environment” (Henderson, 1913). The observation that the behavioral spatial modularity echoes the discontinuity outside the organism implies that the physical dimensions carry a biological significance for the organism.

These observations were made years before Ilan Golani was exposed to Thom’s views on the role played by physiological modules (bodily organs) in bridging across metabolic discontinuities (so-called “catastrophic” jumps), expanding thereby the organism’s life space (Thom, 1982; Bundgaard and Stjernfelt, 2010).

*Addressing discontinuities in perception, thought, and language.* A premiere in developing mathematical tools for the geometrization of perception, thought and language – phenomena, all involving a mixture of continuities and discontinuities has been Rene Thom (Tsatsanis, 2012; Thom, 2018). Thom offered the methodology and tools of Catastrophe (discontinuity) theory for handling morphogenetic divergences. In “Semio Physics: a sketch” (Thom, 1990), he offered a comprehensive outline of a methodological solution for the problem of geometrizing thought and linguistic activity (Thom, 2018). In that book he has been concerned with the topological description of physical gestalts perceived by the organism (“saliencies”) and with their biological operational significances (“pregnancies”). The topology of the physical gestalts, along with their respective affordances, constitutes the intelligible modules of perception, action, cognition, and language. In Thom’s geometric description each of the modules is represented by one out of seven prototypical manifolds, called “elementary catastrophes,” which include a sudden shift in behavior. For processes involving four factors (e.g., the three spatial dimensions and time), there are seven elementary catastrophes, each portraying the architecture of the object and its operational significance in perception, action, thought, and language.

*Features revealed in the geometrization of the phenomenal manifold of perception, cognition and language.* Thom (1990), supporters (Largeault and Thom, 1988; Wildgen and Brandt, 2010; Petitot, 2011), and cognitive linguists (e.g., Tesnière, 1959; Talmy, 1978; Jackendoff, 1983; Langacker, 1987) propose mathematical and schematic qualitative ontologies (lists of the things that exist) of the physical world, of human perception, and of cognition and language. Resonating with the compatibility we describe between physical space and modular life-space, they stress the compatibilities of language with perception and action, see cognitive grammar as a perceptually rooted “space grammar,” criticize the mechanistic conception of grammars as algorithms that generate languages from finite sets of rules, and consider meaning and grammar as indissociable (a central claim of cognitive grammar, Talmy, 1978; Jackendoff, 1983; Petitot, 2011). These authors question the autonomy of syntax and claim that there exist semantic constraints that condition syntax, where these constraints are themselves constrained by the structure of perception (Jackendoff, 1983). In the same way that the dimensionality of physical space impregnates the dimensionality of life-space (current essay), these authors advocate looking for the roots of basic linguistic structures in the relations between the active subject and reality and not in the mind itself (Luria, 1974). Thom’s definition of a morphology as a system of boundaries, and the modular boundaries in life-space demonstrated in our work, which correspond to the dimensional boundaries in physical space, fit with Langacker’s description whereby human scanning picks up qualitative discontinuities and builds from them a schematic imagery which is at the same time linguistic and perceptual. Cognitive scanning - the detection of qualitative discontinuities in general qualitative spaces - opens onto a *geometrization of meaning* (Langacker in Petitot, 2011). Thom, and following him Petitot, thus postulate that the universals of language are rooted in cognitive universals which are themselves dependent on the qualitative morphological structure of the natural phenomenal world. In both the realm of movement and the realms of perception, cognition and natural language, the issue of a corresponding order across levels is not addressed in terms of an arbitrary link between signifier and signified, but in terms of a re-articulation of one order in another order. In both our and Thom’s (Petitot, 2011) universes of discourse, to perceive is to take in a form from one domain, the physical realm, a form, which is then re-unfolded in another realm or domain. Taking in the physical dimensions and the mechanical constraints of the body and re-unfolding them in movement (in our case), or taking in the physical dimensions and the body and re-unfolding them in the mind (in, e.g., Thom’s and Petitot’s case; and in Lakoff and Johnson’s; Lakoff and Johnson, 2008) case.

*Postscript* [by Ilan Golani]: Some 30 years ago, having demonstrated that a literate analysis of the morphogenesis of vertebrate locomotor behavior reveals a mobility gradient, which constitutes a geometric homology (Golani, 1992a, 1992b), My endeavor was applauded by Rene Thom: “Note that in his theorizing, Golani (without making it explicit) draws heavily on facts that are essentially mathematical, arising, as they do, from mechanical constraints. Yet no equations are written or solved, only plain ordinary language is used. This does not render Golani’s account less convincing, although the use of mathematics is only qualitative here. I hope this work will serve as an example. In its methodological aspect, the importance of Golani’s article can hardly be overrated” (Thom, 1992). At the other extreme, the response of ethologists and philosophers to the focus on kinematic literacy was apprehensive: “Golani went over the brink with his meticulous descriptions of mammalian social interactions—and the ethological community balked” (Drummond, 1985); “It was like reading a newspaper with a microscope” (Barlow, 1992); “A danger of EW notation is that it obscures the function driven nature of … the evolutionary process”…. (Allen, 1992); “the assessment of homology on the basis of the principle of connections, independent of phylogeny, seems odd nearly 140 years after the publication of *The Origin of Species”* (Lauder and Hall, 1994), and so forth (Golani, 1992a).

Thirty years later, automatic marker-less video-tracking of all skeletal joints in vertebrates and arthropods (Mathis et al., 2018) is accomplished using deep learning technologies (LeCun et al., 2015), making the kinematic analysis we have proposed readily accessible. The results render the reluctance to use kinematic analysis outdated, foreshadowing easily accessible, massive amounts of kinematic data (von Ziegler et al., 2021). A meaningful analysis of such data - derived from the movement of unrestrained (free) kinematic linkages - requires, however, taking into account their intrinsic connectedness. An unavoidable way to get out of this situation would be the use of the principles adopted in our movement notation analysis for the portrayal of a fully quantitative geometric manifold, in the same way that numeral literacy paved the way for, e.g., grand scale computation. This implies a preparation of the data using the principles employed in EWMN (Golani, 1976; Eshkol and Harries, 1998), thus securing the geometrization of movement-based behavior.

The several decades that have passed since the nascence of a literate study of animal movement as well as the renewal of the extensive study of comparative developmental anatomy allow us to identify shared questions, methodological principles, and results, obtained in the respective fields. This will prompt behavioral biologists to study the morphogenesis of behavior, and entice geneticists to look into the processes that mediate this behavior. Both fields strive to explain the organization of the phenotype by formulating the generative rules of its morphogenesis; both fields use monsters, actual genesis, ontogeny, and recovery, to extract the invariants that shape the essential core of the phenomenon while temporarily suspending judgment about adaptive explanations; and both fields uncover a morphospace characterized by few, distinct, relatively separated modules that unfold, following a multiplicity of treatments and situations, in an invariant linear and modular order.

The study of the pyramid of life requires an understanding of both the bottom-up, and top-down relationships across levels. Animal life implies action, and the main component of action is movement. Using literacy, the level of movement (skeletal kinematics) is as tangible as anatomy, disclosing to the observer what is relevantly the matter. A mapping of the geometry of the behavioral *bauplan* can perhaps serve as a Rosetta stone for deciphering the other, less tangible levels, including aspects of the brain, perception, attention, cognition and perhaps also language.

A century after the publication of D’Arcy Thompson’s monumental work “On growth and form” (d'Arcy, 1942; Gould, 1971), which geometrized aspects of animal morphology in a grand way, the current essay geometrizes the *bauplan* of the growth and form of a part of animal behavior. This also yields the geometrizing of aspects of perception, attention, emotion, and cognition, if only in a primordial way, yet leading all the way to experiences relating to numinosity, and an *umwelt* pregnant with presence, freedom, and joy.

## Data Availability

The original contributions presented in the study are included in the article/Supplementary material, further inquiries can be directed to the corresponding author.

## In memoriam

IG was originally the sole author of this manuscript, but regretfully passed away before completing the submission process. NK had assisted IG with writing and preparation of the manuscript for publication, and is now added as an author, to enable the submission process, and edit any revisions as needed.

## Supplementary material

Supplementary Figure 1 **(A)** Honey badger’s (Melivora capensis) whole-body horizontal movement around the vertical axis located in the hindquarters (emerges early in morphogenesis). **(B)** Whole-body horizontal movement around the vertical axis located in the forequarters (derived). **(C)** Whole-body vertical movement head-on (clockwise rotation around the side-to-side axis of the body when viewed from the right side; derived). Whole-body rotation in the opposite, counterclockwise direction involving saltation pelvis-on may be performed when the animal maintains snout to shoulder opposition with a partner moving in the opposite direction (after Yaniv and Golani, 1987).

Supplementary Figure 2 The hydra’s body plan, with a hypothesized operational space represented by several vertical planes centred on the main body axis (see text).

Supplementary Figure 3 Snapshots of the two extremes in the middle of the buildup and narrowing of contact-with-ground-and-support module. **(A)** “Passive submission” in wolves illustrates maximal narrowing of support and maximal contact with ground (from Schenkel, 1948; With permission of Brill). **(B)** An artist’s illustration of “the big bad wolf” in a children’s book portrays “active submission”: a back to front narrowing of support and increase of feet contact with ground; **(C)** Stotting in Thompson’s gazelle – an exuberant behavior performed at the peak of the mobility gradient buildup. It illustrates simultaneous release of all four feet contact with ground; performed by antelopes upon noticing a coursing predator (from Sebeok, 1977), reproduced by permission of Hachette Children’s Books, Carmelite House, 50 Victoria Embankment, EC4Y 0DZ. In **(A)** and less so in **(B)** the animal’s movement repertoire that follows the respective posture is narrow, while contact and opposition stimuli induce stereotyped, predictable, and stimulus bound behavior. In contrast, in **(C)**, stotting is sometimes followed by so-called Protean behavior, involving unsystematic, unpredictable path, including zigzagging, spinning, looping, or bouncing, claimed to impede the predator’s prey catching (Humphries and Driver, 1970).

Supplementary Figure 4 Steady maintenance of a particular relationship of opposition (near contact) of a Tasmanian devil’s (*Sarcophylus harisii*) snout vis-à-vis the snout of its partner, during a ritualized fighting interaction (Eisenberg and Golani, 1977).

Supplementary Figure 5 Building up mobility by stretching the kinematic linkage, in animals and man in health and disease. and: pandiculation upon waking, including stretching and yawning, in cat **(A,B)** and man **(C)**, Permissions and image licenses have been obtained from the copyright holders (Source: Shutterstock.com); Finger stretching enhances mobility in a Parkinsonian patient preparing himself for playing snooker **(D,E)** from Ivan (1984) BBC (Miller Vaughan) **(D,E)** are unfortunately not included in this submission, since the BBC “studios learning” does not answer any of my emails requesting permission to use them. I still hope to get them for the final submission, and in the meantime, they can be watched in the preprint: https://osf.io/preprints/osf/mqeg3.
