# Supplementary figures and images for: On growth and form of animal behavior
Source: Front Integr Neurosci. 2025 Feb 4;18:1476233. doi: 10.3389/fnint.2024.1476233 (PMC11832518; doi:10.3389/fnint.2024.1476233)

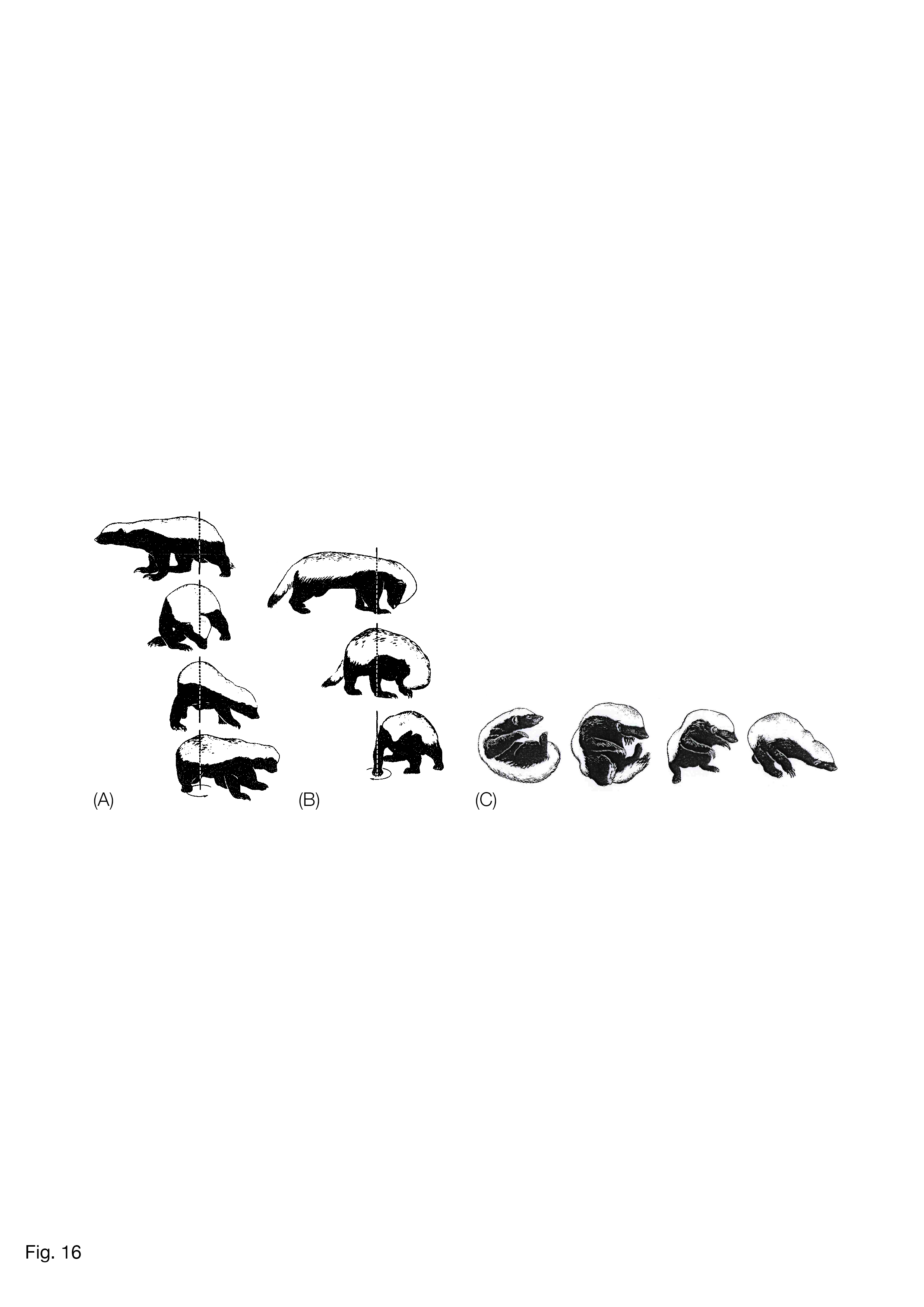

Supplement: Supplementary Figure 1 — (A) Honey badger’s (Melivora capensis) whole-body horizontal movement around the vertical axis located in the hindquarters (emerges early in morphogenesis). (B) Whole-body horizontal movement around the vertical axis located in the forequarters (derived). (C) Whole-body vertical movement head-on (clockwise rotation around the side-to-side axis of the body when viewed from the right side; derived). Whole-body rotation in the opposite, counterclockwise direction involving saltation pelvis-on may be performed when the animal maintains snout to shoulder opposition with a partner moving in the opposite direction (after Yaniv and Golani, 1987). [file Figure_1.jpg]

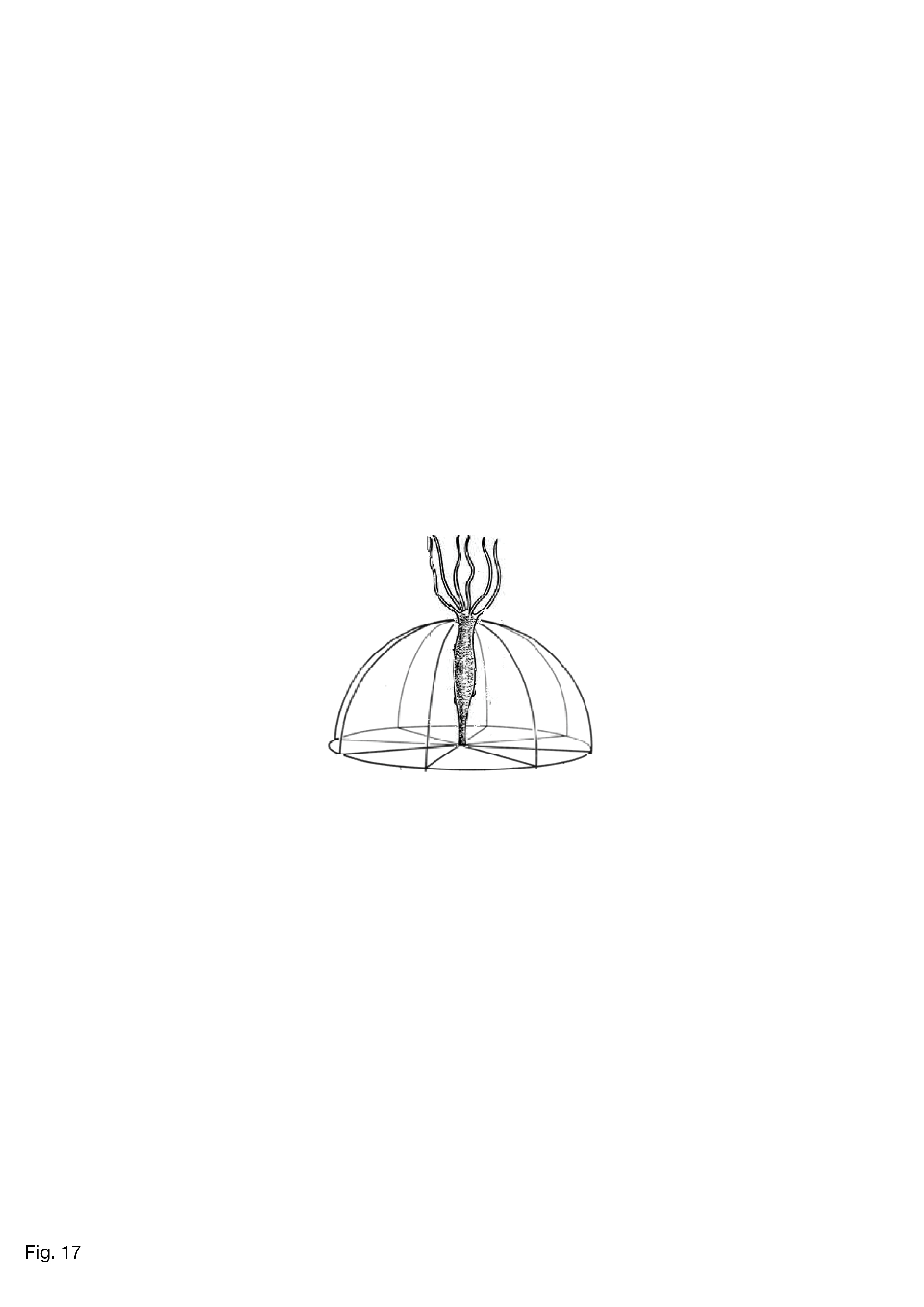

Supplement: Supplementary Figure 2 — The hydra’s body plan, with a hypothesized operational space represented by several vertical planes centred on the main body axis (see text). [file Figure_2.jpg]

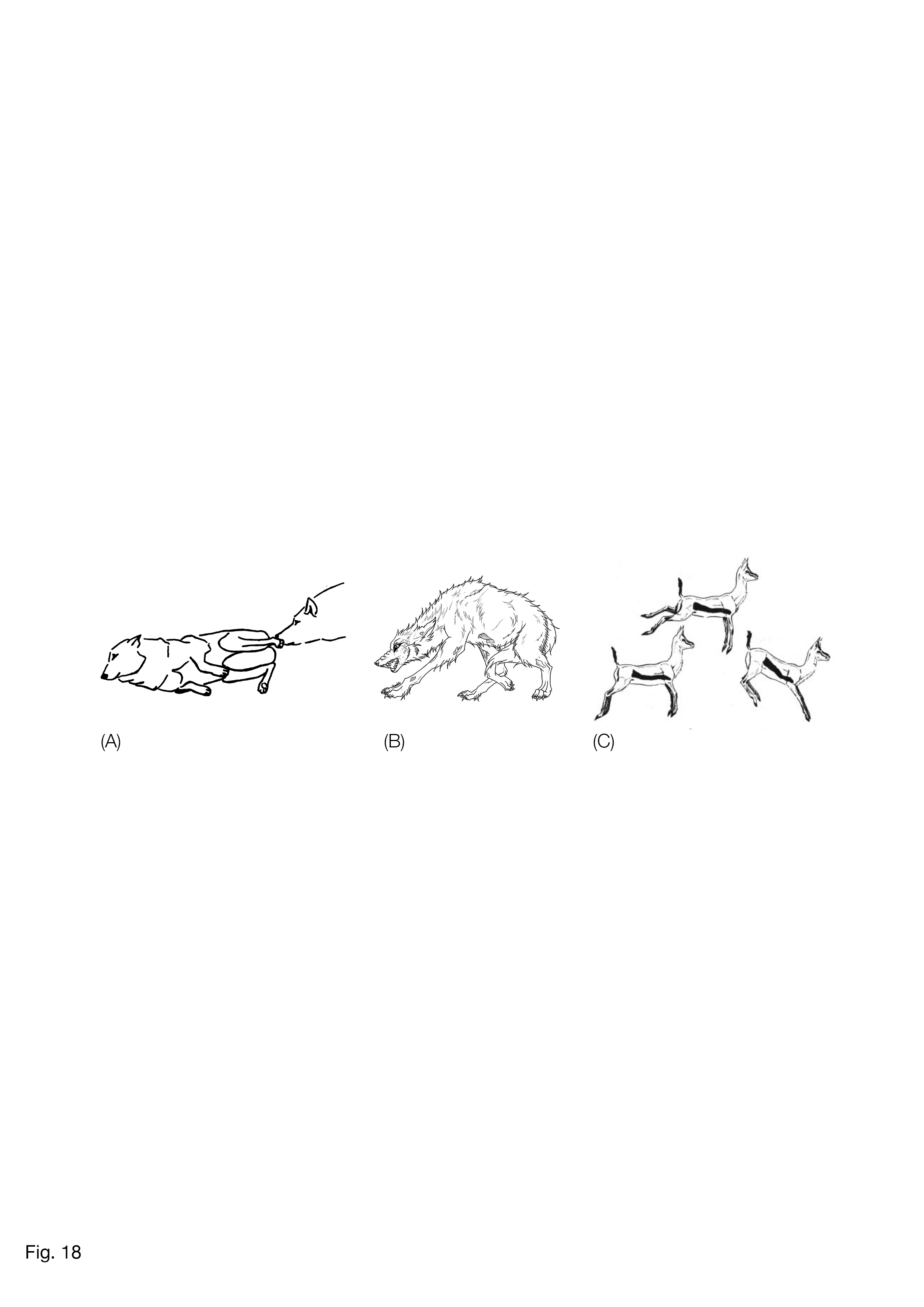

Supplement: Supplementary Figure 3 — Snapshots of the two extremes in the middle of the buildup and narrowing of contact-with-ground-and-support module. (A) “Passive submission” in wolves illustrates maximal narrowing of support and maximal contact with ground (from Schenkel, 1948; With permission of Brill). (B) An artist’s illustration of “the big bad wolf” in a children’s book portrays “active submission”: a back to front narrowing of support and increase of feet contact with ground; (C) Stotting in Thompson’s gazelle – an exuberant behavior performed at the peak of the mobility gradient buildup. It illustrates simultaneous release of all four feet contact with ground; performed by antelopes upon noticing a coursing predator (from Sebeok, 1977), reproduced by permission of Hachette Children’s Books, Carmelite House, 50 Victoria Embankment, EC4Y 0DZ. In (A) and less so in (B) the animal’s movement repertoire that follows the respective posture is narrow, while contact and opposition stimuli induce stereotyped, predictable, and stimulus bound behavior. In contrast, in (C), stotting is sometimes followed by so-called Protean behavior, involving unsystematic, unpredictable path, including zigzagging, spinning, looping, or bouncing, claimed to impede the predator’s prey catching (Humphries and Driver, 1970). [file Figure_3.jpg]

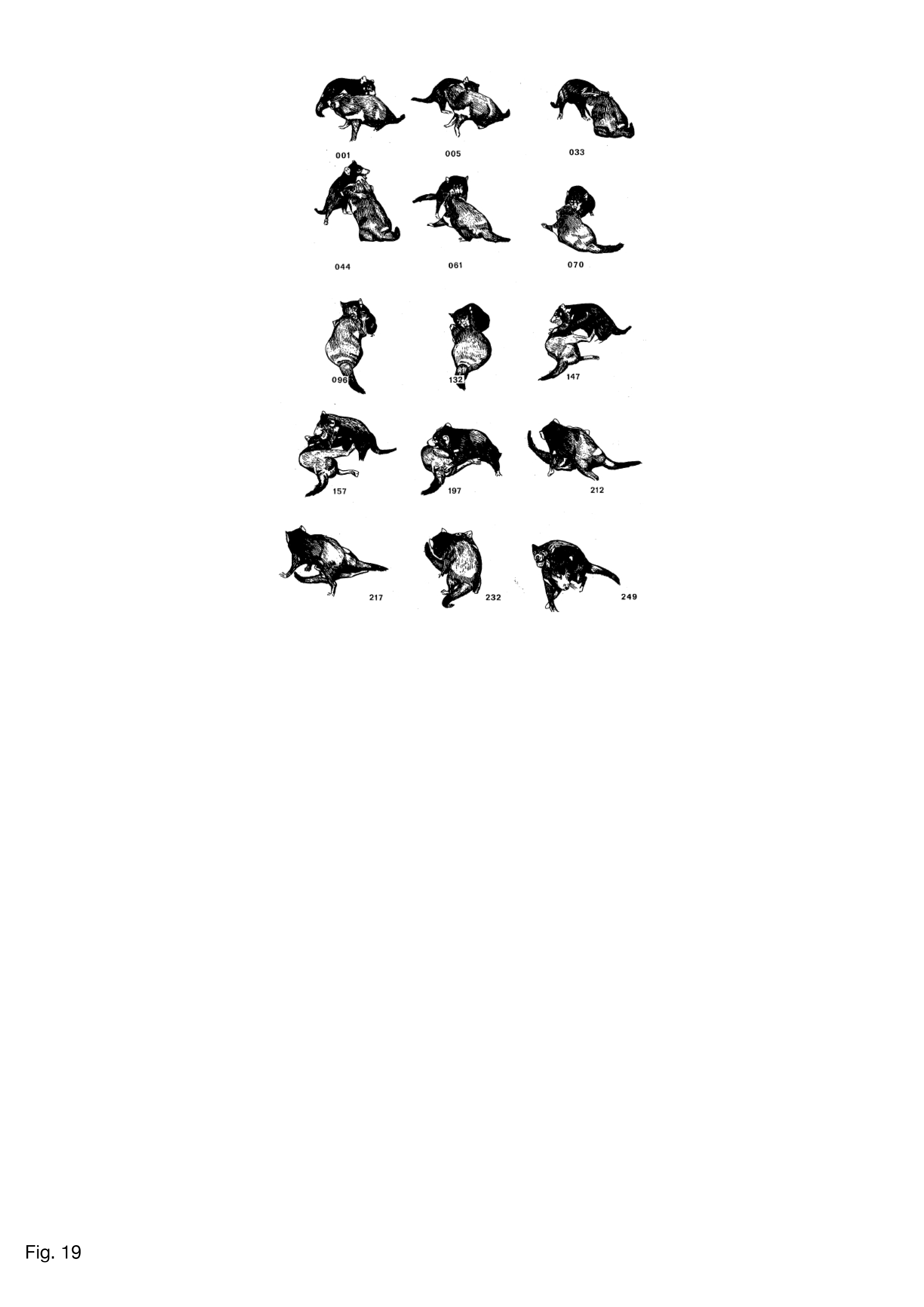

Supplement: Supplementary Figure 4 — Steady maintenance of a particular relationship of opposition (near contact) of a Tasmanian devil’s (Sarcophylus harisii) snout vis-à-vis the snout of its partner, during a ritualized fighting interaction (Eisenberg and Golani, 1977). [file Figure_4.jpg]

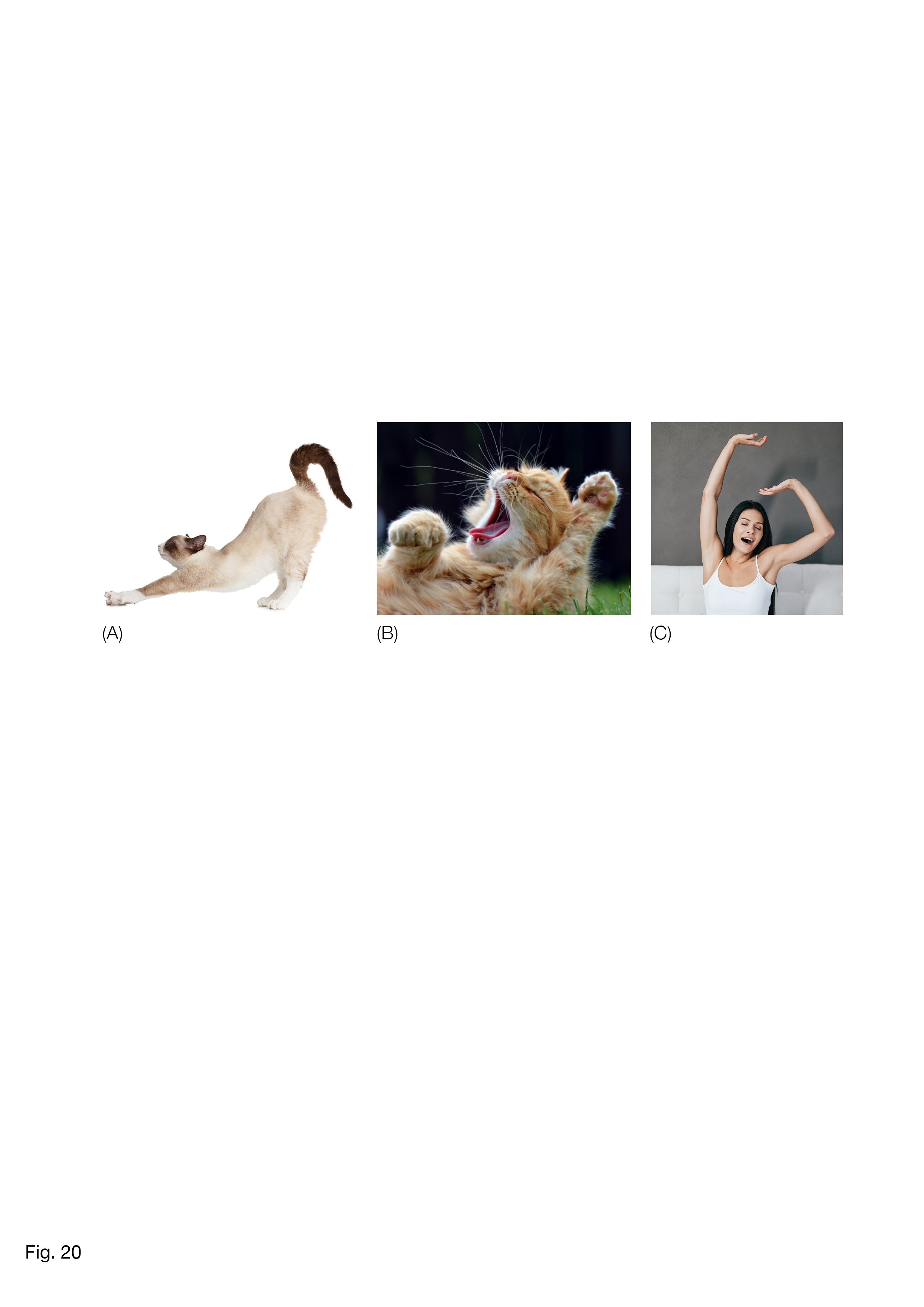

Supplement: Supplementary Figure 5 — Building up mobility by stretching the kinematic linkage, in animals and man in health and disease. and: pandiculation upon waking, including stretching and yawning, in cat (A,B) and man (C), Permissions and image licenses have been obtained from the copyright holders (Source: Shutterstock.com); Finger stretching enhances mobility in a Parkinsonian patient preparing himself for playing snooker (D,E) from Ivan (1984) BBC (Miller Vaughan) (D,E) are unfortunately not included in this submission, since the BBC “studios learning” does not answer any of my emails requesting permission to use them. I still hope to get them for the final submission, and in the meantime, they can be watched in the preprint: https://osf.io/preprints/osf/mqeg3. [file Figure_5.jpg]
